# Thioketone-directed rhodium(I) catalyzed enantioselective C-H bond arylation of ferrocenes

**DOI:** 10.1038/s41467-019-12181-x

**Published:** 2019-09-13

**Authors:** Zhong-Jian Cai, Chen-Xu Liu, Qiang Wang, Qing Gu, Shu-Li You

**Affiliations:** State Key Laboratory of Organometallic Chemistry, Center for Excellence in Molecular Synthesis, Shanghai Institute of Organic Chemistry, Chinese Academy of Sciences, 345 Lingling Lu, Shanghai, 200032 China

**Keywords:** Asymmetric catalysis, Homogeneous catalysis, Synthetic chemistry methodology

## Abstract

Planar chiral ferrocenes have received great attention in both academia and industry. Although remarkable progresses have been made over the past decade, the development of efficient and straightforward methods for the synthesis of enantiopure  planar chiral  ferrocenes remains highly challenging. Herein, we report a rhodium(I)/phosphonite catalyzed thioketone-directed enantioselective C-H bond arylation of ferrocenes. Readily available aryl iodides are used as the coupling partners in this transformation, leading to a series of planar chiral ferrocenes in good yields and excellent enantioselectivities (up to 86% yield, 99% *ee*). Of particular note, heteroaryl coupled ferrocenes, which are difficult to access with previous approaches, can be obtained in satisfactory results.

## Abstract (web summary)

The development of straightforward methods for the synthesis of planar chiral ferrocenes remains highly challenging. Here, the authors report a rhodium(I)/phosphonate-catalyzed thioketone-directed enantioselective C-H bond arylation reaction for the synthesis of chiral planar ferrocenes.

## Introduction

**Fig. 1:**
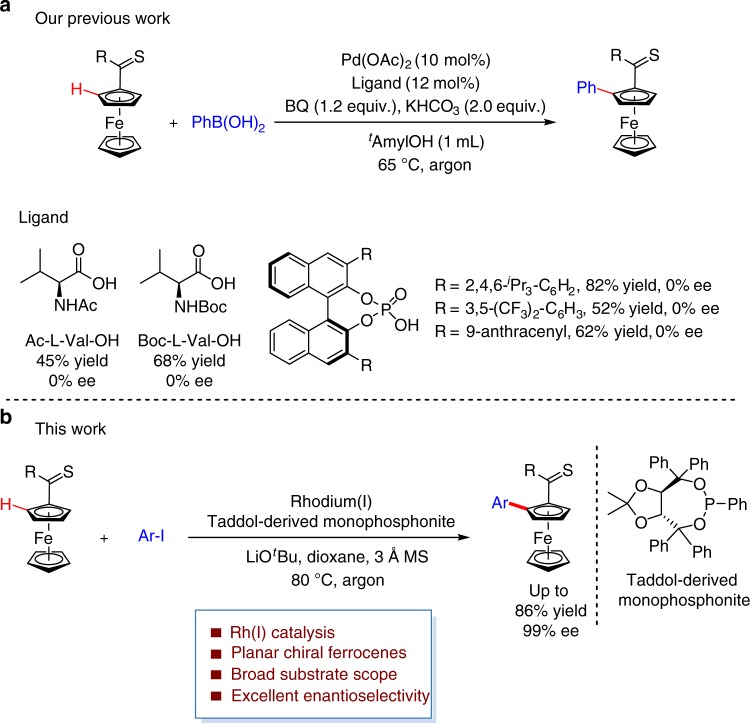
Thiocarbonyl-chelation-assisted C–H bond arylations. **a** Failed attempts via palladium(II) catalyzed direct C–H arylation. **b** Enantioselective C–H arylation of ferrocenes enabled by rhodium(I) and monodentate phosphonite

Transition-metal-catalyzed inert C–H bond direct functionalization has become a powerful tool in modern synthetic chemistry^1–17^. One of the remained challenges for the widespread application of C–H bond functionalization is how to control the regioselectivity and stereoselectivity. Up to now, extensive attention has been paid on this field of research and significant results have been achieved^18–22^. Recently, we developed a Pd(II)-catalyzed C–H direct arylation of ferrocenes with thioketones as effective directing groups^23^. The importance of planar chiral ferrocenes^24–27^ prompted us to explore a corresponding enantioselective arylation reaction. In this regard, Pd(II)/monoprotected amino acid (MPAA) catalytic system^28–36^, established by Yu and coworkers, was firstly examined as our initial attempts for this asymmetric C–H bond arylation. The desired product could be obtained but without any enantioselectivity. Chiral phosphate anion as a counteranion with palladium has also been demonstrated as an effective catalytic system in a number of asymmetric C–H bond functionalization reactions^37–41^. Unfortunately, no positive results on enantioselective control were obtained when various chiral phosphoric acids (CPAs) were introduced (Fig. 1a). Therefore, the development of enantioselective C–H arylation reaction to provide structurally diverse planar chiral ferrocenes is highly desirable^42–61^.

Recently, Rh(I)-catalyzed enantioselective C–H functionalizations progressed rapidly. In 1997, Murai and coworkers reported Rh(I)-catalyzed asymmetric C–H/olefin coupling reaction with modest enantioselectivity^62^. Later, Ellman, Bergman and coworkers achieved a remarkable progress on Rh(I)-catalyzed enantioselective intramolecular C–H alkylation reaction, which gave cyclic products with up to 96% *ee*^63^. Several elegant Rh(I)-catalyzed asymmetric intramolecular dehydrogenative silylations have been demonstrated by the groups of Takai^64,65^, He^66^, and Shibata^67^, respectively. Notably, Glorius and coworkers have made breakthroughs that the combination of a rhodium(I) precatalyst with chiral N-heterocyclic carbene (NHC) or monodentate phosphonite ligand enabled highly enantioselective Csp^3^–H arylations^68,69^.

Inspired by these pioneering results, we explore Rh(I) catalyzed enantioselective arylation of thiocarbonylferrocenes (Fig. 1b) after achieving the synthesis of axially chiral heterobiaryls via isoquinoline directed C-H functionalization^70^. In the presence of Rh(I) catalyst derived from a chiral phosphonite, direct arylation of thioketone substituted ferrocenes with readily available aryl iodides proceeds in excellent enantioselectivity. Herein, we report the results of this study.

## Results

### Optimization of reaction conditions

We began our initial attempts by utilizing chiral diphosphines **L1**-**2** (BINAP, DIOP) as the ligand. However, no product was observed when thiocarbonylferrocene **1a** was treated with 1.1 equiv of iodobenzene **2a** in the presence of 5 mol% [Rh(C_2_H_4_)_2_Cl]_2_ and 3.0 equiv of LiO^*t*^Bu in THF at 80 °C (Table 1, entries 1–2). When Feringa ligand **L3** was used, to our delight, planar chiral product (**3a**) was obtained in 19% yield with 88% *ee* (Table 1, entry 3). Stereochemical induction is predominantly determined by the BINOL backbone of the phosphoramidite ligand by comparing the results of that of diastereomeric ligand **L4** (Table 1, entry 4, 15% yield, −71% *ee*). The utilization of TADDOL-derived phosphoramidite **L5** could further increase the yield to 29% (Table 1, entry 5). Notably, **3a** was isolated in 46% yield and 90% *ee* when TADDOL-derived phosphonite **L7**, introduced by the Glorius group^69^, was used (Table 1, entry 7). Promoted by these encouraging results, we further investigated other TADDOL-derived phosphonite ligands (Table 1, entries 8–13). It was found that the chiral backbone assembled with 2-naphthyl groups delivered **3a** in 26% yield with 91% *ee* (Table 1, entry 8). Further evaluation of substituents [Ar = 3,5-(CF_3_)_2_C_6_H_3_ and 3,5-(^*t*^Bu)_2_C_6_H_3_] on the TADDOL backbone revealed that both ligands could promote the reaction (33% and 44% yield, respectively) albeit with decrease of enantioselectivity (Table 1, entries 9–10). Ligand **L11** bearing a ^*t*^butyl group could not promote this reaction (Table 1, entry 11). On the other hand, varying the aromatic moiety on the P atom afforded product **3a** with comparative results (45–46% yields, 90% *ee*) as those of **L7** (Table 1, entries 12–13). Thus, the easily accessible ligand **L7** was chosen as the optimal one. The addition of molecular sieves was found to increase the yield of **3a** (Table 1, entry 14, 59% yield with 92% *ee*). Increasing the amount of iodobenzene **2a** to 1.3 equivalents also has a positive influence on the reaction outcome (Table 1, entry 15, 78% yield, 94% *ee*). Other molecular sieves did not give a noticeable improvement of the yield. Various solvents were next screened, and it was found that the reaction in dioxane afforded **3a** in 76% yield with 97% *ee* (Table 1, entry 18, see the Supplementary Table 1 for complete optimization). The ratio between Rh(I) and ligand had a profound effect on the results. The use of 20 mol% of **L7** ([Rh]/L = 1/2) provided **3a** without erosion in term of yield and enantioselectivity (Table 1, entry 17, 75% yield, 97% *ee*). The loading of **L7** could be further reduced to 15 mol % (Table 1, entry 18, [Rh]/L = 1/1.5), 76% 97% *ee*). However, both yield and enantioselectivity were decreased when 10 mol% of **L7** ([Rh]/L = 1/1) was used (Table 1, entry 19).

**Table 1:** Optimization of the reaction conditions^[a]^


Entry	Ligand	Solvent	Additive	Yield (%)^[g]^	*ee* (%)^[h]^
1	**L1**	THF	–	0	–
2	**L2**	THF	–	0	–
3	**L3**	THF	–	19	88
4	**L4**	THF	–	15	−71
5	**L5**	THF	–	29	84
6	**L6**	THF	–	23	86
7	**L7**	THF	–	46	90
8	**L8**	THF	–	26	91
9	**L9**	THF	–	33	59
10	**L10**	THF	–	44	12
11	**L11**	THF	–	trace	–
12	**L12**	THF	–	45	90
13	**L13**	THF	–	46	90
14	**L7**	THF	3 Å MS	59	92
15^[b]^	**L7**	THF	3 Å MS	78	94
16^[b]^	**L7**	dioxane	3 Å MS	76	97
17^[b,c]^	**L7**	dioxane	3 Å MS	75	97
18^[b,d]^	**L7**	dioxane	3 Å MS	76^[e]^	97
19^[b,f]^	**L7**	dioxane	3 Å MS	67	94

[a] Reaction conditions: **1a** (0.2 mmol), **2a** (0.22 mmol), [Rh(C_2_H_4_)_2_Cl]_2_ (5 mol%), ligand (0.06 mmol), LiO^*t*^Bu (0.6 mmol) in solvent (1.5 mL) at 80 °C. [b] **2a** (0.26 mmol). [c] **L7** (0.04 mmol). [d] **L7** (0.03 mmol). [e] The diarylation product was obtained in 13% yield. [f] **L7** (0.02 mmol). [g] Yield of isolated product. [h] Determined by HPLC analysis

### Substrate scope

Under the above optimized reaction conditions [**1a** (0.2 mmol), **2a** (0.26 mmol), [Rh(C_2_H_4_)_2_Cl]_2_ (5 mol%), **L7** (15 mol%), LiO^*t*^Bu (0.6 mmol) and 3 Å MS (100 mg) in dioxane at 80 °C], C–H arylation reactions of thiocarbonylferrocene **1a** with various aryl iodides were investigated (Table 2). Notably, the absolute configuration of product **3a** (99% *ee* after recrystallization) was determined by X-ray crystallographic diffraction as *S*_p_ (see the Supplementary Table 2 for details). Both electron-donating groups (such as methyl, methoxy), halogen groups (F, Cl, and Br) and electron-withdrawing groups (cyano and trifluoromethyl) on the phenyl ring were all well tolerated, and the desired products were delivered in good yields with good to excellent enantioselectivity (**3b–h**, 58–82% yields, 89–95% *ee*). Delightedly, the reaction of 1-bromo-4-iodobenzene **2f** afforded product **3f** in 68% yield with 90% *ee*, leaving the Br atom intact. *Meta*- and *ortho*-substituted aryl iodides **1i**, **1j**, and **1k** also proceeded smoothly to give the arylated products **3i–k** in 70–80% yields with 91–97% *ee*. 2-Iodonaphthalene was found to be a suitable substrate, and **3l** was obtained in 62% yield with 97% *ee*. Notably, heteroaryl iodides such as benzofuranyl, thienyl and pyridyl iodides were found to be effective coupling partners, providing the desired products **3m–q** in 53–75% yields with 74–94% *ee*. However, 30 mol% of **L7** was required to obtain reasonable yields for thienyl and pyridyl iodides. This is likely due to their relatively strong coordination with rhodium. This asymmetric C–H arylation reaction is also compatible with other thiocarbonylferrocenes such as **1r** and **1s**, leading to the formation of **3r** and **3s** in 65–76% yields with 99% *ee*.

**Table 2:**
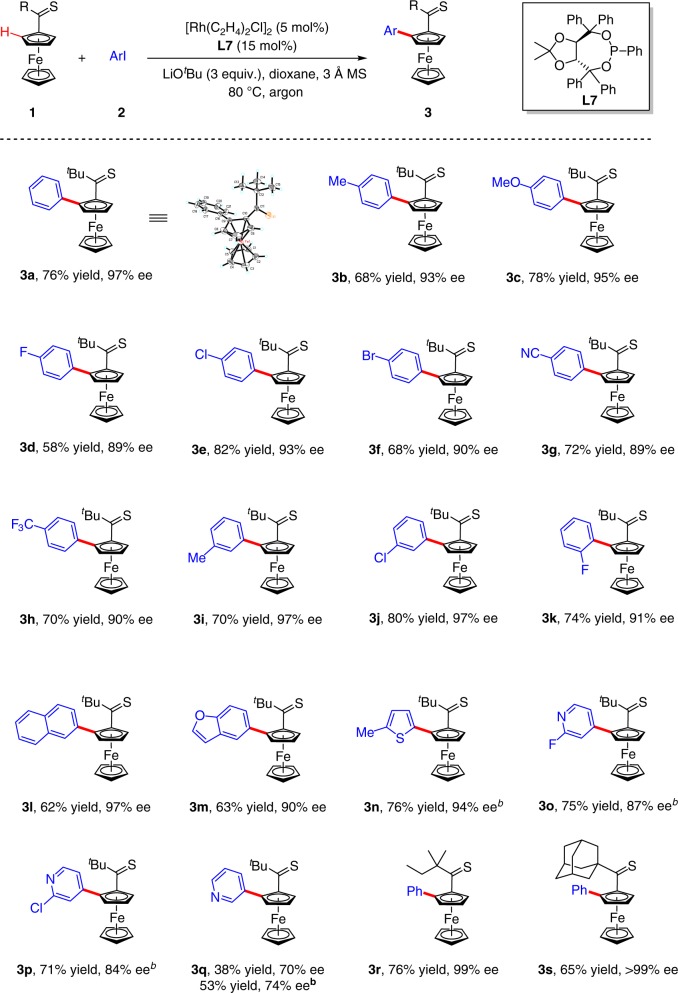
Scope of asymmetric C–H arylation^[a,c,d]^

[a] General conditions: **1** (0.2 mmol), **2** (0.26 mmol), [Rh(C_2_H_4_)_2_Cl]_2_ (5 mol%), **L7** (0.03 mmol), LiO^*t*^Bu (0.6 mmol) and 3 Å MS (100 mg) in dioxane (1.5 mL) at 80 °C, 12 h. [b] **L7** (0.06 mmol). [c] Yield of isolated product. [d] Determined by HPLC analysis

**Fig. 2:**
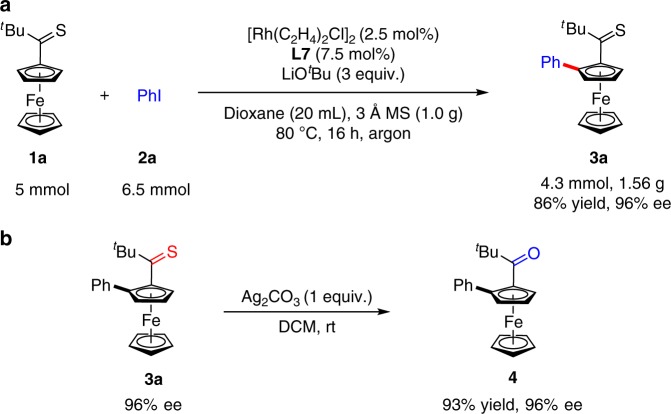
Gram-scale reaction and transformation. **a** Arylation of **1a** on a 1.56 g scale. **b** Oxidation of thioketone **3a**

To demonstrate the potential utility of such an asymmetric C–H bond arylation reaction, a gram-scale reaction was performed. Thioketone **1a** (5 mmol) could be efficiently converted into **3a** (1.56 grams) with 96% *ee* in the presence of 2.5 mol% [Rh(C_2_H_4_)_2_Cl]_2_ and 7.5 mol% **L7** (Fig. 2a). Furthermore, planar chiral ferrocenyl ketone **4** was obtained in 93% yield without erosion of enantioselectivity by the treatment with Ag_2_CO_3_ under mild conditions (Fig. 2b).

### Mechanistic studies

**Fig. 3:**
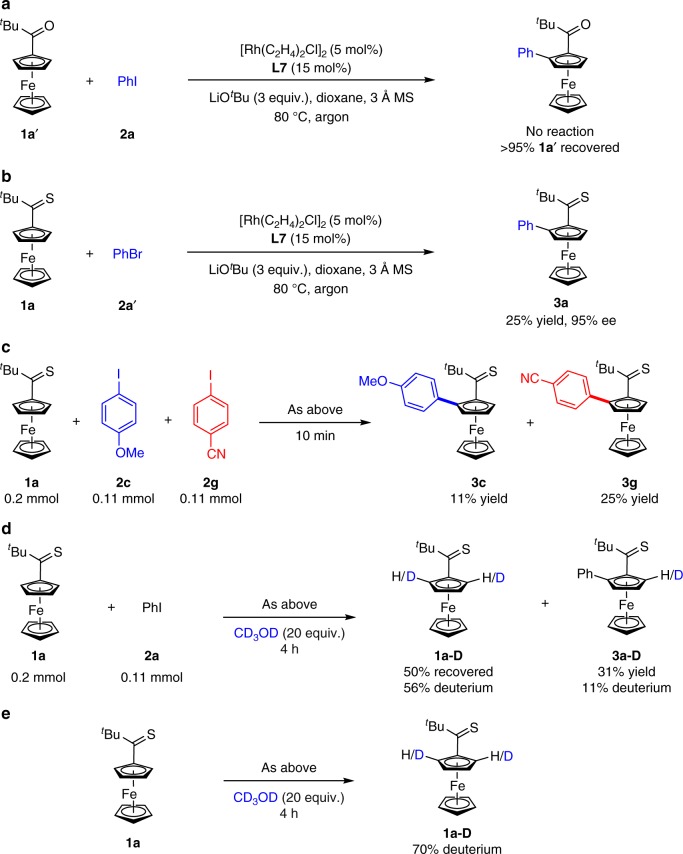
Control experiments. **a** Arylation of carbonyl compound **1a**′. **b** Bromobenzene was used as a coupling partner. **c** Competition experiment between 4-iodoanisole **2c** and 4-iodobenzonitrile **2g**. **d** H/D exchange experiment of **1a** and **2a**. **e** H/D exchange experiment of **1a**

To gain insights into this enantioselective C–H arylation reaction of ferrocenes, several control and deuteration experiments were carried out. No desired arylation product was observed when ketone substituted ferrocene **1a’** was subjected to the standard conditions, which indicated the unique role of thioketone group in the C–H bond activation process (Fig. 3a). **3a** was obtained in 25% yield with 95% ee when bromobenzene was used as a coupling partner (Fig. 3b). The competition experiment between 4-iodoanisole **2c** and 4-iodobenzonitrile **2g** revealed that electron-deficient substrate was more reactive than the electron-rich one (Fig. 3c). Significant deuteration was observed for the recovered starting material **1a** when the C–H arylation reaction was performed in the presence of 20 equiv of CD_3_OD (Fig. 3d). More importantly, we attempted this H/D exchange experiment in the absence of iodobenzene, which led to the recovered **1a** with 70% deuterium (Fig. 3e). These results indicate that the C–H cleavage of thioketone substituted ferrocene is reversible and may be not the rate-determining step.

## Discussion

In conclusion, we have developed an efficient thioketone-directed Rh(I)-catalyzed enantioselective C–H bond arylation of ferrocenes. In the presence of Rh(I) catalyst derived from [Rh(C_2_H_4_)_2_Cl]_2_ and a TADDOL-based chiral phosphonite ligand, various aryl iodides reacted with ferrocenyl thioketones smoothly in good yields. The reactions displayed a broad substrate scope, and the planar chiral ferrocenes were obtained in excellent enantioselectivity. Heteroaryl iodides also work well in this catalytic system, affording heteroaryl substituted ferrocenes, which are difficult to be obtained by the previously known methods. Further mechanistic studies and applications of the products are ongoing in our laboratory.

## Methods

### Representative procedure

3 Å MS (100 mg) was added to a dry Schlenk tube (25 mL). The flask was evacuated and backfilled with argon for 3 times. Then, LiO^*t*^Bu (0.6 mmol, 48.1 mg), **L7** (0.03 mmol, 17.2 mg), [Rh(C_2_H_4_)_2_Cl]_2_ (0.01 mmol, 3.9 mg), and **1** (0.2 mmol) were added to the Schlenk tube. The flask was evacuated and backfilled with argon for 3 times again, and followed by addition of dioxane (1.5 mL). Aryl iodide **2** (0.26 mmol, 1.3 equiv.) was added at last. The mixture was stirred at 80 °C. After the reaction was complete (monitored by TLC), the mixture was cooled to room temperature. The mixture was diluted with petroleum ether (~10 mL). Then, silica gel was added and the solvent was evaporated under reduced pressure. The product was isolated by sicila gel column chromatography (petroleum ether or petroleum ether/ethyl acetate = 20/1).

## Supplementary information

Supplementary Information

## Data Availability

The X-ray crystallographic coordinates for product **3a** have been deposited at the Cambridge Crystallographic Data Centre (CCDC) with the accession code 1894751. These data can be obtained free of charge from The Cambridge Crystallographic Data Centre via www.ccdc.cam.ac.uk/data_request/cif. The authors declare that all other data supporting the findings of this work, including experimental procedures and compound characterization data, are available within the article and its Supplementary Information files.
