# Supplementary material for: Thioketone-directed rhodium(I) catalyzed enantioselective C-H bond arylation of ferrocenes
Source: Nat Commun. 2019 Sep 13;10:4168. doi: 10.1038/s41467-019-12181-x (PMC6744407; doi:10.1038/s41467-019-12181-x)
Supplement: Supplementary file 1 — Supplementary Information [file 41467_2019_12181_MOESM1_ESM.pdf]

Supplementary Information

Thioketone-Directed Rhodium(I) Catalyzed Enantioselective C-H Bond Arylation of Ferrocenes

Cai et al.

Supplementary Notes

General Methods. Unless otherwise noted, all reactions were carried out in flame-dried glassware under a dry argon atmosphere. All solvents were purified and dried according to standard methods prior to use. All aryl iodides were obtained from the commercial sources and used directly without further purification. Rhodium precursors and LiO^{t}Bu were obtained from Strem and J&K and used directly without further purification. Compounds **1a**, **1r** and **1s** were prepared according to the reported procedure.[1] Monoprotected amino acids, ligands **L1-2** and **L16** were obtained from the commercial sources and used directly without further purification. Chiral phosphoric acids,[2] ligands **L3-4**,[3] **L14**,[4] **L5-13**[5] and **L15**[6] were prepared according to the reported procedure. ^{1}H and ^{13}C NMR spectra were recorded on a Bruker instrument (400 MHz and 100 MHz, respectively) or an Agilent instrument (400, 600 MHz and 100, 150 MHz, respectively) and internally referenced to tetramethylsilane signal or residual protic solvent signals. ^{19}F NMR spectra were recorded on a Bruker or Agilent instrument (376 MHz) and internally referenced to $CFCl_3$. Data for ^{1}H NMR are recorded as follows: chemical shift (δ, ppm), multiplicity (s = singlet, d = doublet, t = triplet, m = multiplet or unresolved, br = broad singlet, coupling constant (s) in Hz, integration). Data for ^{13}C NMR and ^{19}F NMR are reported in terms of chemical shift (δ, ppm).

Supplementary Table 1. Complete Optimization

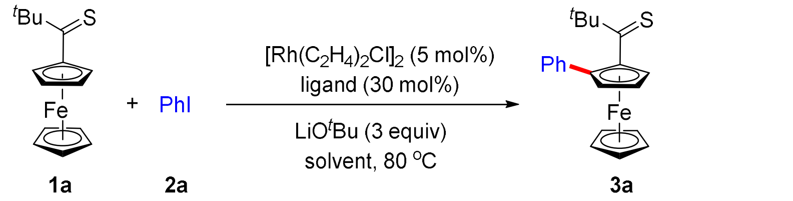

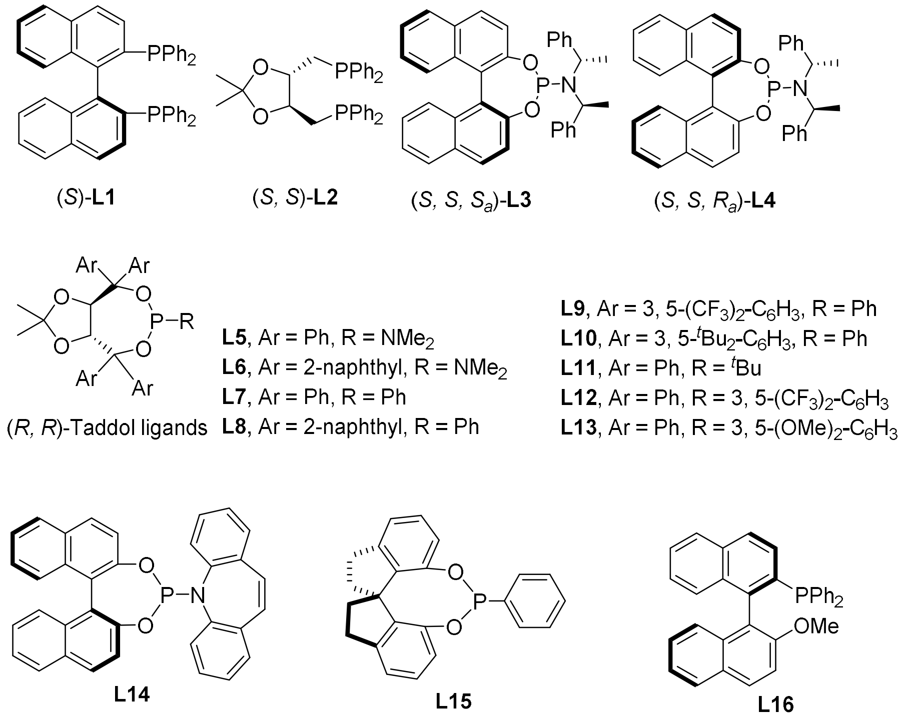

entry	ligand (equiv)	solvent	additive (100 mg)	yield (%)	ee (%)
1	**L1**	THF	--	0	--
2	**L2**	THF	--	0	--
3	**L3**	THF	--	19	88
4	**L4**	THF	--	15	-71
5	**L5**	THF	--	29	84
6	**L6**	THF	--	23	86
7	**L7**	THF	--	46	90
8	**L8**	THF	--	26	91
9	**L9**	THF	--	33	59
10	**L10**	THF	--	44	12
11	**L11**	THF	--	trace	--
12	**L12**	THF	--	45	90
13	**L13**	THF	--	46	90
14	**L14**	THF	--	trace	--
15	**L15**	THF	--	trace	--
16	**L16**	THF	--	15	-14
17	**L7**	THF	3Å MS	59	92
18[b]	**L7**	THF	3Å MS	78	94
19[b]	**L7**	THF	4Å MS	66	89
20[b]	**L7**	THF	5Å MS	58	88
21[b]	**L7**	dioxane	3Å MS	76	97
22[b]	**L7**	toluene	3Å MS	44	98

23[b]	**L7**	DCE	3Å MS	<10	--
24[b]	**L7**	DMF	3Å MS	trace	--
25[b,c]	**L7**	dioxane	3Å MS	75	97
**26[b,d]**	**L7**	**dioxane**	**3Å MS**	**76**	**97**
27[b,e]	**L7**	dioxane	3Å MS	67	94

[a]General conditions: **1a** (0.2 mmol), **2a** (0.22 mmol), $[Rh(C_2H_4)_2Cl]_2$ (5 mol%), ligand (0.06 mmol), LiO^{t}Bu (0.6 mmol) in solvent (1.5 mL) at 80 °C. [b]**2a** (0.26 mmol). [c]**L7** (0.04 mmol). [d]**L7** (0.03 mmol). [e]Yield of isolated product. [f]Determined by HPLC analysis.

General procedure for Rh(I) catalyzed enantioselective C-H bond arylations (3a-s)

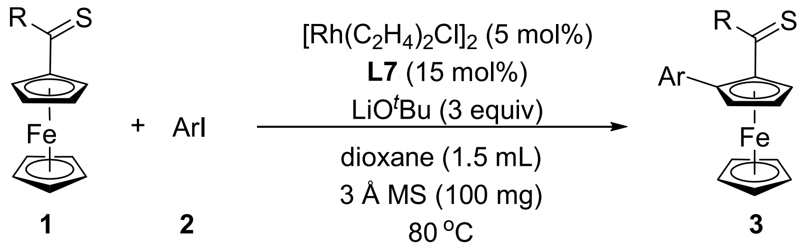

3Å MS (100 mg) was added to a 25 mL dry Schlenk tube. The flask was evacuated and backfilled with argon for 3 times. Then, LiO^{t}Bu (48.1 mg, 0.6 mmol), **L7** (17.2 mg, 0.03 mmol), $[Rh(C_2H_4)_2Cl]_2$ (3.9 mg, 0.01 mmol) and **1** (0.2 mmol) were added to the Schlenk tube, the flask was evacuated and backfilled with argon for 3 times again, and followed by addition of dioxane (1.5 mL). Then aryl iodide **2** (0.26 mmol, 1.3 equiv.) was added. The mixture was stirred at 80 ℃. After the reaction was complete (monitored by TLC), the mixture was cooled to room temperature. The mixture was diluted with petroleum ether (~10 mL), silica gel was added and the solvent was evaporated under reduced pressure. The product was isolated by silica gel column chromatography (petroleum ether or petroleum ether / ethyl acetate = 20/1).

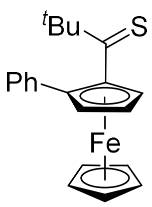

3a.[1] Purple solid, (55.1 mg, 76% yield, 97% *ee*). Analytical data for **3a**: 60-61 °C. $[\alpha]_D^{29}$ = +699.5 (c = 0.01 $CHCl_3$, 97% *ee*). ^1H NMR (400 MHz, $CDCl_3$) δ 7.49 (d, J = 8.4 Hz, 2H), 7.28-7.20 (m, 3H), 4.58 (br s, 1H), 4.54 (br s, 1H), 4.36 (br s, 1H), 4.23 (s, 5H), 1.13 (s, 9H). The enantiomeric excess was determined by Daicel Chiralcel OD-H (25 cm), Hexanes / IPA = 98 / 2, 0.50 mL/min, λ = 254 nm, t (minor) = 7.63 min, t (major) = 8.26 min.

Note: The racemization of **3a** was observed from 93% *ee* to 78% *ee* after its solution in ^{i}PrOH was heated at 75 °C for 2 hrs.

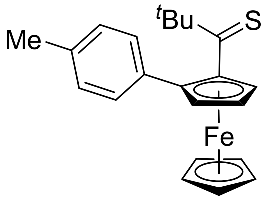

3b.[1] Purple solid, (51.1 mg, 68% yield, 93% *ee*). Analytical data for **3b**: mp: 85-86 °C. $[\alpha]_D^{29}$ = +983.0 (c = 0.01 $CHCl_3$, 93% *ee*). ^1H NMR (400 MHz, $CDCl_3$) δ 7.34 (d, J = 8.0 Hz, 2H), 7.04 (d, J = 8.0 Hz, 2H), 4.53 (dd, J = 2.4, 1.6 Hz, 1H), 4.40 (dd, J = 2.4, 1.6 Hz, 1H), 4.31 (t, J = 2.4 Hz, 1H), 4.19 (s, 5H), 2.32 (s, 3H), 1.10 (s, 9H). The enantiomeric excess was determined by Daicel Chiralcel OD-3 (15 cm), CO_2 / MeOH = 90 / 10, 1.0 mL/min, λ = 214 nm, t (minor) = 3.40 min, t (major) = 3.65 min.

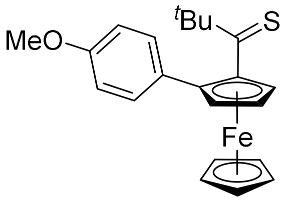

3c.[1] Purple oil, (61.1 mg, 78% yield, 95% *ee*). Analytical data for **3c**: $[\alpha]_D^{29}$ = +493.1 (c = 0.01 $CHCl_3$, 95% *ee*). ^1H NMR (400 MHz, $CDCl_3$) δ 7.34 (d, J = 8.8 Hz, 2H), 6.79 (d, J = 8.8 Hz, 2H), 4.51 (dd, J = 2.4, 1.6 Hz, 1H), 4.39 (dd, J = 2.4, 1.6 Hz, 1H), 4.30 (t, J = 2.4 Hz, 1H), 4.20 (s, 5H), 3.81 (s, 3H), 1.10 (s, 9H). The enantiomeric excess was determined by Daicel Chiralcel OD-H (25 cm), Hexanes / IPA = 98 / 2, 0.50 mL/min, λ = 254 nm, t (minor) = 8.45 min, t (major) = 9.82 min.

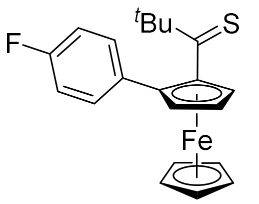

3d.[1] Purple solid, (44.2 mg, 58% yield, 89% *ee*). Analytical data for **3d**: 67-68 ^{o}C. $[\alpha]_D^{29}$ = +711.7 (c = 0.01 $CHCl_3$, 89% *ee*). ^{1}H NMR (400 MHz, $CDCl_3$) δ 7.44-7.40 (m, 2H), 6.95-6.91 (m, 2H), 4.51 (dd, *J* = 2.4, 1.6 Hz, 1H), 4.43 (dd, *J* = 2.4, 1.6 Hz, 1H), 4.33 (t, *J* = 2.4 Hz, 1H), 4.20 (s, 5H), 1.10 (s, 9H). The enantiomeric excess was determined by Daicel Chiralcel OD-H (25 cm), CO_2 / IPA = 95 / 5, 0.70 mL/min, λ = 254 nm, t (minor) = 3.67 min, t (major) = 4.01 min.

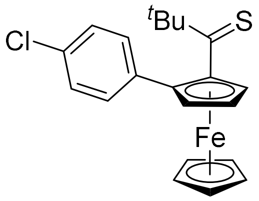

3e.[1] Purple solid, (64.9 mg, 82% yield, 93% *ee*). Analytical data for **3e**: 76-77 ^{o}C. $[\alpha]_D^{29}$ = +792.0 (c = 0.01 $CHCl_3$, 93% *ee*). ^{1}H NMR (400 MHz, $CDCl_3$) δ 7.39 (d, *J* = 8.4 Hz, 2H), 7.20 (d, *J* = 8.4 Hz, 2H), 4.52 (dd, *J* = 2.4, 1.6 Hz, 1H), 4.45 (dd, *J* = 2.4, 1.6 Hz, 1H), 4.35 (t, *J* = 2.4 Hz, 1H), 4.19 (s, 5H), 1.11 (s, 9H). The enantiomeric excess was determined by Daicel Chiralcel OD-H (25 cm), Hexanes / IPA = 98 / 2, 0.50 mL/min, λ = 254 nm, t (minor) = 7.77 min, t (major) = 8.37 min.

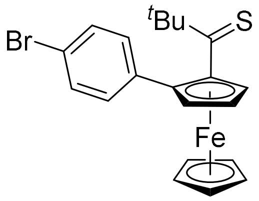

3f.[1] Purple oil, (60.0 mg, 68% yield, 90% *ee*). Analytical data for **3f**: $[\alpha]_D^{29}$ = +669.2 (c = 0.01 $CHCl_3$, 90% *ee*). ^{1}H NMR (400 MHz, $CDCl_3$) δ 7.37-7.31 (m, 4H), 4.52 (dd, *J* = 2.4, 1.6 Hz, 1H), 4.45 (dd, *J* = 2.4, 1.2 Hz, 1H), 4.35 (t, *J* = 2.4 Hz, 1H), 4.19 (s, 5H), 1.11 (s, 9H). The enantiomeric excess was determined by Daicel Chiralcel OD-H (25 cm), Hexanes / IPA = 98 / 2, 0.50 mL/min, λ = 254 nm, t (minor) = 7.70 min, t (major) = 8.41 min.

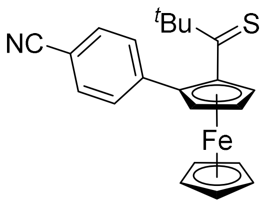

3g.[1] Purple oil, (55.7 mg, 72% yield, 89% *ee*). Analytical data for **3g**: $[\alpha]_D^{29}$ = +135.9 (c = 0.01 $CHCl_3$, 89% *ee*). ^1H NMR (400 MHz, $CDCl_3$) δ 7.54-7.49 (m, 4H), 4.58 (dd, *J* = 2.4, 1.6 Hz, 1H), 4.54 (dd, *J* = 2.4, 1.6 Hz, 1H), 4.44 (t, *J* = 2.4 Hz, 1H), 4.18 (s, 5H), 1.13 (s, 9H). The enantiomeric excess was determined by Daicel Chiralcel OD-H (25 cm), Hexanes / IPA = 98 / 2, 1.0 mL/min, λ = 254 nm, t (minor) = 6.50 min, t (major) = 6.97 min.

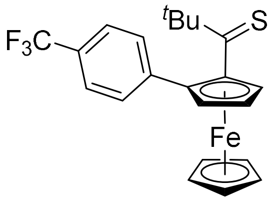

3h. Purple oil, (60.3 mg, 70% yield, 90% *ee*). Analytical data for **3h**: $[\alpha]_D^{29}$ = +566.6 (c = 0.01 $CHCl_3$, 90% *ee*). ^1H NMR (400 MHz, $CDCl_3$) δ 7.55 (d, *J* = 8.4 Hz, 2H), 7.48 (d, *J* = 8.8 Hz, 2H), 4.58 (dd, *J* = 2.4, 1.6 Hz, 1H), 4.50 (dd, *J* = 2.4, 1.6 Hz, 1H), 4.40 (t, *J* = 2.4 Hz, 1H), 4.20 (s, 5H), 1.13 (s, 9H). ^{19}F NMR (376 MHz, $CDCl_3$) δ -62.4 ^{13}C NMR (100 MHz, $CDCl_3$) δ 264.7, 143.4, 129.1, 128.2 (d, *J* = 32.1 Hz), 124.8 (q, *J* = 3.8 Hz), 124.3 (q, *J* = 270.1 Hz), 102.3, 87.3, 72.4, 71.2, 68.2, 67.0, 53.8, 31.5. IR (neat): ν_{max} (cm^{-1}) = 2968, 2920, 1614, 1413, 1321, 1119, 843, 814; HRMS (ESI) calcd for $C_{22}H_{21}F_3[^{56}Fe]S$ $[M]^+$: 430.0660. Found: 430.0663. The enantiomeric excess was determined by Daicel Chiralcel OD-H (25 cm), Hexanes / IPA = 98 / 2, 0.50 mL/min, λ = 254 nm, t (minor) = 7.49 min, t (major) = 8.01 min.

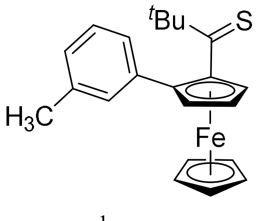

3i.[1] Purple solid, (52.6 mg, 70% yield, 97% *ee*). Analytical data for **3i**: 59-60 °C. $[\alpha]_D^{29}$ = +586.1 (c = 0.01 $CHCl_3$, 97% *ee*). ^1H NMR (400 MHz, $CDCl_3$) δ 7.28 (d, *J* = 8.4 Hz, 1H), 7.25 (s, 1H), 7.13 (t, *J* = 7.6 Hz, 1H), 7.01 (d, *J* = 8.0 Hz, 1H), 4.55 (dd, *J* = 2.4, 1.6 Hz, 1H), 4.41 (dd, *J* = 2.4, 1.6 Hz, 1H), 4.32 (t, *J* = 2.6 Hz, 1H), 4.19 (s,

5H), 2.33 (s, 3H), 1.10 (s, 9H). The enantiomeric excess was determined by Daicel Chiralcel OD-H (25 cm), Hexanes / IPA = 98 / 2, 0.50 mL/min, λ = 254 nm, t (minor) = 7.11 min, t (major) = 7.51 min.

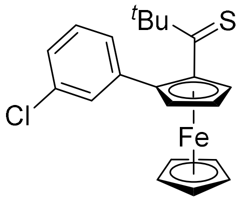

3j. Purple oil, (63.3 mg, 80% yield, 97% *ee*). Analytical data for **3j**: $[\alpha]_D^{29}$ = +606.1 (c = 0.01 $CHCl_3$, 97% *ee*). ^1H NMR (400 MHz, $CDCl_3$) δ 7.43-7.42 (m, 1H), 7.36-7.33 (m, 1H), 7.17-7.15 (m, 2H), 4.55 (dd, *J* = 2.4, 1.6 Hz, 1H), 4.46 (dd, *J* = 2.4, 1.6 Hz, 1H), 4.36 (t, *J* = 2.4 Hz, 1H), 4.20 (s, 5H), 1.12 (s, 9H). The enantiomeric excess was determined by Daicel Chiralcel OD-H (25 cm), Hexanes / IPA = 98 / 2, 0.50 mL/min, λ = 254 nm, t (minor) = 7.52 min, t (major) = 8.06 min.

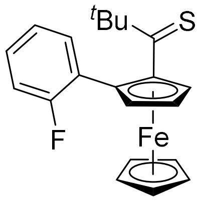

3k. Purple oil, (56.2 mg, 74% yield, 91% *ee*). Analytical data for **3l**: $[\alpha]_D^{29}$ = +1135.2 (c = 0.01 $CHCl_3$, 91% *ee*). ^1H NMR (400 MHz, $CDCl_3$) δ 7.75 (td, *J* = 7.6, 2.0 Hz, 1H), 7.24-7.18 (m, 1H), 7.12 (td, *J* = 7.6, 1.6 Hz, 1H), 6.95-6.90 (m, 1H), 4.63-4.61 (m, 2H), 4.44 (t, *J* = 2.4 Hz, 1H), 4.20 (s, 5H), 1.25 (s, 9H). ^{19}F NMR (376 MHz, $CDCl_3$) δ -113.0 (m). ^{13}C NMR (100 MHz, $CDCl_3$) δ 262.5, 159.6 (d, *J* = 245.4 Hz), 132.9 (d, *J* = 3.2 Hz) , 128.1 (d, *J* = 8.1 Hz), 126.1 (d, *J* = 13.8 Hz), 123.7 (d, *J* = 3.5 Hz), 115.3 (d, *J* = 22.4 Hz), 98.3, 86.9, 72.7, 72.0, 68.3, 67.0, 52.9, 31.4. IR (neat): ν_{max} (cm^{-1}) = 2962, 2922, 1573, 1453, 1203, 812, 756; HRMS (ESI) calcd for $C_{21}H_{21}F[^{56}Fe]S$ $[M]^+$: 380.0692. Found: 380.0691. The enantiomeric excess was determined by Daicel Chiralcel OD-H (25 cm), Hexanes / IPA = 98 / 2, 0.50 mL/min, λ = 254 nm, t (minor) = 7.87 min, t (major) = 8.54 min.

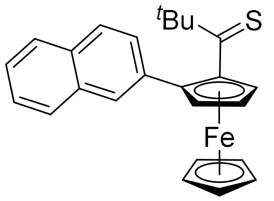

3l.[1] Purple solid, (51.1 mg, 62% yield, 97% *ee*). Analytical data for **3k**: 131-132 °C. $[\alpha]_D^{29}$ = +1069.2 (c = 0.01 $CHCl_3$, 97% *ee*). ^1H NMR (400 MHz, $CDCl_3$) δ 7.89 (s, 1H), 7.80 (t, *J* = 8.0 Hz, 2H), 7.71 (d, *J* = 8.4 Hz, 1H), 7.66 (dd, *J* = 8.4, 1.6 Hz, 1H), 7.49-7.42 (m, 2H), 4.70 (dd, *J* = 2.4, 1.6 Hz, 1H), 4.48 (dd, *J* = 2.4, 1.6 Hz, 1H), 4.39 (t, *J* = 2.4 Hz, 1H), 4.24 (s, 5H), 1.12 (s, 9H). The enantiomeric excess was determined by Daicel Chiralcel AH-3 (15 cm), CO_2 / MeOH = 90 / 10, 1.0 mL/min, λ = 214 nm, t (major) = 5.33 min, t (minor) = 6.44 min.

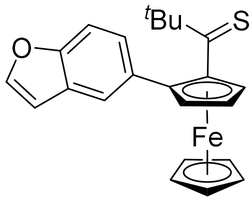

3m. Purple oil, (50.7 mg, 63% yield, 90% *ee*). Analytical data for **3m**: $[\alpha]_D^{29}$ = +853.3 (c = 0.01 $CHCl_3$, 90% *ee*). ^1H NMR (400 MHz, $CDCl_3$) δ 7.68 (s, 1H), 7.60 (d, *J* = 2.0 Hz, 1H), 7.43 (d, *J* = 8.8, 1H), 7.36 (d, *J* = 8.8 Hz, 1H), 6.73 (s, 1H), 4.58 (dd, *J* = 2.4, 1.6 Hz, 1H), 4.43 (dd, *J* = 2.4, 1.6 Hz, 1H), 4.33 (t, *J* = 2.4 Hz, 1H), 4.22 (s, 5H), 1.08 (s, 9H). ^{13}C NMR (100 MHz, $CDCl_3$) δ 266.1, 153.9, 145.3, 133.4, 127.2, 126.1, 121.3, 110.6, 106.6, 102.9, 89.0, 72.1, 71.1, 68.0, 66.2, 53.8, 31.5. IR (neat): ν_{max} (cm^{-1}) = 3075, 2921, 1462, 1445, 1253, 1222, 812, 734; HRMS (ESI) calcd for $C_{23}H_{22}[^{56}Fe]OS$ $[M]^+$: 402.0735. Found: 402.0743. The enantiomeric excess was determined by Daicel Chiralcel OD-H (25 cm), Hexanes / IPA = 98 / 2, 0.50 mL/min, λ = 254 nm, t (minor) = 8.91 min, t (major) = 9.48 min.

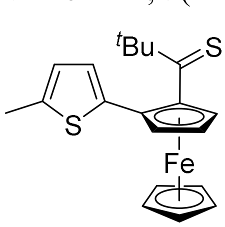

3n. Purple oil, (58.1 mg, 76% yield, 94% *ee*). Analytical data for **3n**: $[\alpha]_D^{29}$ = +230.9 (c = 0.01 $CHCl_3$, 94% *ee*). ^1H NMR (400 MHz, $CDCl_3$) δ 6.72 (d, *J* = 3.6 Hz,

1H), 6.53-6.52 (m, 1H), 4.52 (dd, J = 2.4, 1.6 Hz, 1H), 4.37 (dd, J = 2.4, 1.6 Hz, 1H), 4.29 (t, J = 2.4 Hz, 1H), 4.24 (s, 5H), 2.42 (s, 3H), 1.17 (s, 9H). ^{13}C NMR (100 MHz, $CDCl_3$) δ 263.8, 138.9, 138.6, 125.7, 124.9, 102.2, 83.0, 72.3, 68.7, 68.3, 66.0, 53.8, 31.2, 15.4. IR (neat): ν_{max} (cm^{-1}) = 2983, 2954, 1501, 1411, 1099, 1222, 818, 793; HRMS (ESI) calcd for $C_{20}H_{22}[^{56}Fe]S_2$ $[M]^+$: 382.0507. Found: 382.0507. The enantiomeric excess was determined by Daicel Chiralcel OD-H (25 cm), Hexanes / IPA = 98 / 2, 0.50 mL/min, λ = 254 nm, t (minor) = 7.56 min, t (major) = 7.97 min.

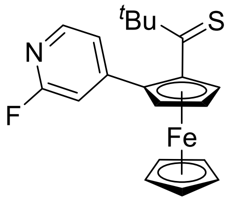

3o. Purple oil, (57.2 mg, 75% yield, 87% *ee*). Analytical data for **3o**: $[\alpha]_D^{29}$ = +703.1 (c = 0.01 $CHCl_3$, 87% *ee*). ^{1}H NMR (400 MHz, $CDCl_3$) 8.02 (d, J = 5.2 Hz, 1H), 7.22-7.19 (m, 1H), 6.94 (s, 1H), 4.62 (dd, J = 2.4, 1.6 Hz, 1H), 4.55 (dd, J = 2.4, 1.6 Hz, 1H), 4.46 (t, J = 2.4 Hz, 1H), 4.19 (s, 5H), 1.15 (s, 9H). ^{19}F NMR (376 MHz, $CDCl_3$) δ -69.0 (m). ^{13}C NMR (100 MHz, $CDCl_3$) δ 264.1, 163.9 (d, J = 235.9 Hz), 154.5 (d, J = 8.5 Hz), 146.9 (d, J = 15.6 Hz) , 121.3 (d, J = 3.8 Hz), 108.3 (d, J = 37.9 Hz), 102.5, 83.6, 72.7, 72.1, 70.6, 68.1, 67.8, 54.0, 31.5. IR (neat): ν_{max} (cm^{-1}) = 2965, 2922, 1604, 1547, 1433, 1360, 1262, 820; HRMS (ESI) calcd for $C_{20}H_{21}F[^{56}Fe]NS$ $[M+H]^+$: 382.0723. Found: 382.0715. The enantiomeric excess was determined by Daicel Chiralcel OD-H (25 cm), Hexanes / IPA = 95 / 5, 1.0 mL/min, λ = 254 nm, t (minor) = 5.44 min, t (major) = 8.35 min.

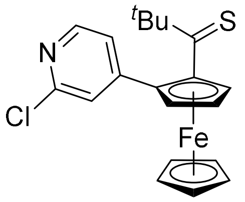

3p. Purple oil, (56.4 mg, 71% yield, 84% *ee*). Analytical data for **3p**: $[\alpha]_D^{29}$ = +926.0 (c = 0.01 $CHCl_3$, 84% *ee*). ^{1}H NMR (400 MHz, $CDCl_3$) 8.18 (d, J = 5.2 Hz, 1H), 7.34 (s, 1H), 7.25 (d, J = 5.2 Hz, 1H), 4.61 (dd, J = 2.4, 1.6 Hz, 1H), 4.56 (dd, J = 2.4, 1.6 Hz, 1H), 4.46 (t, J = 2.4 Hz, 1H), 4.19 (s, 5H), 1.16 (s, 9H). ^{13}C NMR (100 MHz, $CDCl_3$) δ 264.0, 152.2, 151.4, 149.0, 123.1, 122.3, 102.4, 83.4, 72.7, 72.1, 68.1,

67.9, 54.0, 31.5. IR (neat): ν_{max} (cm^{-1}) = 2966, 2921, 1586, 1529, 1428, 1149, 1124, 820, 758; HRMS (ESI) calcd for $C_{20}H_{21}Cl[^{56}Fe]NS$ $[M+H]^+$: 398.0427. Found: 398.0423. The enantiomeric excess was determined by Daicel Chiralcel OD-H (25 cm), Hexanes / IPA = 95 / 5, 1.0 mL/min, λ = 254 nm, t (minor) = 5.76 min, t (major) = 8.65 min.

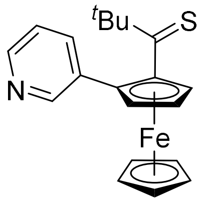

3q. Purple oil, (38.5 mg, 53% yield, 74% *ee*). Analytical data for **3q**: $[\alpha]_D^{29}$ = +616.6 (c = 0.01 $CHCl_3$, 74% *ee*). ^1H NMR (400 MHz, $CDCl_3$) 8.67 (s, 1H), 8.42 (s, 1H), 7.74 (d, *J* = 7.6 Hz, 1H), 7.16 (t, *J* = 6.8 Hz, 1H), 4.57 (br s, 1H), 4.53 (br s, 1H), 4.41 (d, *J* = 2.4 Hz, 1H), 4.19 (s, 5H), 1.15 (s, 9H). ^{13}C NMR (100 MHz, $CDCl_3$) δ 263.3, 150.0, 147.4, 136.5, 134.9, 122.6, 100.9, 86.2, 72.4, 69.8, 69.0, 67.0, 53.5, 31.3. IR (neat): ν_{max} (cm^{-1}) = 2963, 2927, 1565, 1410, 1359, 1137, 810, 708; HRMS (ESI) calcd for $C_{20}H_{22}Cl[^{56}Fe]NS$ $[M+H]^+$: 364.0817. Found: 364.0804. The enantiomeric excess was determined by Daicel Chiralcel OD-H (25 cm), Hexanes / IPA = 90 / 10, 1.0 mL/min, λ = 254 nm, t (minor) = 5.32 min, t (major) = 7.87 min.

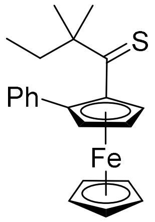

3r.[1] Purple oil, (57.2 mg, 76% yield, 99% *ee*). Analytical data for **3r**: $[\alpha]_D^{29}$ = +450.0 (c = 0.01 $CHCl_3$, 99% *ee*). ^1H NMR (400 MHz, $CDCl_3$) *δ* 7.46 (d, *J* = 8.0 Hz, 2H), 7.24-7.19 (m, 3H), 4.53 (dd, *J* = 2.4, 1.6 Hz, 1H), 4.42 (dd, *J* = 2.4, 1.6 Hz, 1H), 4.33 (t, *J* = 2.4 Hz, 1H), 4.20 (s, 5H), 1.64 (q, *J* = 7.6 Hz, 2H), 1.03 (s, 3H), 0.99 (s, 3H), 0.68 (t, *J* = 7.6 Hz, 3H). The enantiomeric excess was determined by Daicel Chiralcel OD-H (25 cm), Hexanes / IPA = 98 / 2, 0.50 mL/min, λ = 254 nm, t (minor) = 7.44 min, t (major) = 8.10 min.

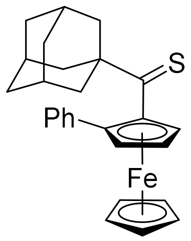

3s.[1] Purple oil, (57.2 mg, 65% yield, >99% *ee*). Analytical data for **3s**: $[\alpha]_D^{29}$ = +609.1 (c = 0.01 $CHCl_3$, >99% *ee*). ^1H NMR (400 MHz, $CDCl_3$) δ 7.45 (dd, J = 8.4, 1.6 Hz, 2H), 7.24-7.18 (m, 3H), 4.55 (dd, J = 2.4, 1.2 Hz, 1H), 4.36 (dd, J = 2.4, 1.6 Hz, 1H), 4.31 (t, J = 2.4 Hz, 1H), 4.21 (s, 5H), 1.86-1.85 (m, 3H), 1.77-1.76 (m, 1H), 1.74-1.73 (m, 2H), 1.63-1.62 (m, 2H), 1.59-1.58 (m, 2H), 1.54 (s, 2H), 1.47 (s, 2H), 1.44 (s, 1H). The enantiomeric excess was determined by Daicel Chiralcel OD-H (25 cm), Hexanes / IPA = 98 / 2, 0.50 mL/min, λ = 254 nm, t (minor) = 7.57 min, t (major) = 8.05 min.

Procedure for transformation of 3a

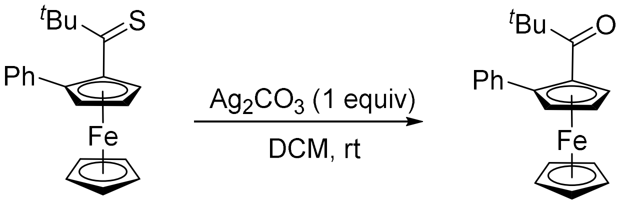

To a 25 mL flask equipped with a magnetic stir bar was added thiocarbonylferrocene (**3a**, 96% ee, 72.4 mg, 0.2 mmol), Ag_2CO_3 (55.2 mg, 0.2 mmol) and dichloromethane (2 mL). The reaction was stirred for 5 h, filtered through a pad of celite, then quenched with saturated aqueous NH_4Cl solution and extracted with EtOAc (15 mL × 3). The combined organic layers were washed with brine, dried over anhydrous Na_2SO_4 and filtrated. After the solvent was removed under reduced pressure, the residue was purified by silica gel column chromatography (ethyl acetate/petroleum ether = 1/20, v/v) to afford the desired product **4**.

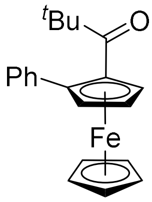

4.[1] Yellow oil, (64.3 mg, 93% yield, 96% *ee*). Analytical data for **4**: $[\alpha]_D^{29}$ = +136.6 (c = 0.1 $CHCl_3$, 96% *ee*). ^1H NMR (400 MHz, $CDCl_3$) δ 7.45 (d, *J* = 6.8 Hz, 2H), 7.30-7.26 (m, 2H), 7.25-7.21 (m, 1H), 4.55 (br s, 1H), 4.54 (br s, 1H), 4.39 (t, *J* = 2.4 Hz, 1H), 4.23 (s, 5H), 1.13 (s, 9H). The enantiomeric excess was determined by Daicel Chiralcel OD-H (25 cm), Hexanes / IPA = 98 / 2, 1.0 mL/min, λ = 254 nm, t (minor) = 4.40 min, t (major) = 4.97 min.

Supplementary Figure 1. X-Ray crystal structure of **3a**
A single crystal of **3a (CCDC 1894751)** was obtained through slow evaporation from its solution in petroleum ether.

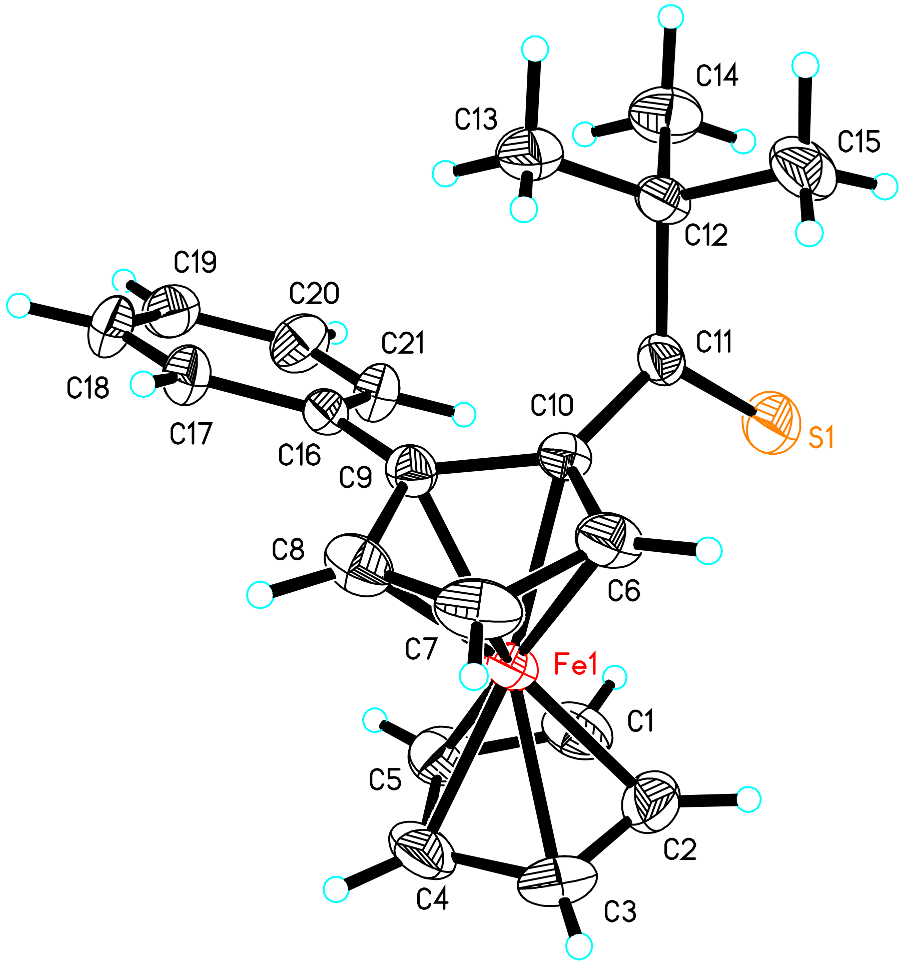

Supplementary Table 2. Crystal data and structure refinement for mo_d8v18779_0m.

Identification code	mo_d8v18779_0m	
Empirical formula	C21 H22 Fe S	
Formula weight	362.29	
Temperature	296(2) K	
Wavelength	0.71073 Å	
Crystal system	Monoclinic	
Space group	P 21	
Unit cell dimensions	a = 9.1350(4) Å	a= 90°.
	b = 9.6567(4) Å	b= 98.924(2)°.

	c = 10.3296(5) Å	g = 90°.
Volume	900.18(7) $Å^3$	
Z	2	
Density (calculated)	1.337 Mg/m^3	
Absorption coefficient	0.950 mm^{-1}	
F(000)	380	
Crystal size	0.190 x 0.160 x 0.130 mm^3	
Theta range for data collection	2.257 to 25.996°.	
Index ranges	-11<=h<=11, -11<=k<=11, -12<=l<=12	
Reflections collected	9112	
Independent reflections	3491 [R(int) = 0.0236]	
Completeness to theta = 25.242°	99.0 %	
Absorption correction	Semi-empirical from equivalents	
Max. and min. transmission	0.7456 and 0.6366	
Refinement method	Full-matrix least-squares on F^2	
Data / restraints / parameters	3491 / 1 / 212	
Goodness-of-fit on F^2	1.124	
Final R indices [I>2sigma(I)]	R1 = 0.0330, wR2 = 0.0920	
R indices (all data)	R1 = 0.0350, wR2 = 0.0939	
Absolute structure parameter	0.015(7)	
Largest diff. peak and hole	0.995 and -0.189 e.$Å^{-3}$	

Supplementary Figure 2. ^{1}H NMR spectra of **3a**

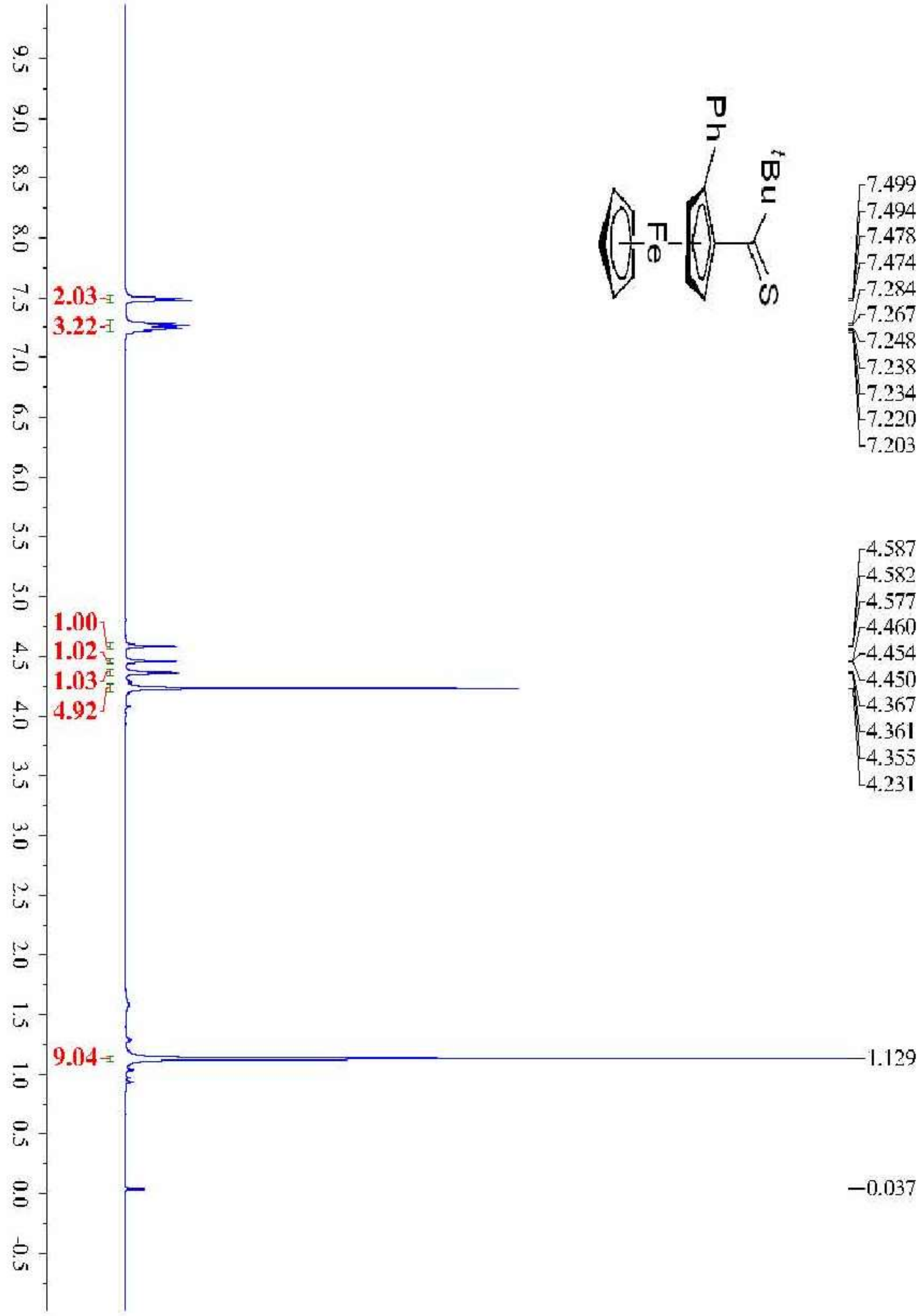

Supplementary Figure 3. HPLC analysis **3a**

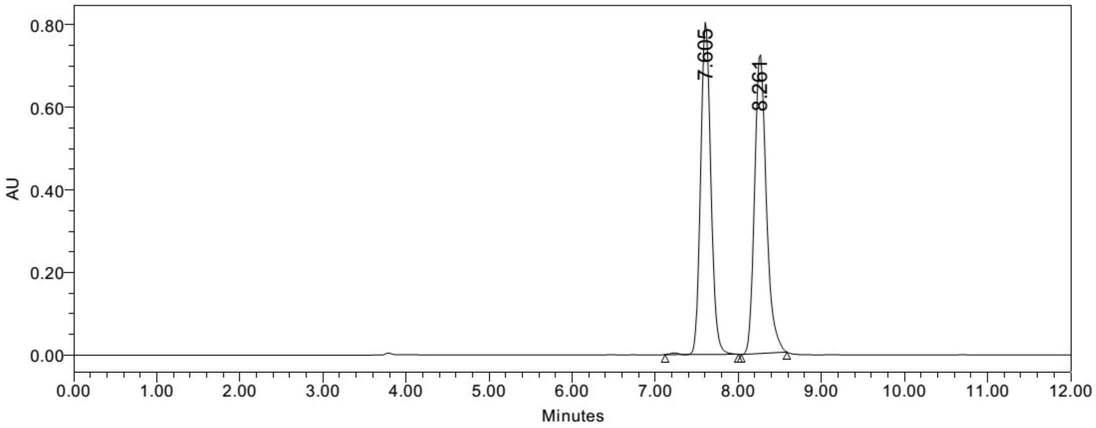

	RT	Area	% Area	Height
1	7.605	6906075	49.13	805305
2	8.261	7149368	50.87	724667

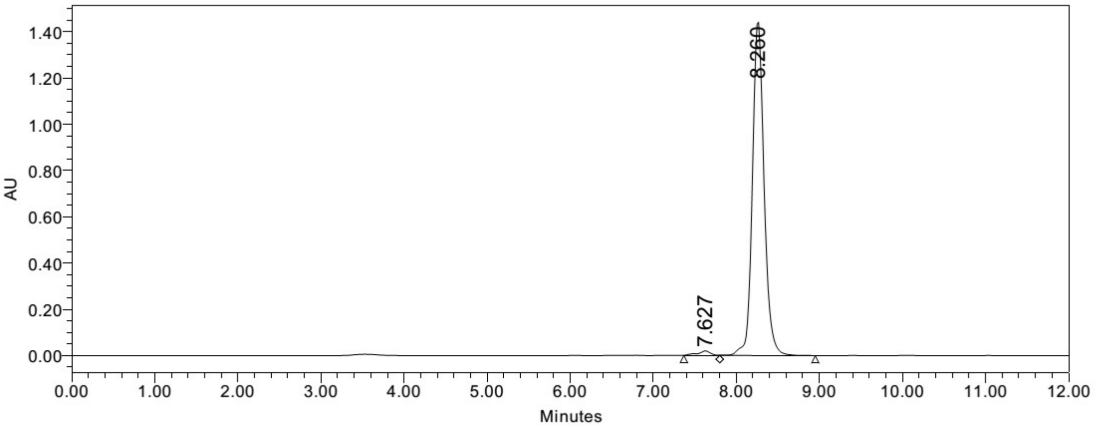

	RT	Area	% Area	Height
1	7.627	199365	1.39	18756
2	8.260	14151003	98.61	1445804

Supplementary Figure 4. ^{1}H NMR spectra of **3b**

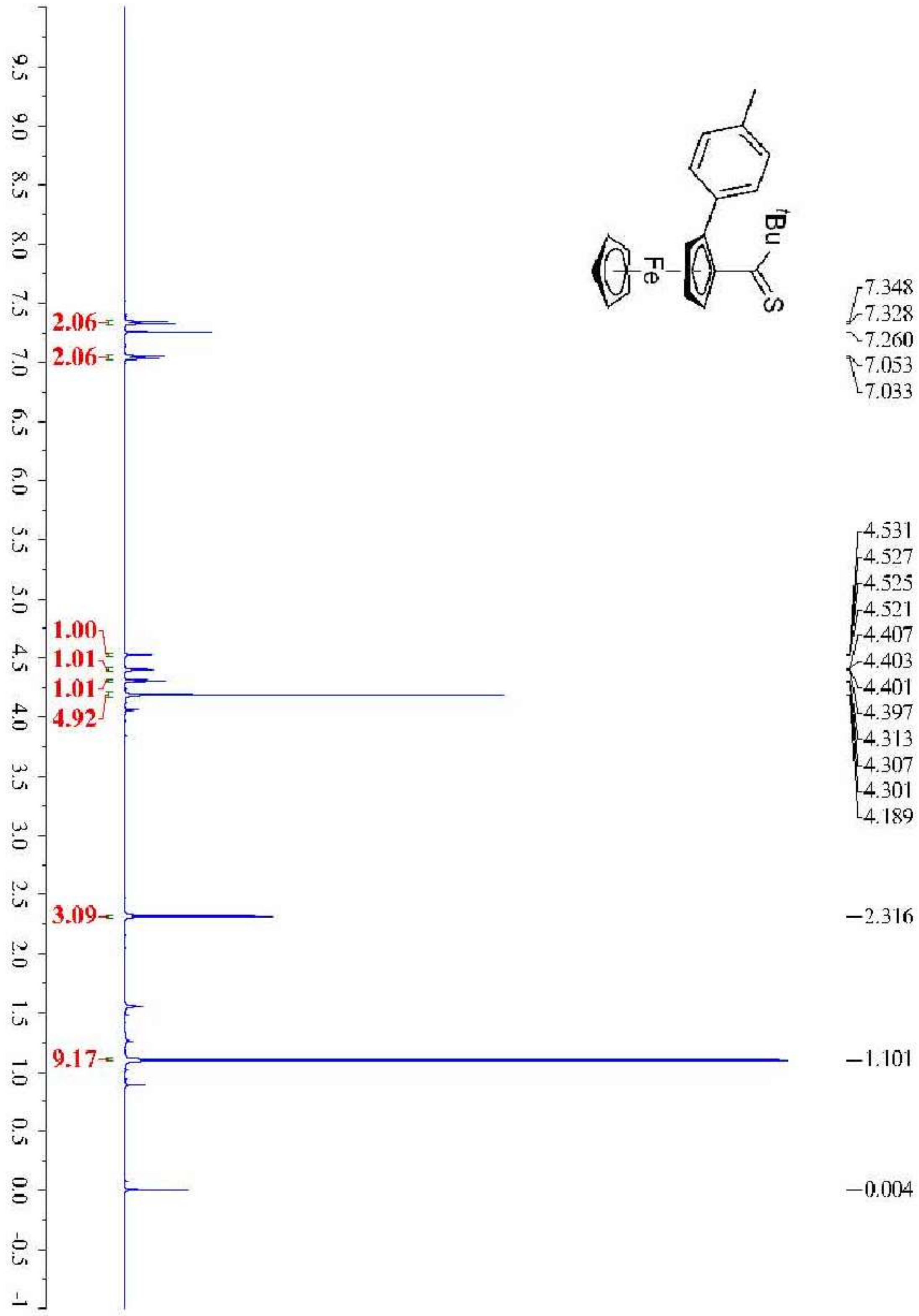

Supplementary Figure 5. HPLC analysis **3b**

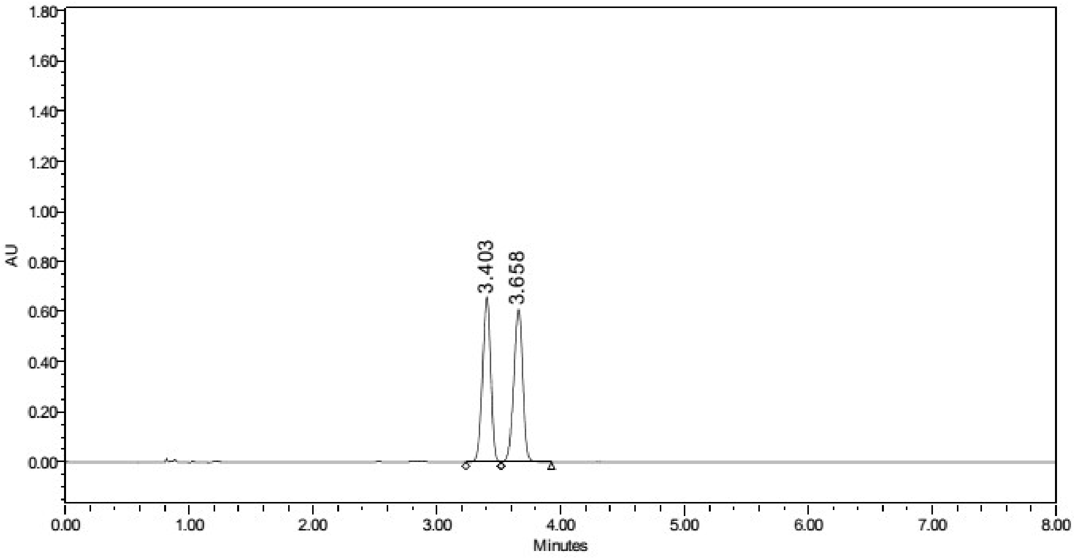

	RT	Peak Type	Height	Width (sec)	Area	% Area
1	3.403	Unknown	658871	17.050	3106404	49.92
2	3.658	Unknown	609603	24.300	3116198	50.08

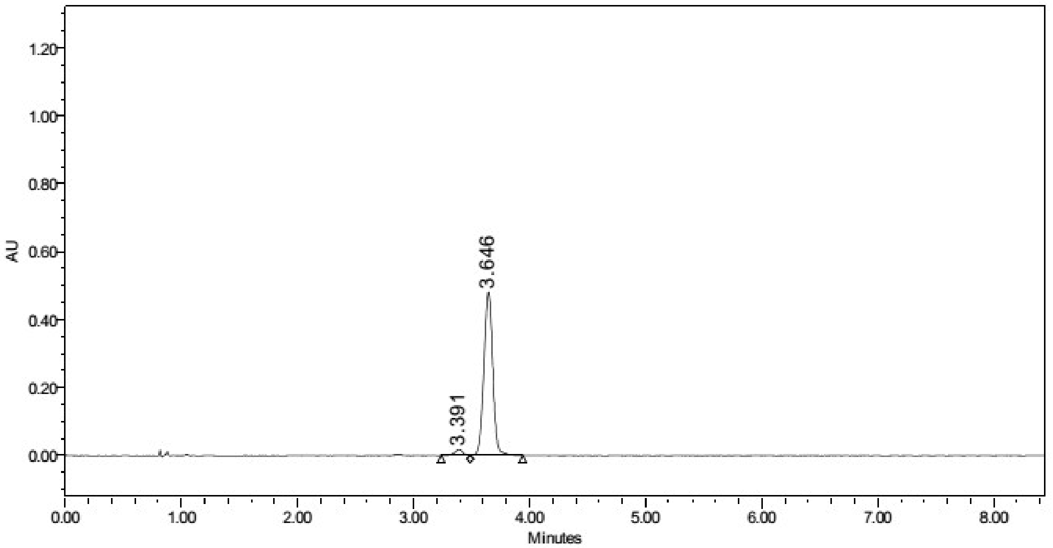

	RT	Peak Type	Height	Width (sec)	Area	% Area
1	3.391	Unknown	17676	15.000	86984	3.30
2	3.646	Unknown	480662	27.300	2548173	96.70

Supplementary Figure 6. ^{1}H NMR spectra of **3c**

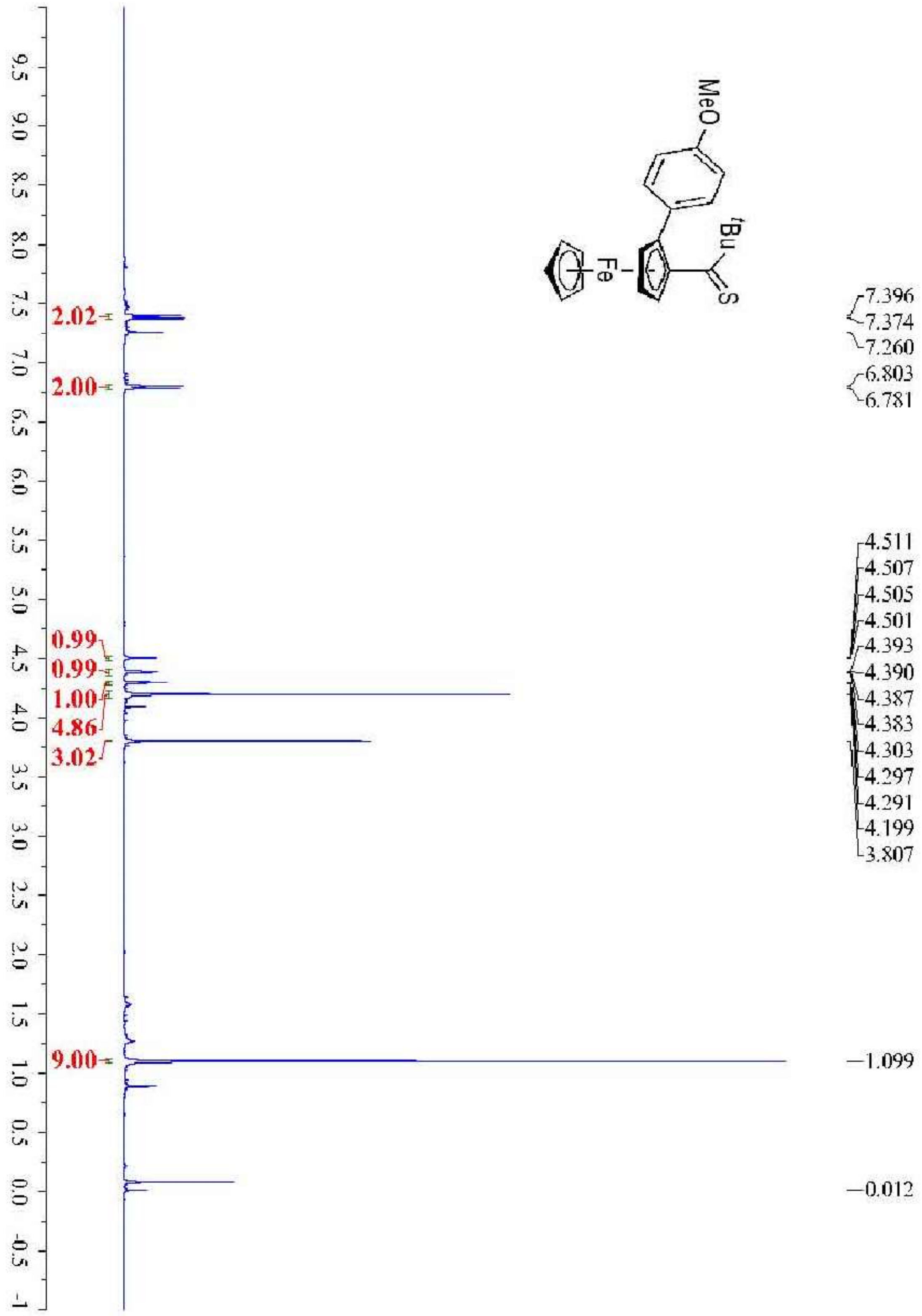

Supplementary Figure 7. HPLC analysis **3c**

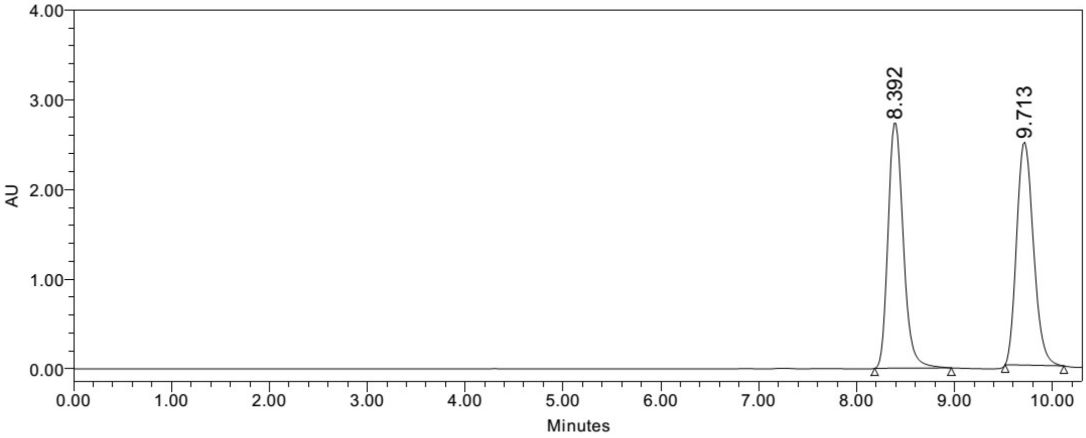

	RT	Area	% Area	Height
1	8.392	29679521	49.89	2748991
2	9.713	29806664	50.11	2489440

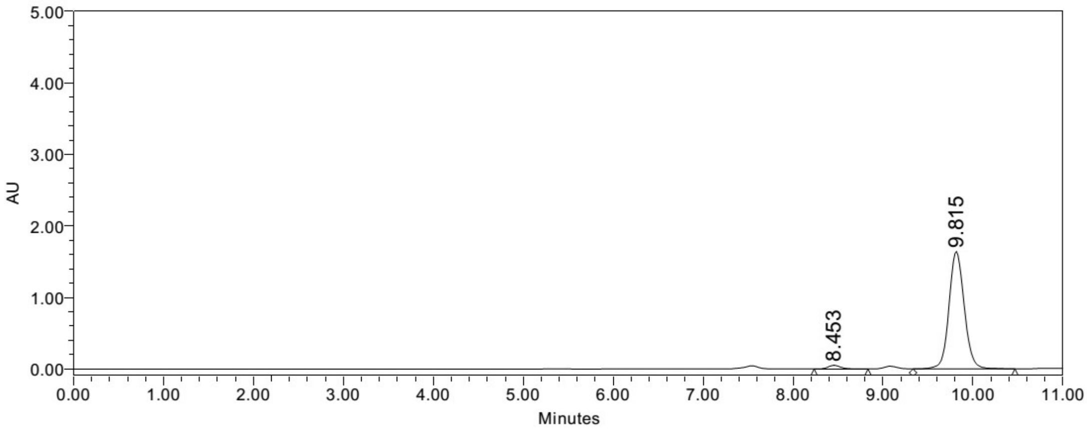

	RT	Area	% Area	Height
1	8.453	503614	2.53	50922
2	9.815	19379034	97.47	1643348

Supplementary Figure 8. ^{1}H NMR and ^{19}F NMR spectra of **3d**

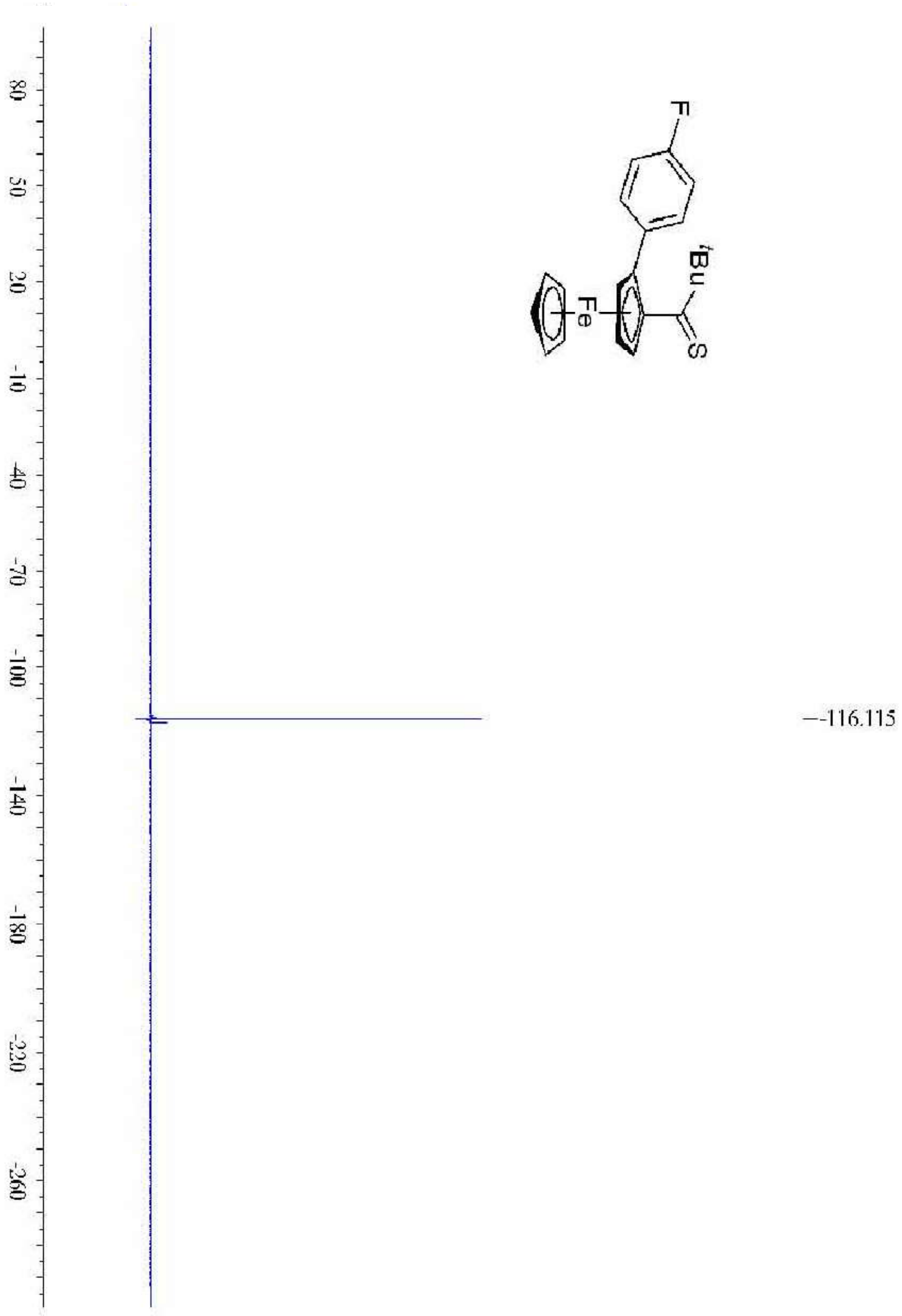

Supplementary Figure 9. HPLC analysis **3d**

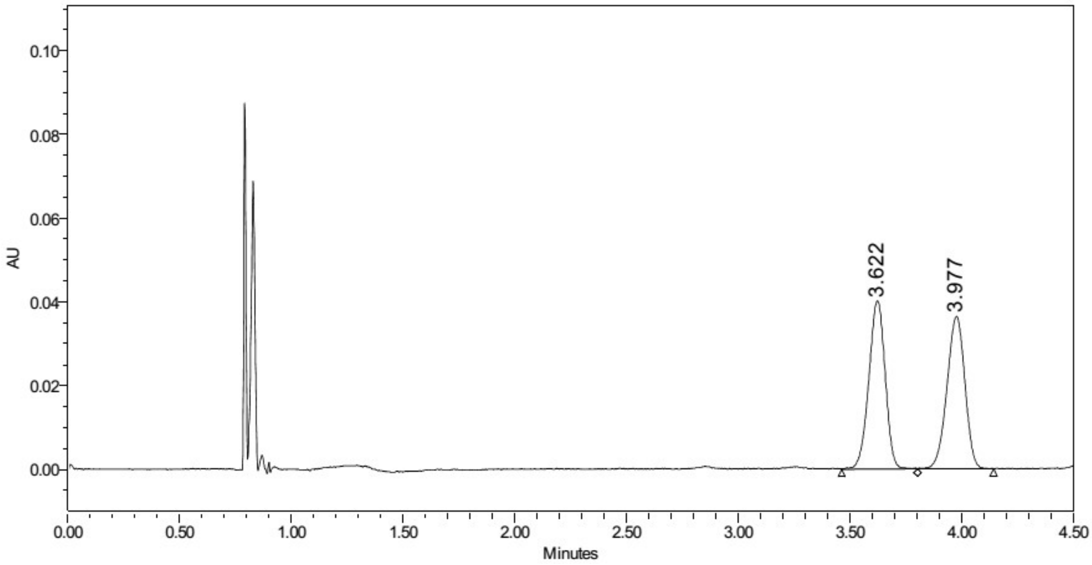

	RT	Peak Type	Height	Width (sec)	Area	% Area
1	3.622	Unknown	40125	20.350	204407	50.06
2	3.977	Unknown	36464	20.450	203941	49.94

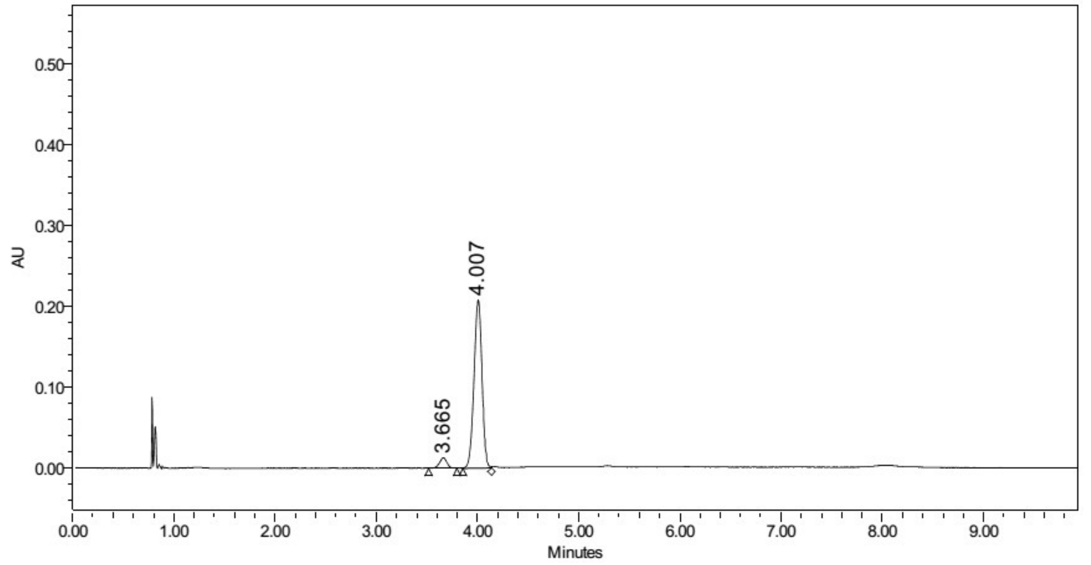

	RT	Peak Type	Height	Width (sec)	Area	% Area
1	3.665	Unknown	12705	16.900	64401	5.34
2	4.007	Unknown	208379	16.950	1142461	94.66

Supplementary Figure 10. ^{1}H NMR of **3e**

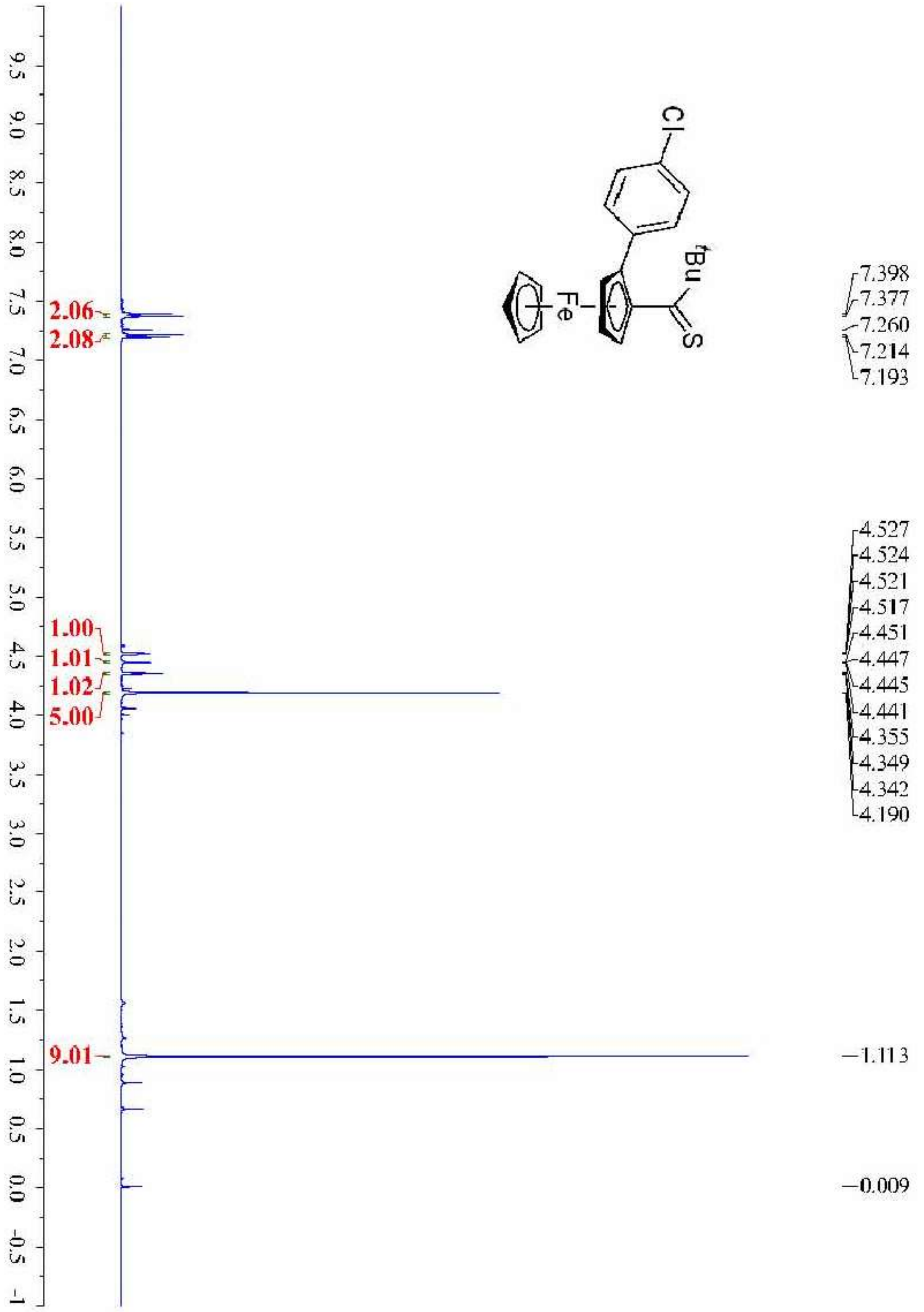

Supplementary Figure 11. HPLC analysis **3e**

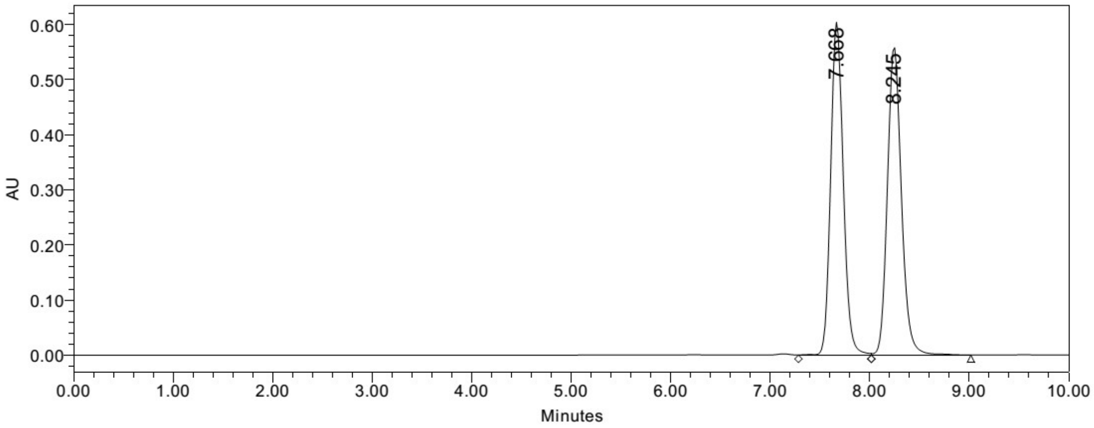

	RT	Area	% Area	Height
1	7.668	5282541	49.09	603256
2	8.245	5479058	50.91	559130

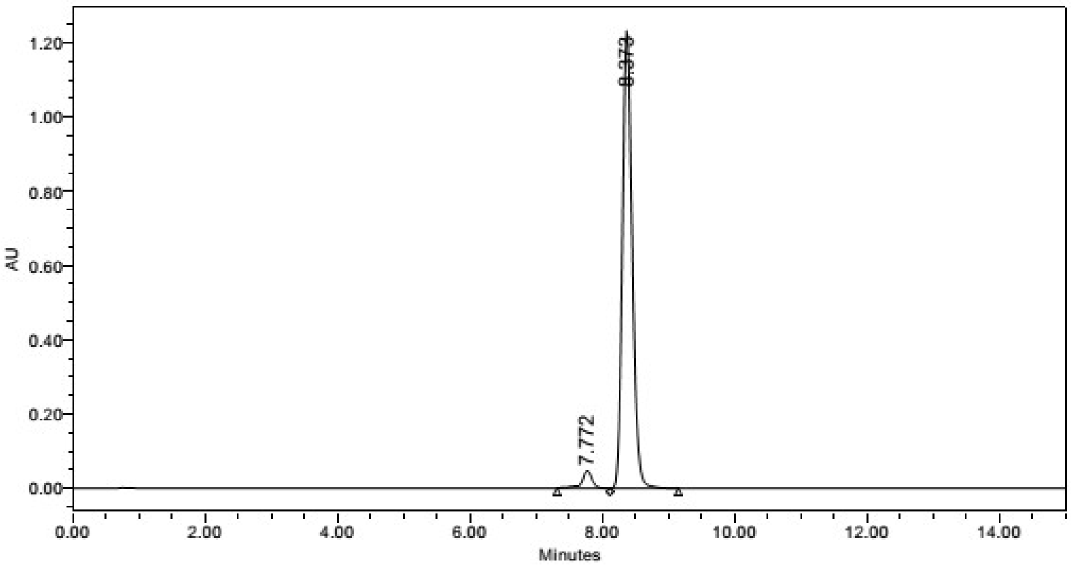

	RT	Area	% Area	Height
1	7.772	488985	3.72	47013
2	8.373	12672428	96.28	1235460

Supplementary Figure 12. ^{1}H NMR spectra of **3f**

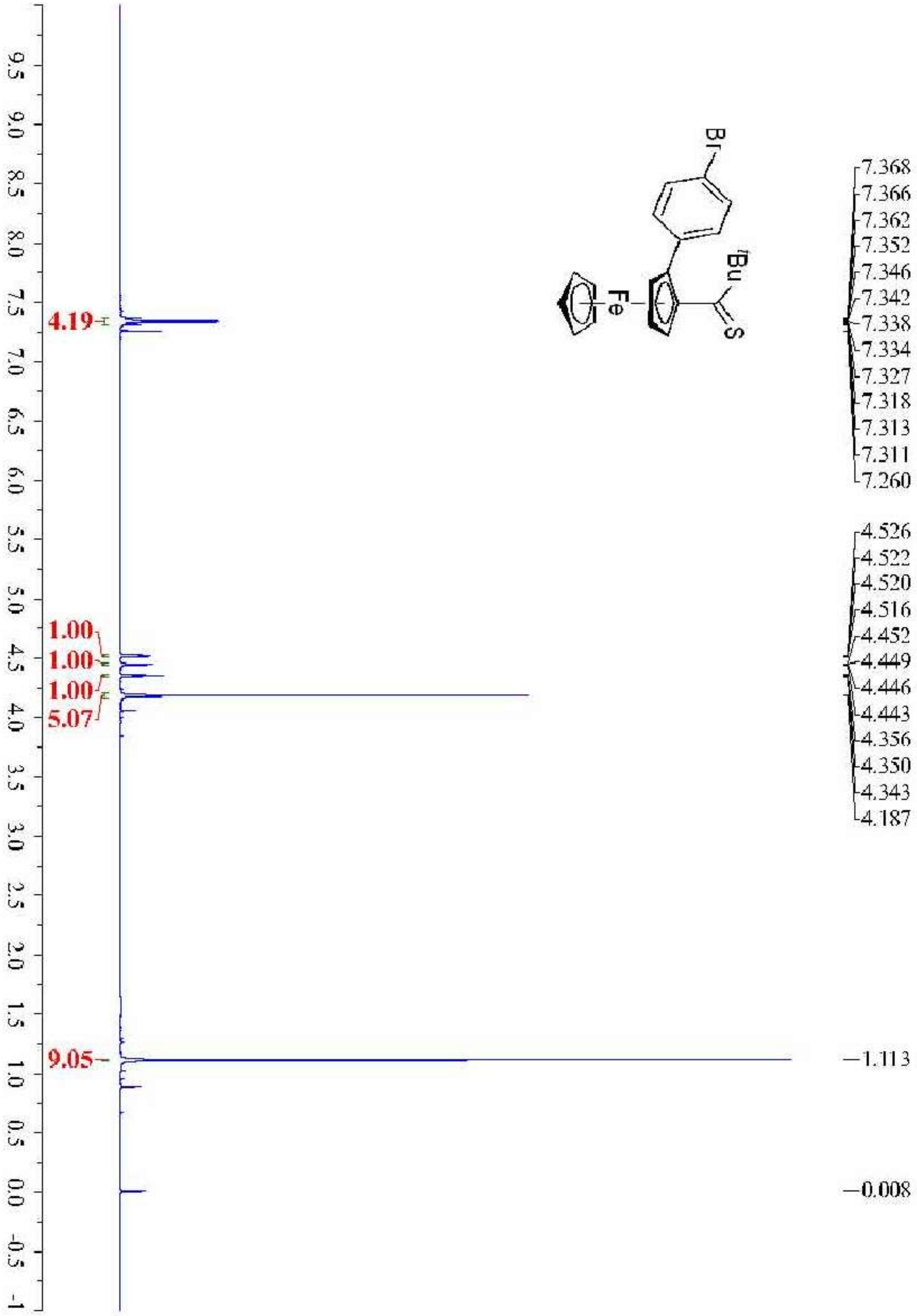

Supplementary Figure 13. HPLC analysis **3f**

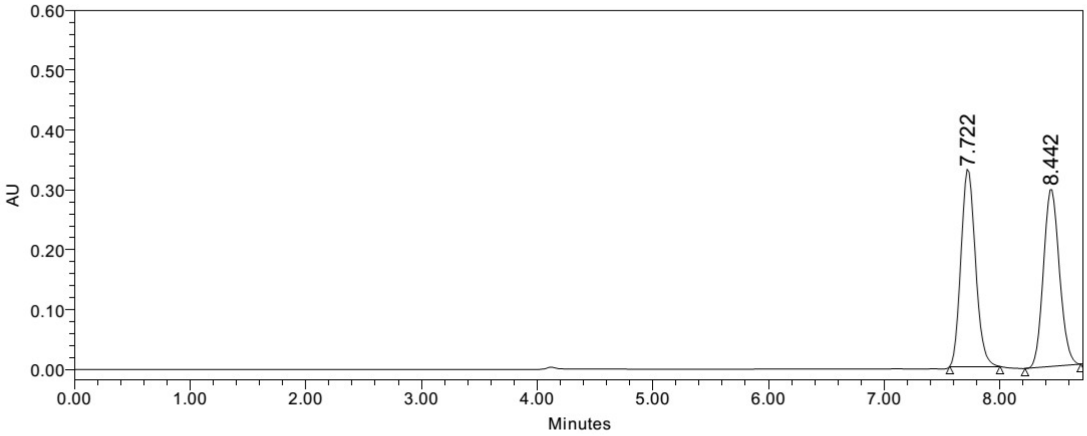

	RT	Area	% Area	Height
1	7.722	2872956	50.17	330536
2	8.442	2853946	49.83	297514

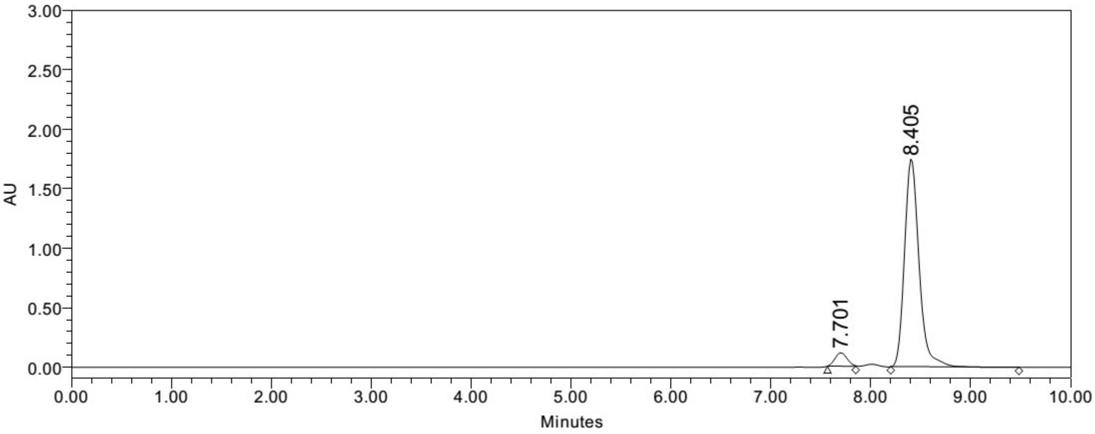

	RT	Area	% Area	Height
1	7.701	937653	5.04	112167
2	8.405	17654509	94.96	1748373

Supplementary Figure 14. ^{1}H NMR spectra of **3g**

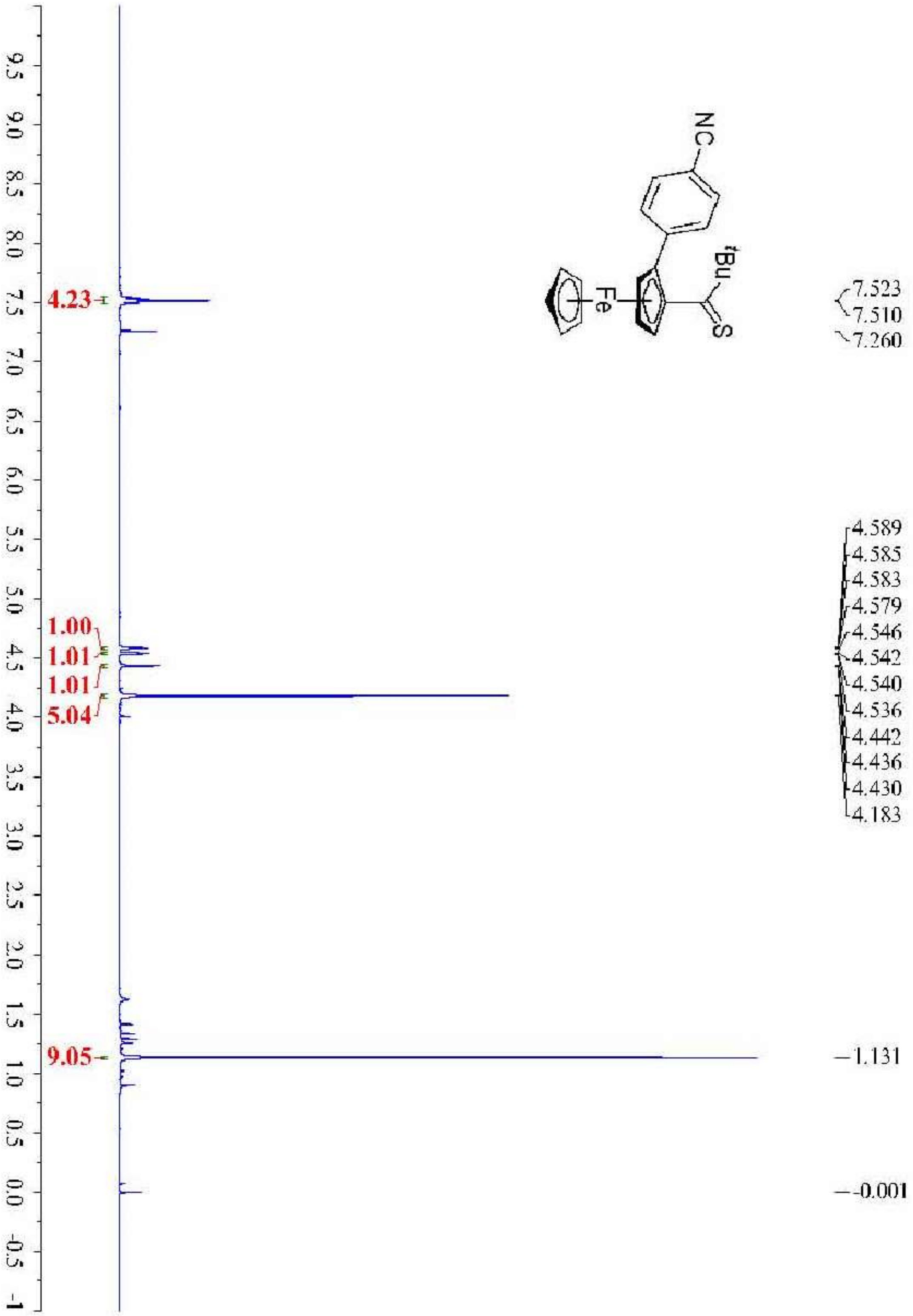

Supplementary Figure 15. HPLC analysis **3g**

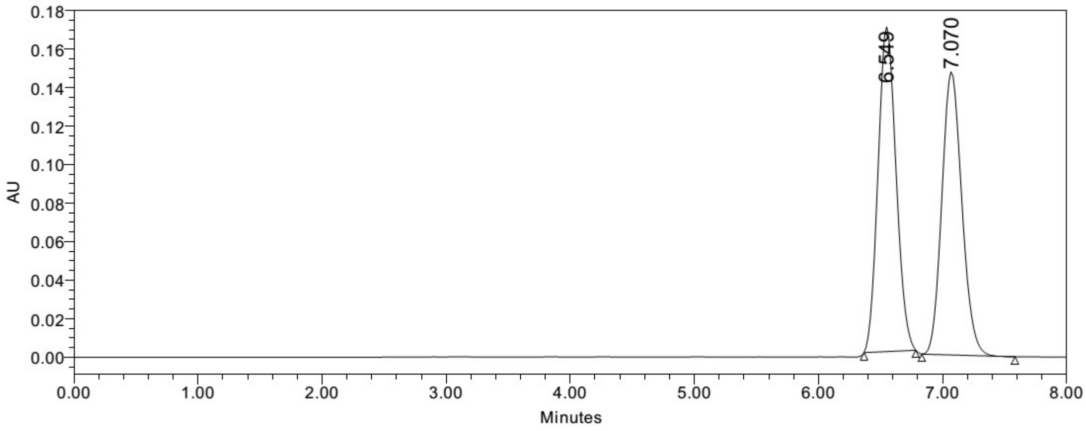

	RT	Area	% Area	Height
1	6.549	1703641	50.77	168331
2	7.070	1651644	49.23	147047

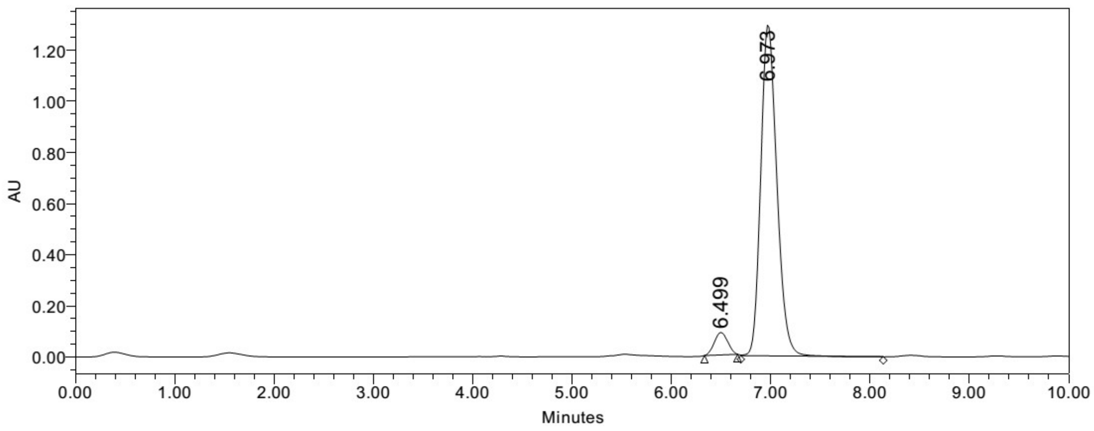

	RT	Area	% Area	Height
1	6.499	823301	5.34	88337
2	6.973	14600432	94.66	1298414

Supplementary Figure 16. ^1H NMR and ^{13}C NMR spectra of **3h**

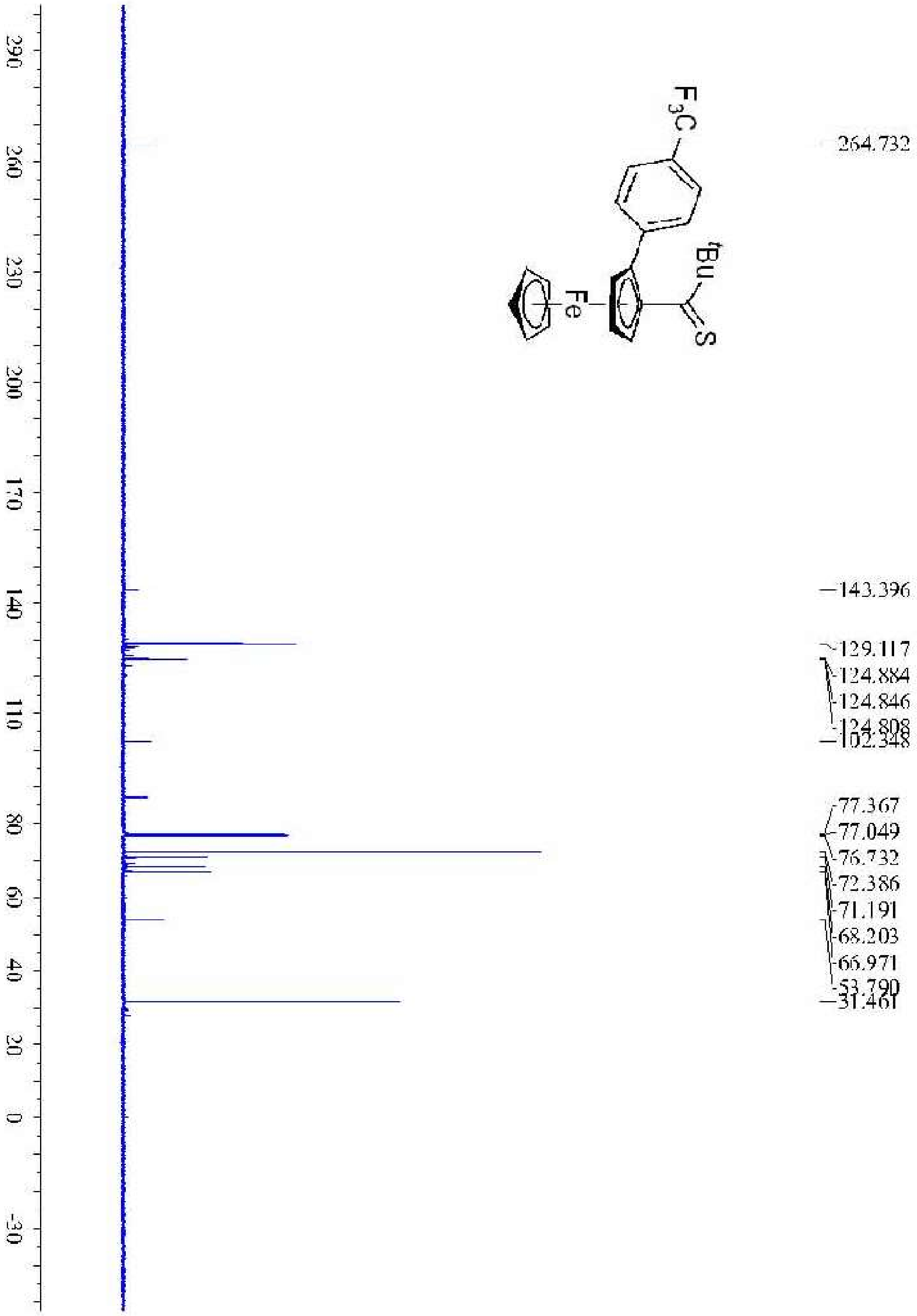

Supplementary Figure 17. ^{19}F NMR spectra of **3h**

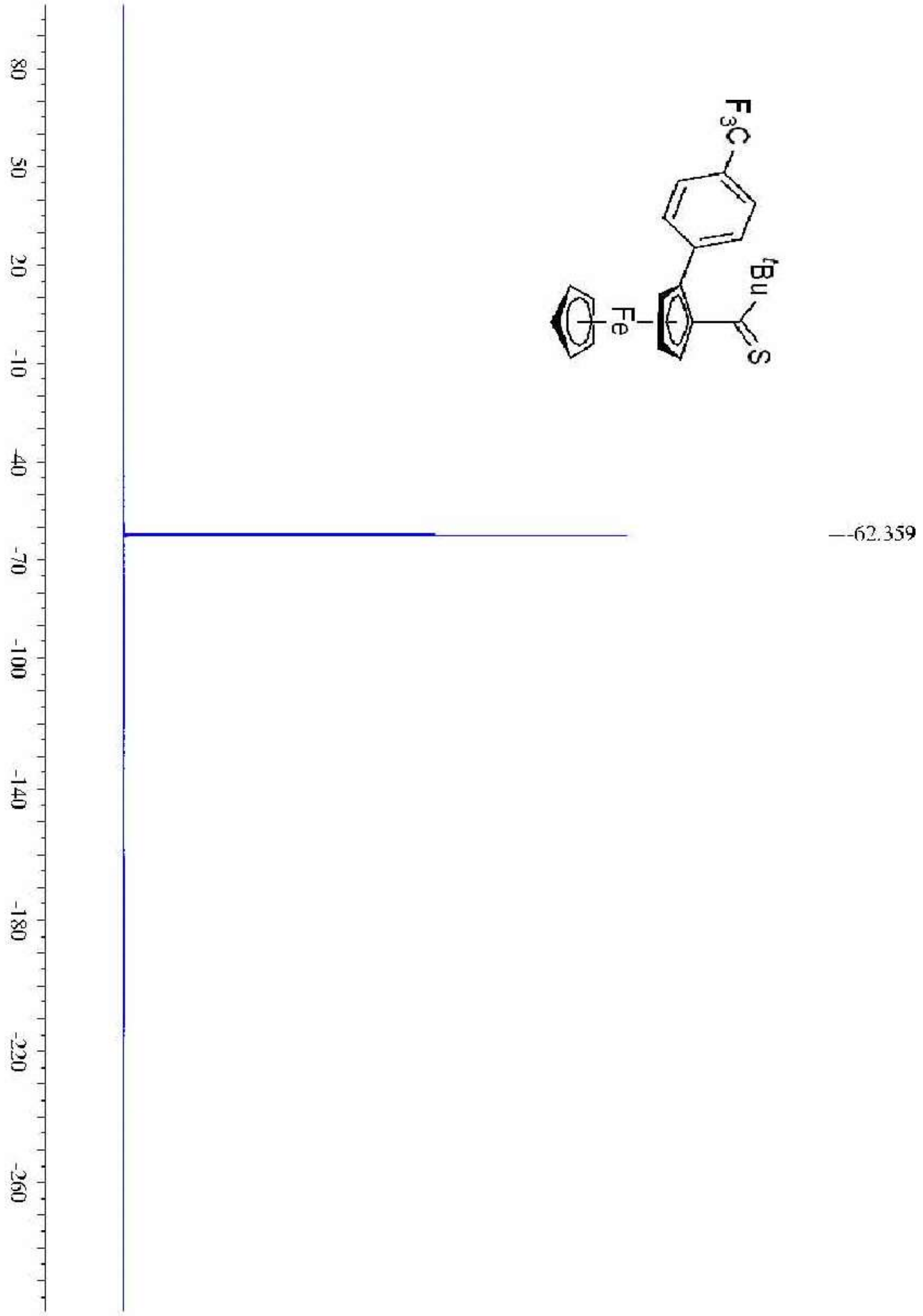

Supplementary Figure 18. HPLC analysis **3h**

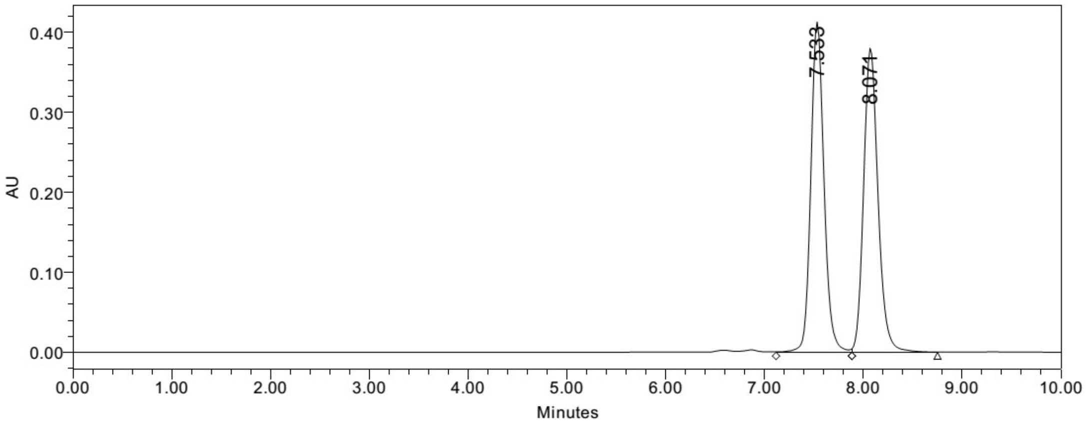

	RT	Area	% Area	Height
1	7.533	3811724	49.79	412241
2	8.071	3843552	50.21	380444

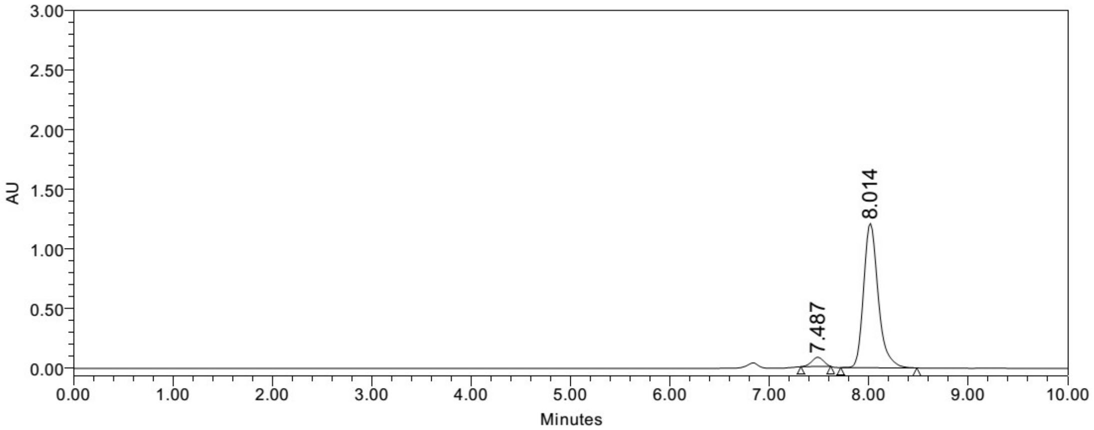

	RT	Area	% Area	Height
1	7.487	641119	4.93	77298
2	8.014	12359858	95.07	1211186

Supplementary Figure 19. ^{1}H NMR spectra of **3i**

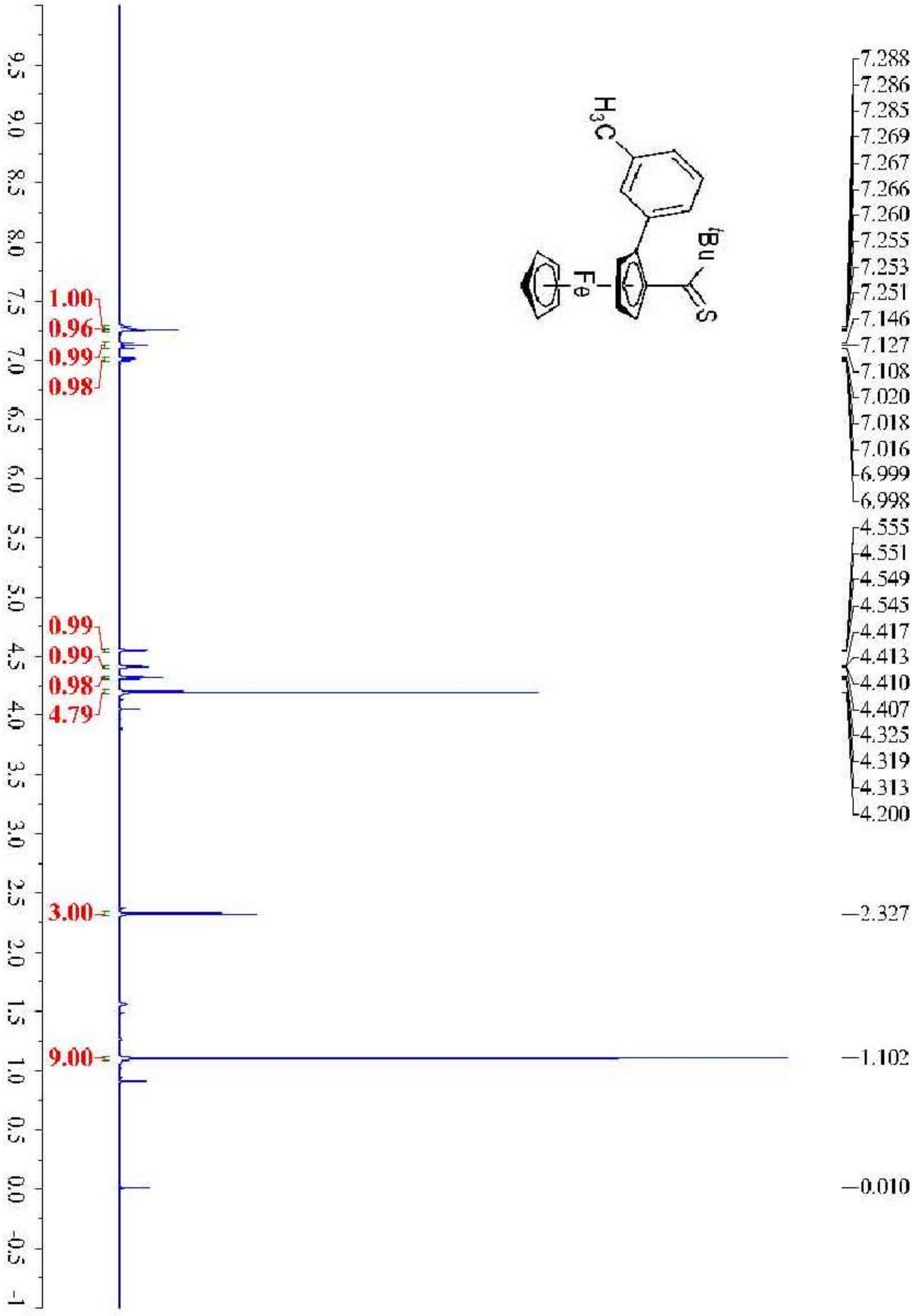

Supplementary Figure 20. HPLC analysis **3i**

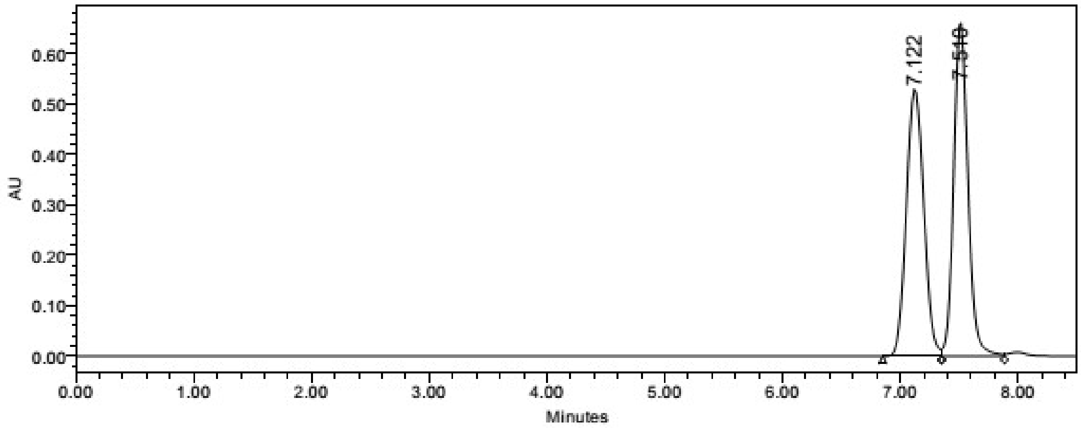

	RT	Area	% Area	Height
1	7.122	5476321	49.71	531368
2	7.510	5539396	50.29	664654

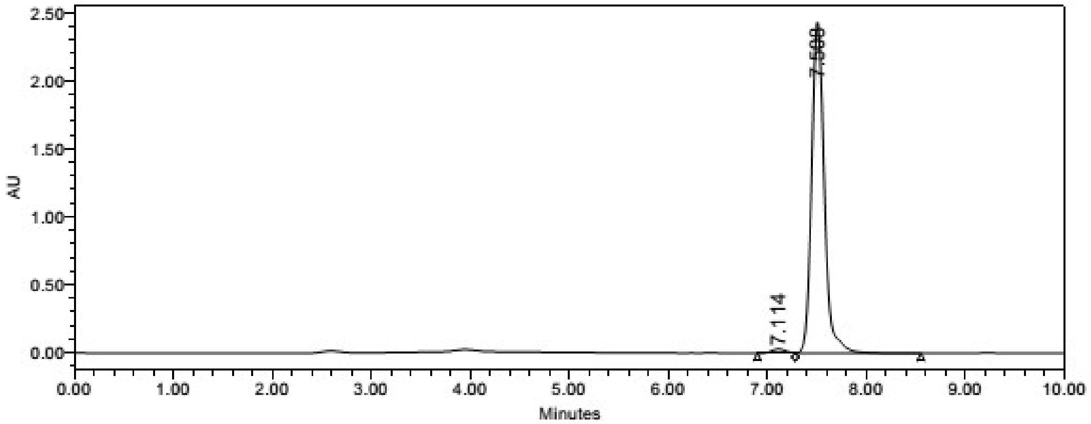

	RT	Area	% Area	Height
1	7.114	323366	1.47	31455
2	7.508	21685695	98.53	2449853

Supplementary Figure 21. ^{1}H NMR spectra of **3j**

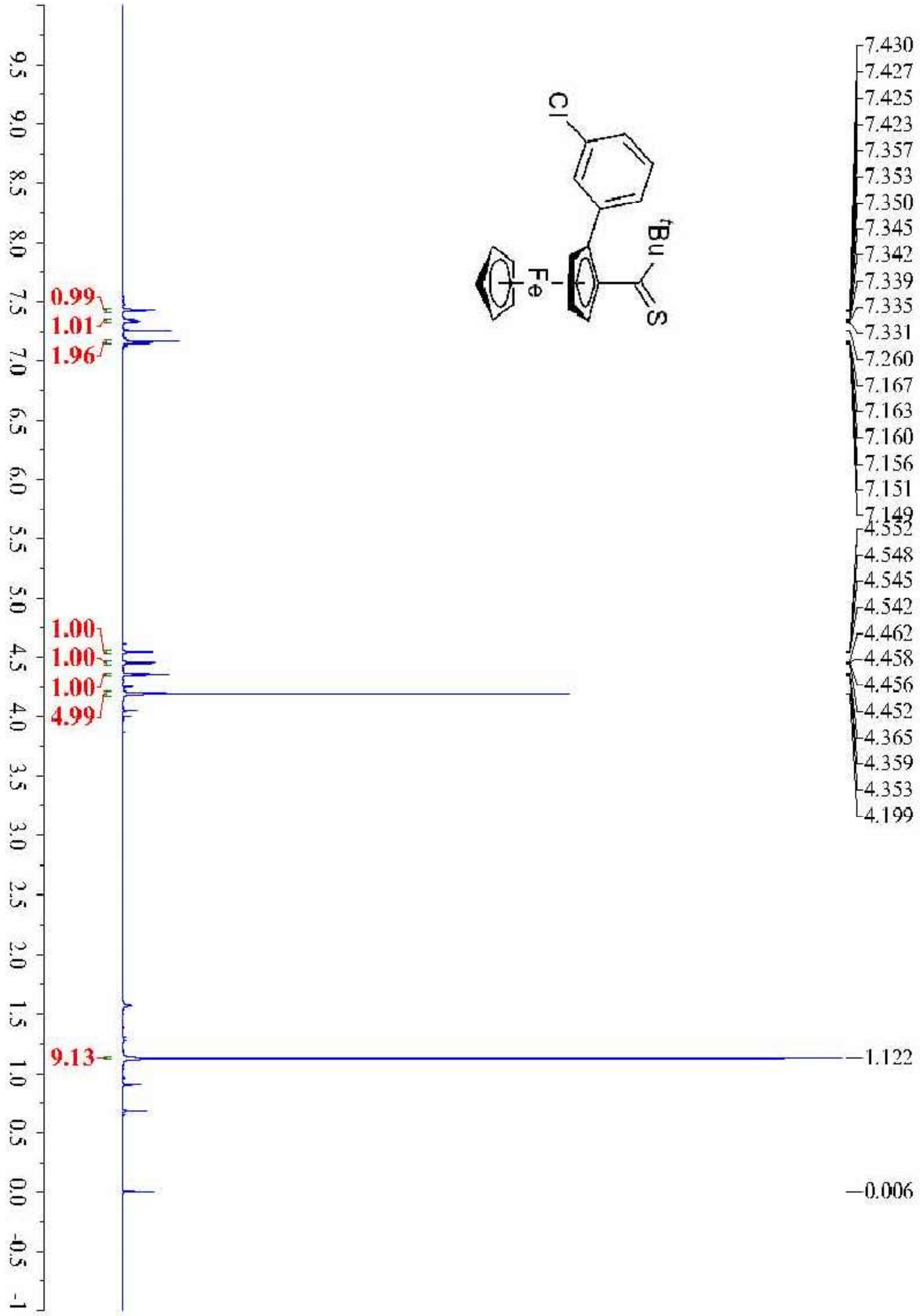

Supplementary Figure 22. HPLC analysis **3j**

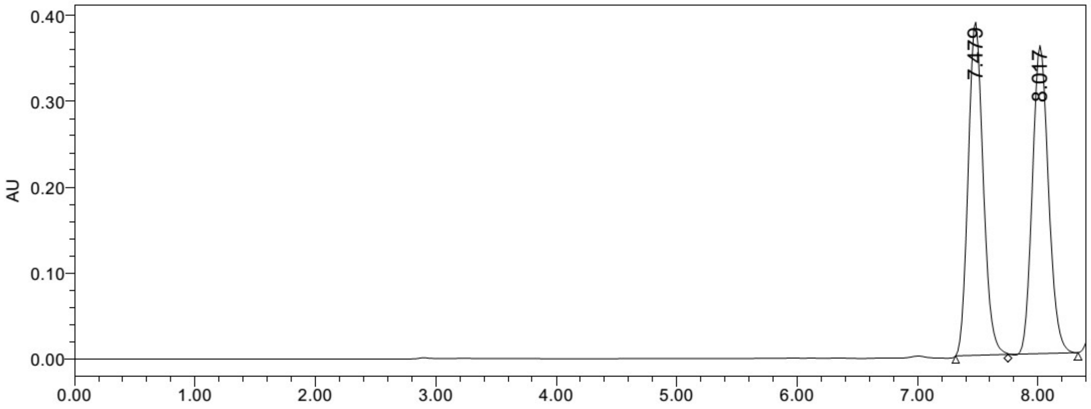

	RT	Area	% Area	Height
1	7.479	3361309	50.12	388246
2	8.017	3344971	49.88	358580

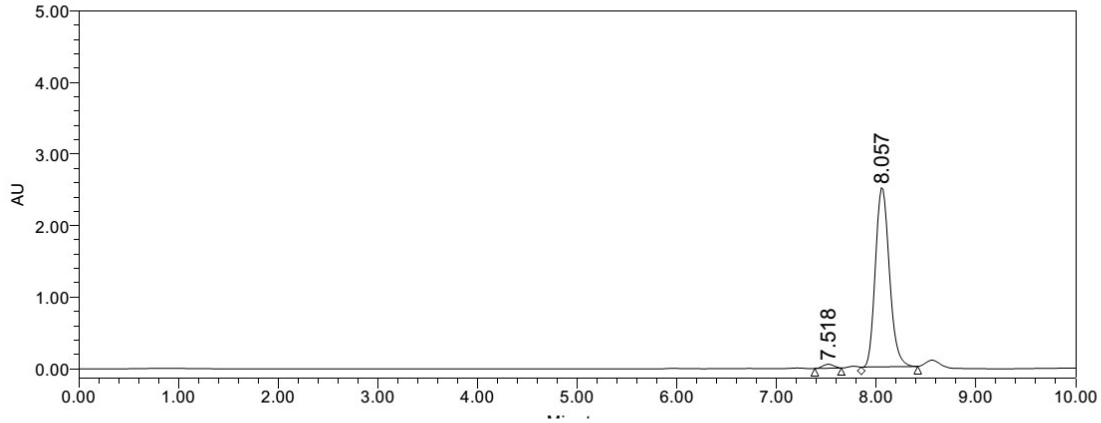

	RT	Area	% Area	Height
1	7.518	424802	1.70	56008
2	8.057	24537316	98.30	2513604

Supplementary Figure 23. ^{1}H NMR and ^{13}C NMR spectra of **3k**

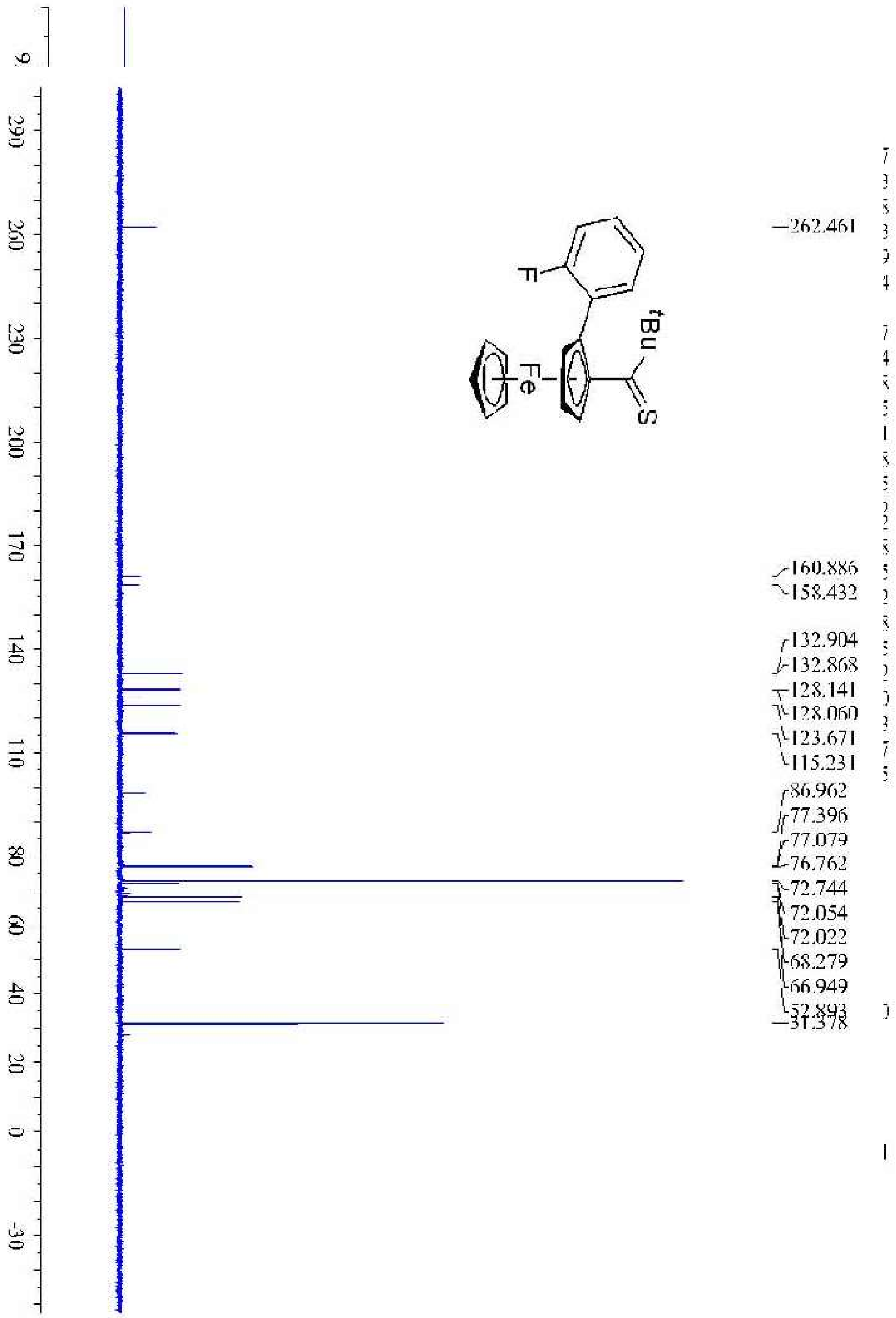

Supplementary Figure 24. [1]F NMR spectra of **3k**

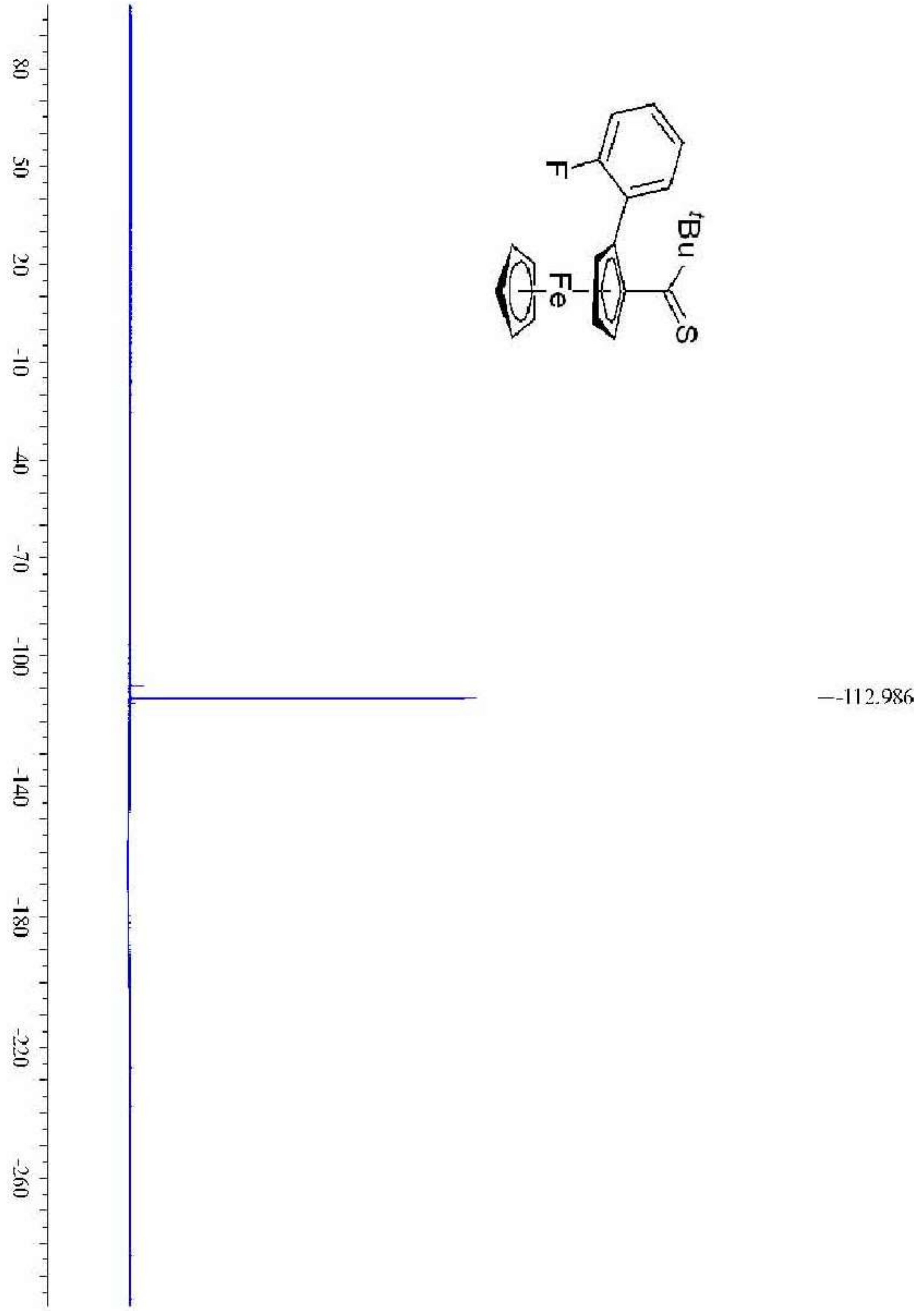

Supplementary Figure 25. HPLC analysis **3k**

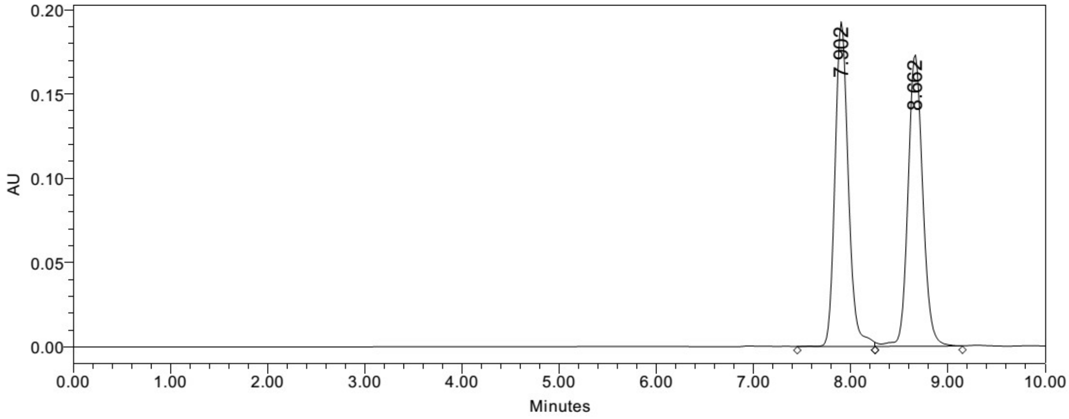

	RT	Area	% Area	Height
1	7.902	1832943	50.04	192577
2	8.662	1830086	49.96	173196

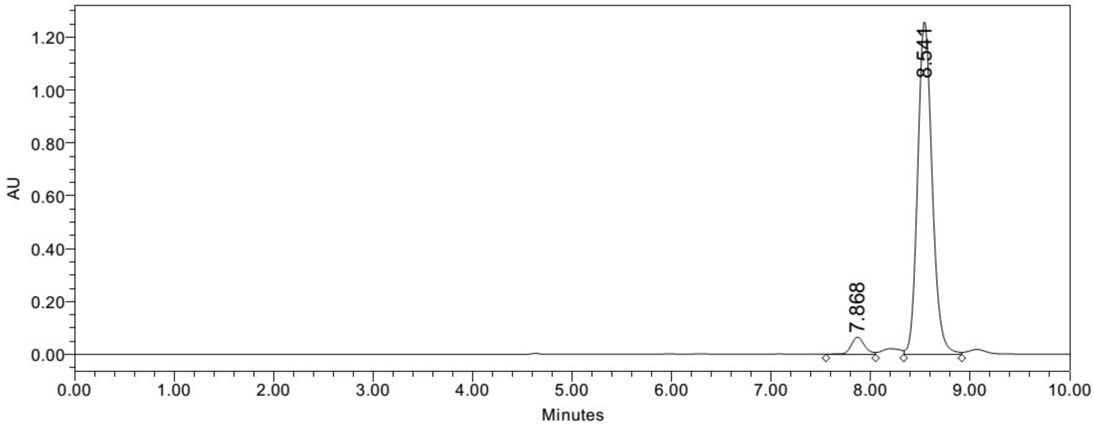

	RT	Area	% Area	Height
1	7.868	600153	4.50	64623
2	8.541	12737282	95.50	1263567

Supplementary Figure 26. ^{1}H NMR spectra of **3l**

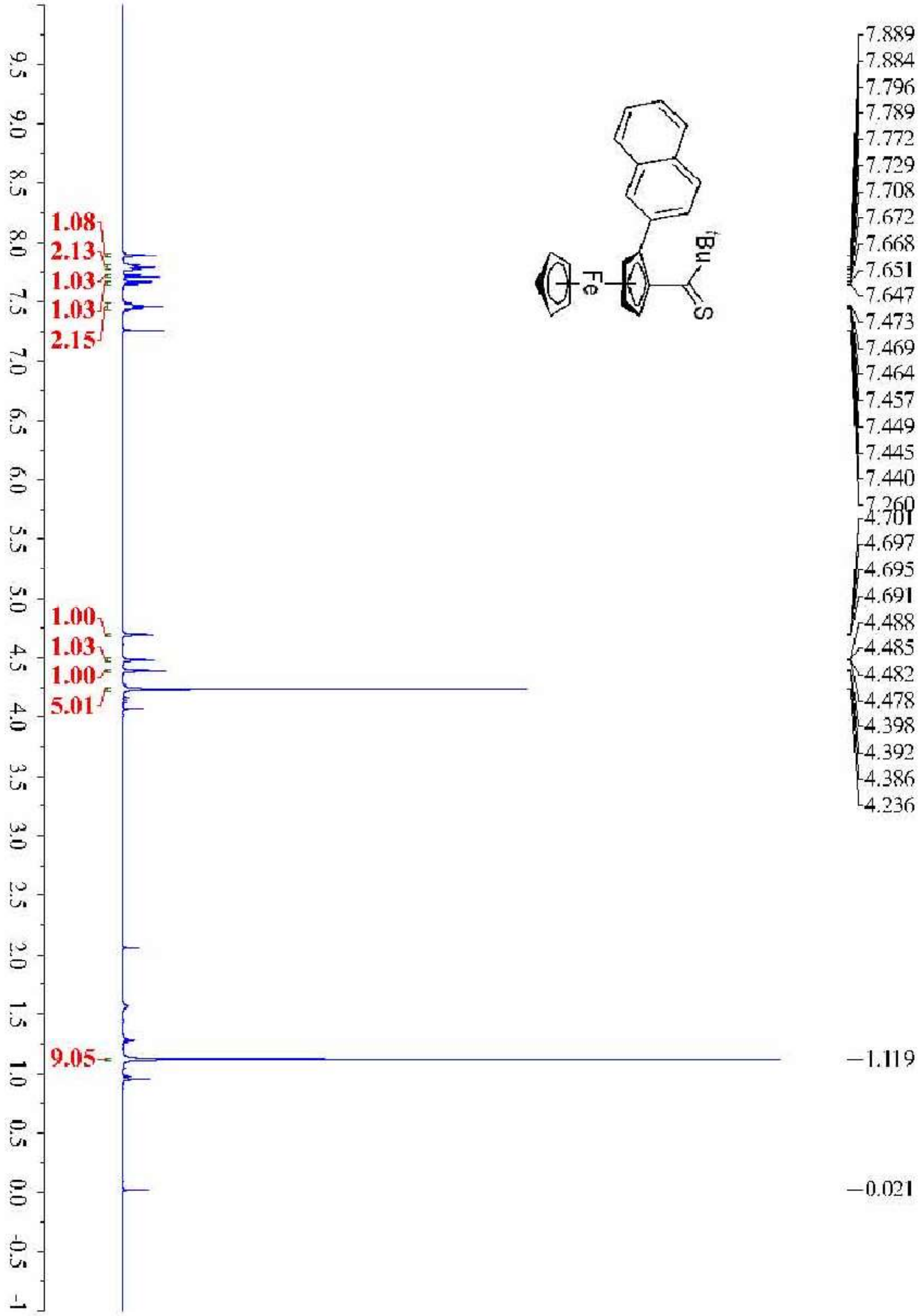

Supplementary Figure 27. HPLC analysis **3l**

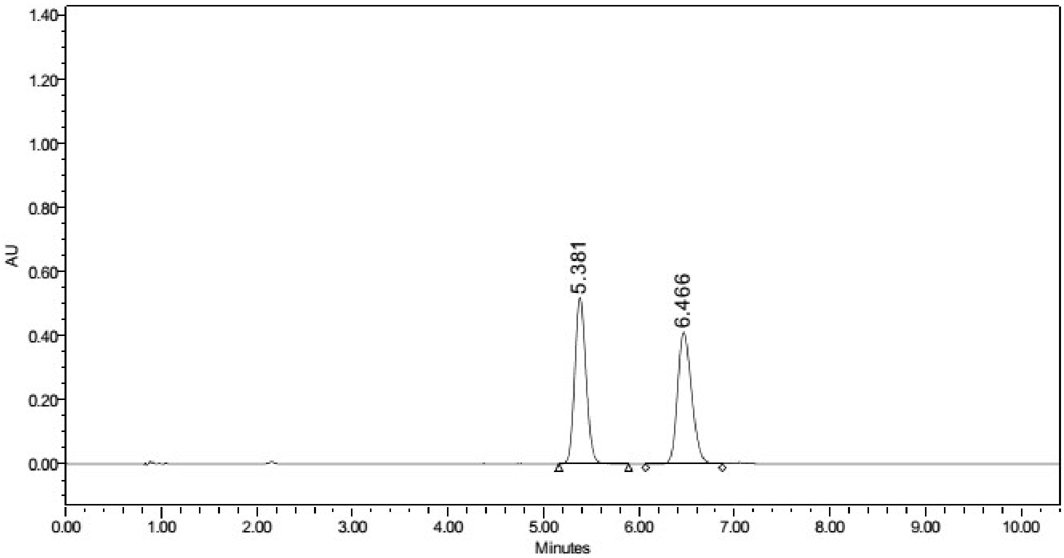

	RT	Peak Type	Height	Width (sec)	Area	% Area
1	5.381	Unknown	519364	43.800	4288084	50.23
2	6.466	Unknown	410485	48.250	4249229	49.77

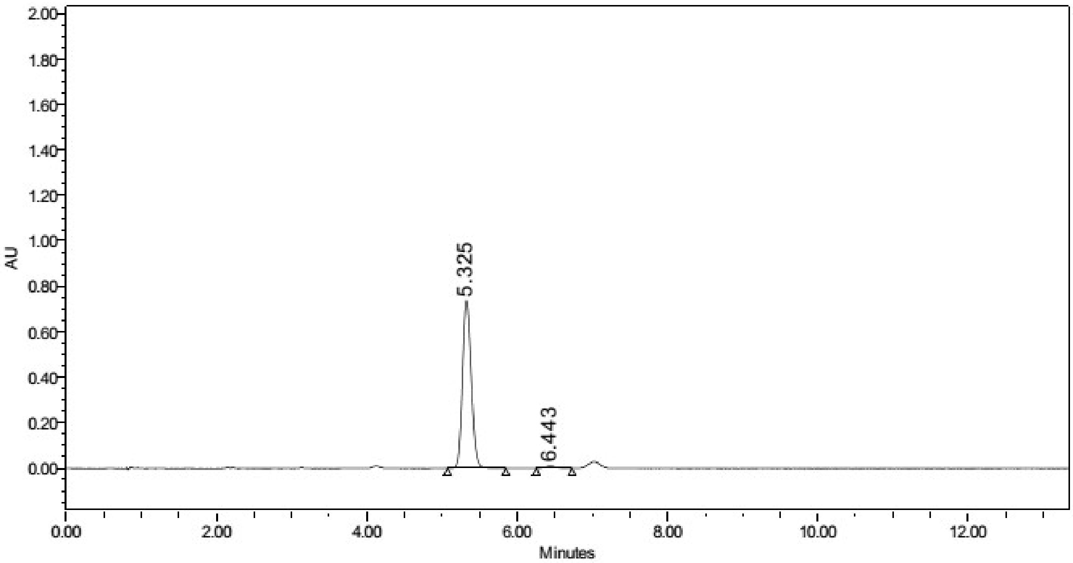

	RT	Peak Type	Height	Width (sec)	Area	% Area
1	5.325	Unknown	738310	46.450	6033047	98.48
2	6.443	Unknown	9419	28.300	93418	1.52

Supplementary Figure 28. ^{1}H NMR and ^{13}C NMR spectra of **3m**

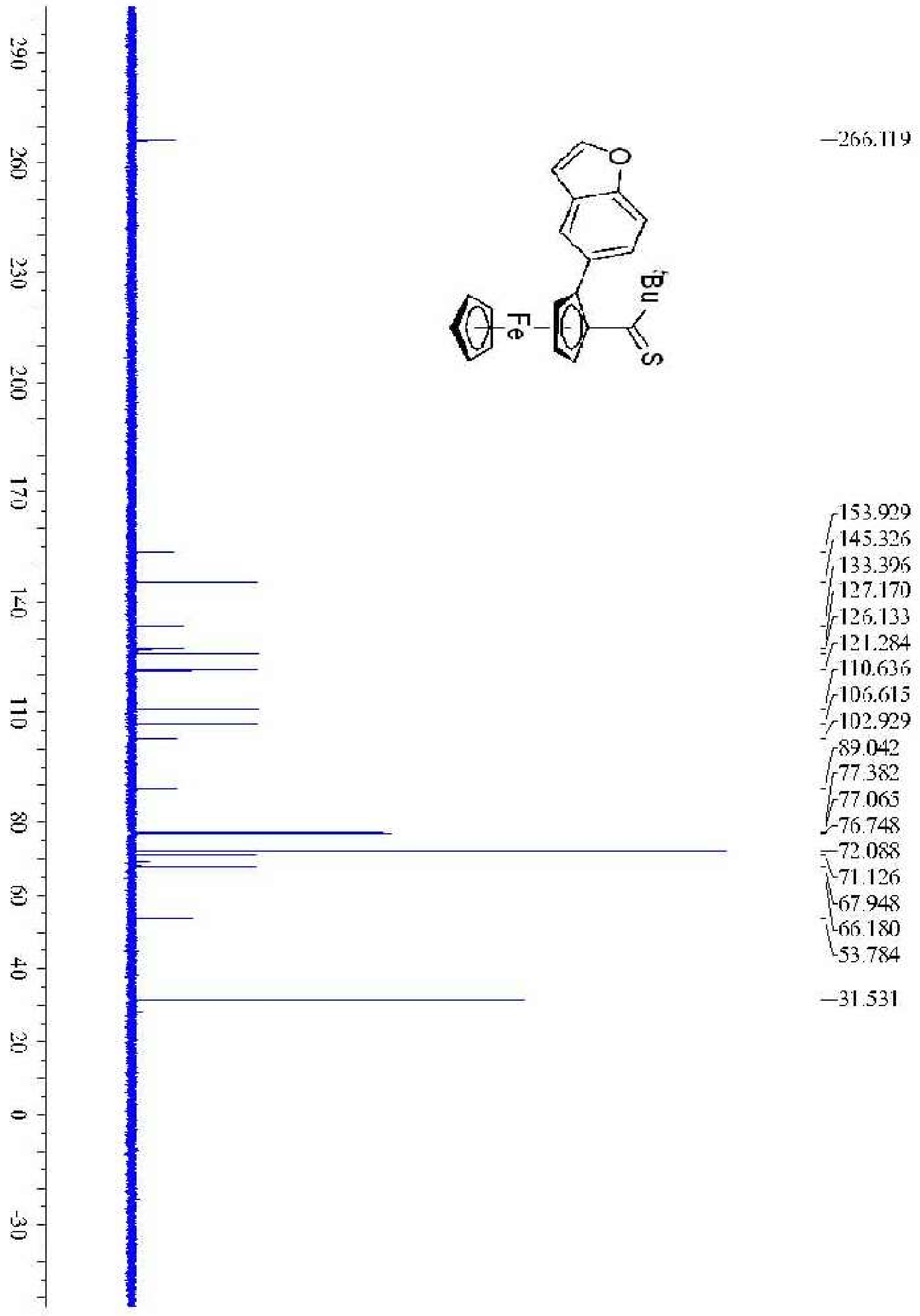

Supplementary Figure 29. HPLC analysis **3m**

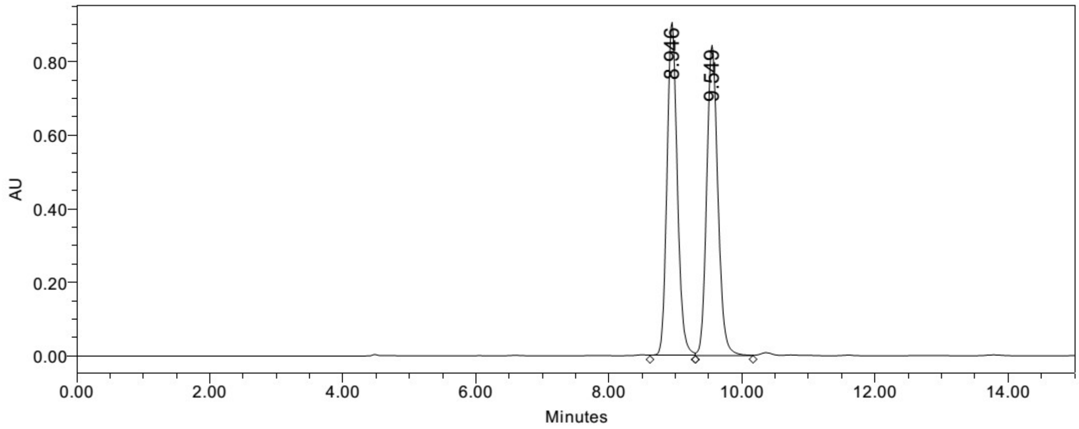

	RT	Area	% Area	Height
1	8.946	9784432	49.80	906672
2	9.549	9863336	50.20	843422

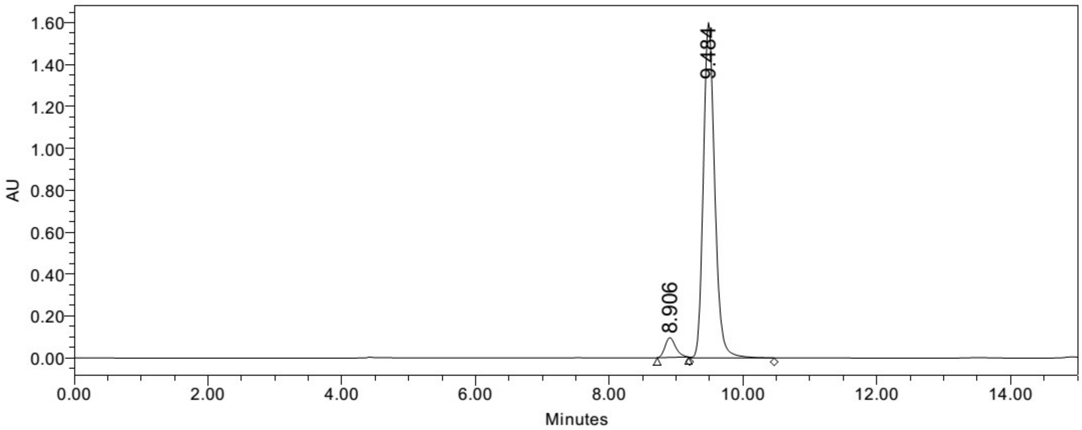

	RT	Area	% Area	Height
1	8.906	1043539	5.21	94871
2	9.484	18974550	94.79	1600982

Supplementary Figure 30. ^{1}H NMR and ^{13}C NMR spectra of **3n**

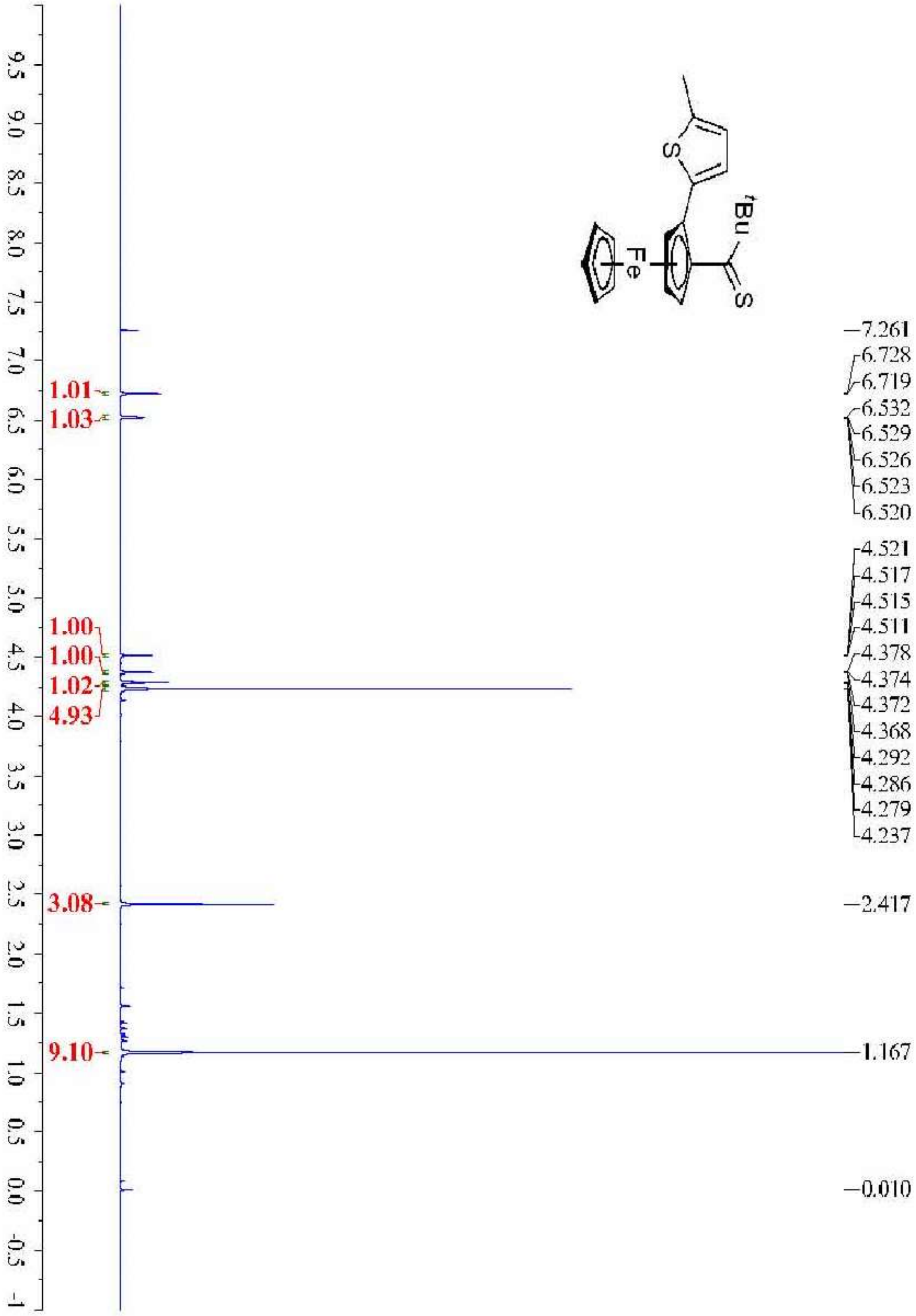

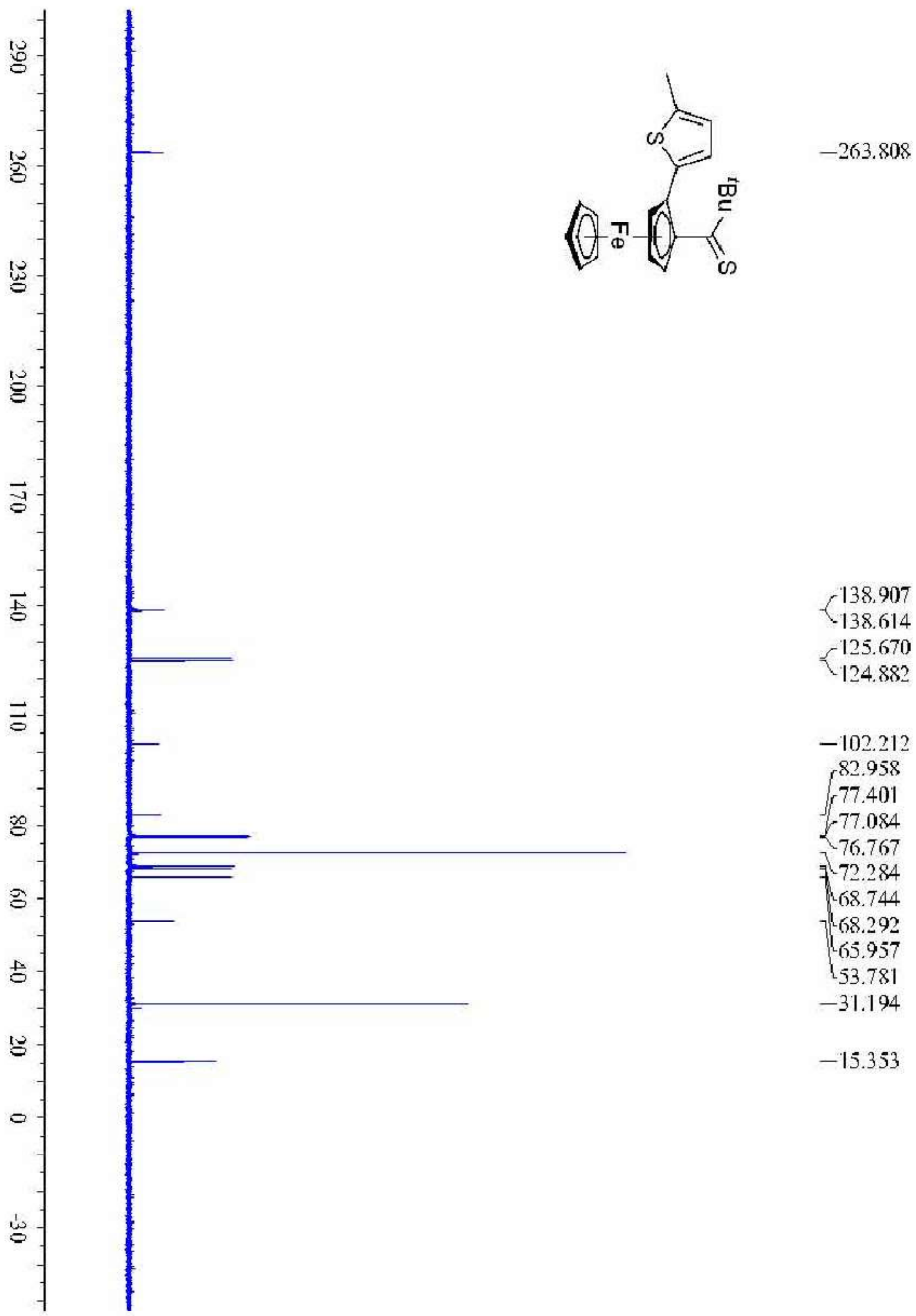
263.808
138.907
138.614
125.670
124.882
102.212
82.958
77.401
77.084
76.767
72.284
68.744
68.292
65.957
53.781
31.194
15.353
290
260
230
200
170
140
110
80
60
40
20
0
-30
Fe
S
S
tBu

Supplementary Figure 31. HPLC analysis **3n**

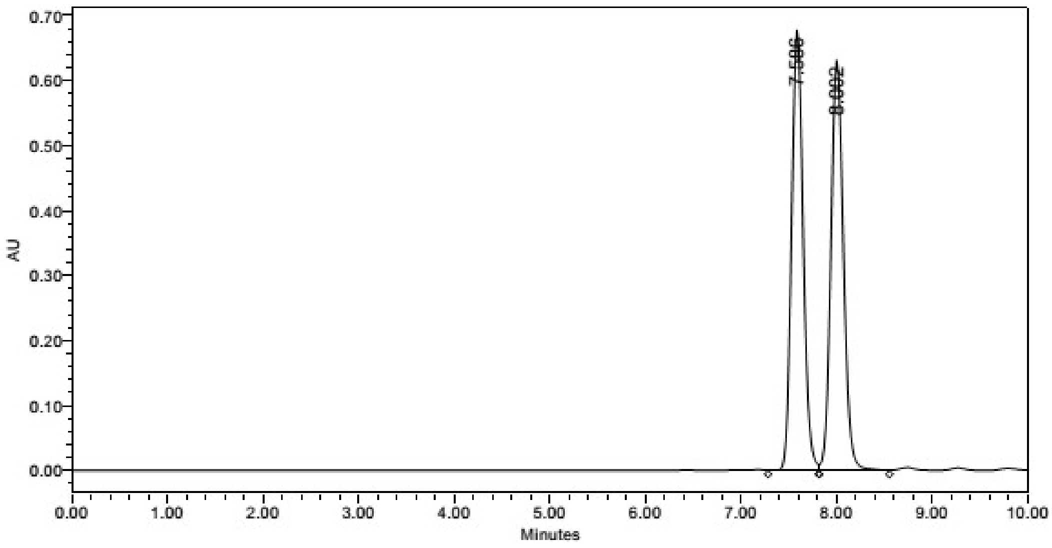

	RT	Area	% Area	Height
1	7.586	5688563	49.88	677162
2	8.002	5716515	50.12	630752

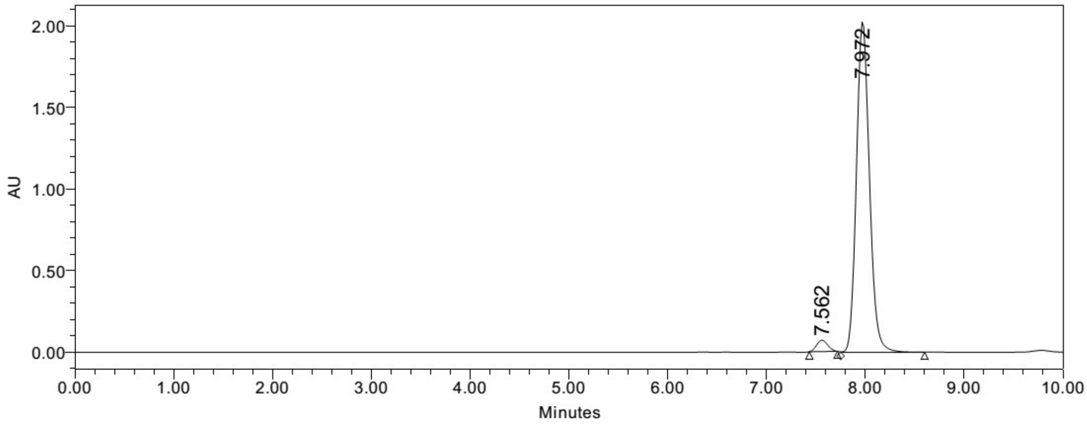

	RT	Area	% Area	Height
1	7.562	551803	2.89	70251
2	7.972	18536865	97.11	2028454

Supplementary Figure 32. ^{1}H NMR and ^{13}C NMR spectra of **3o**

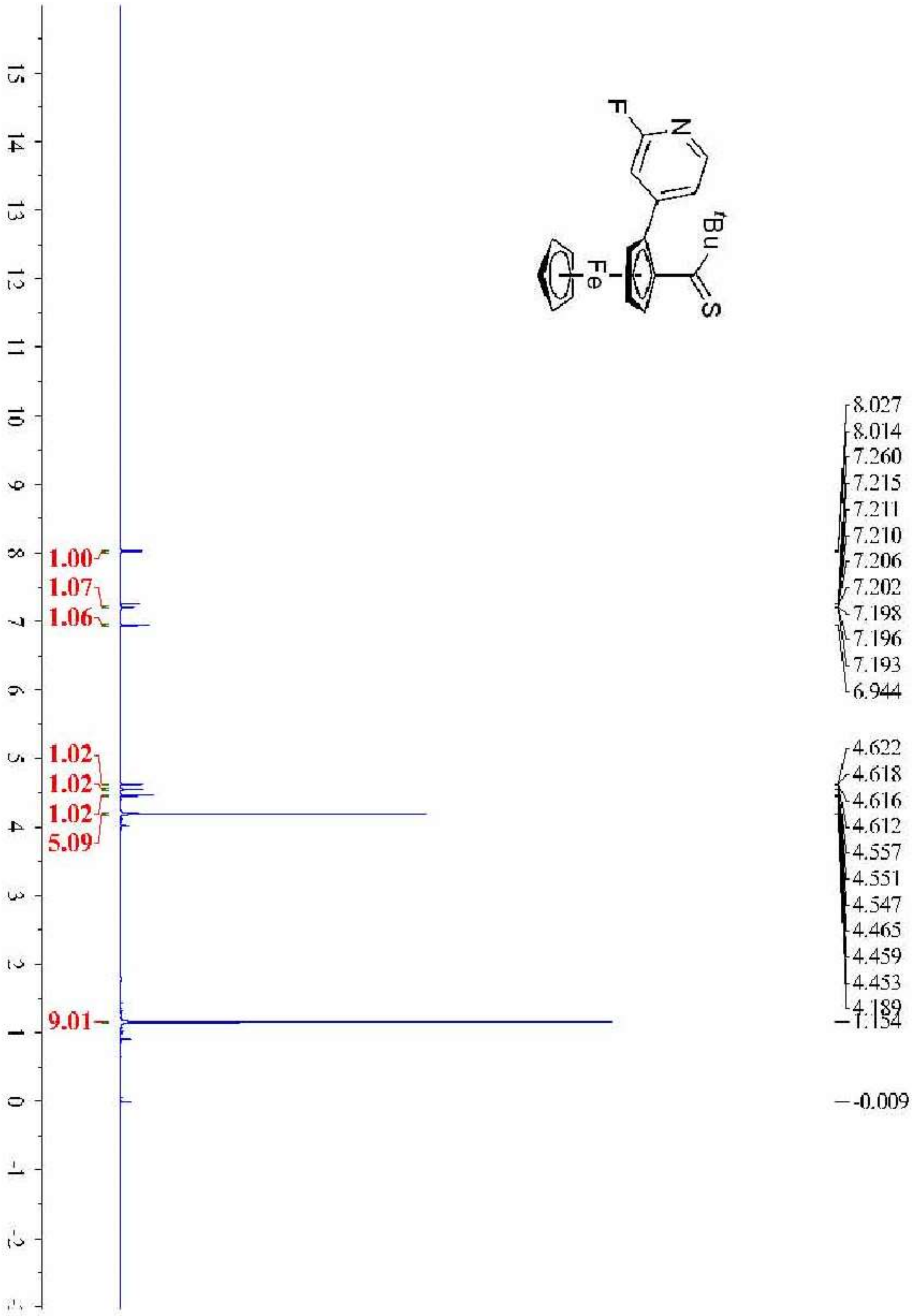

Supplementary Figure 33. ^{19}F NMR spectra of **3o**

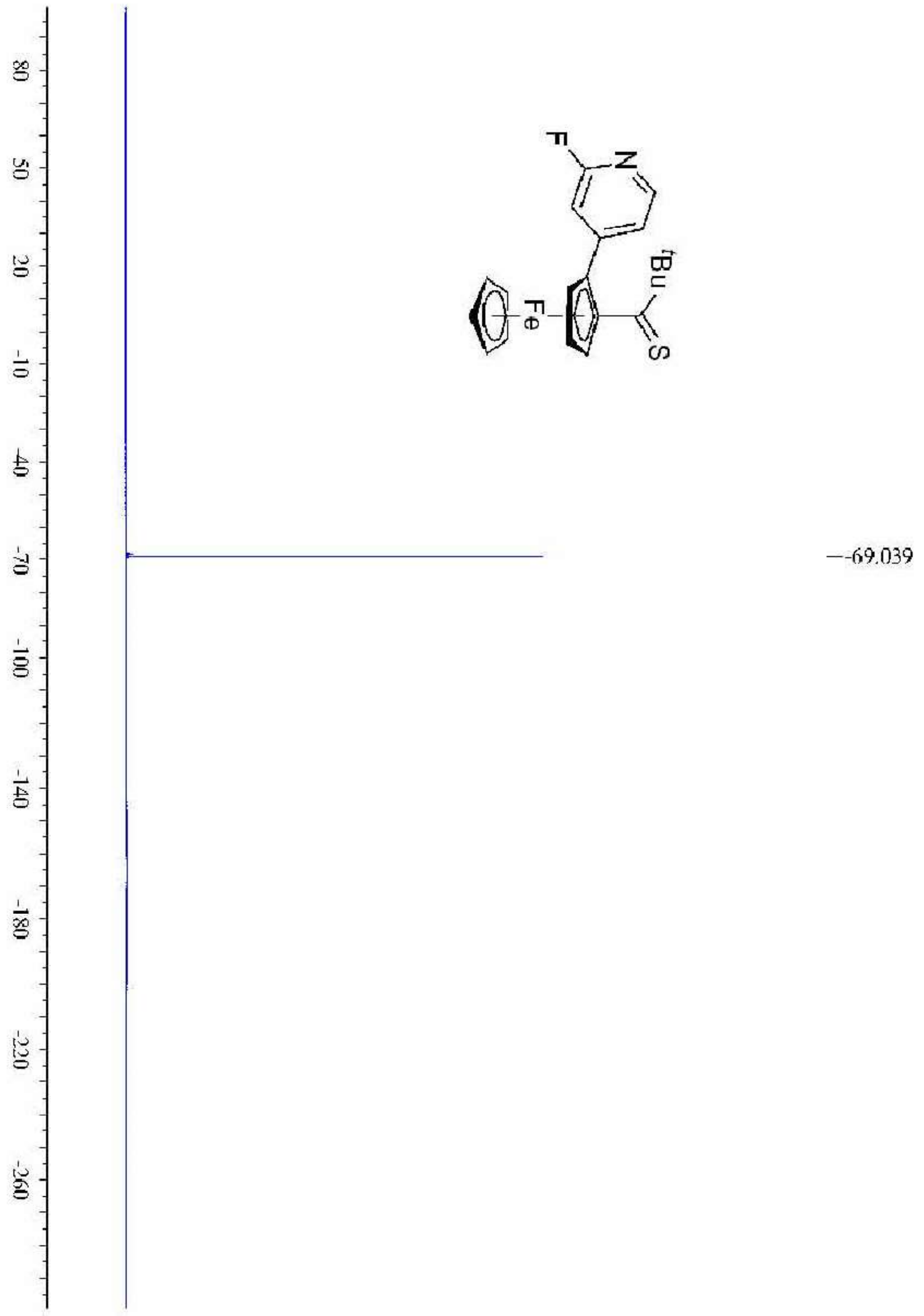

Supplementary Figure 34. HPLC analysis **3o**

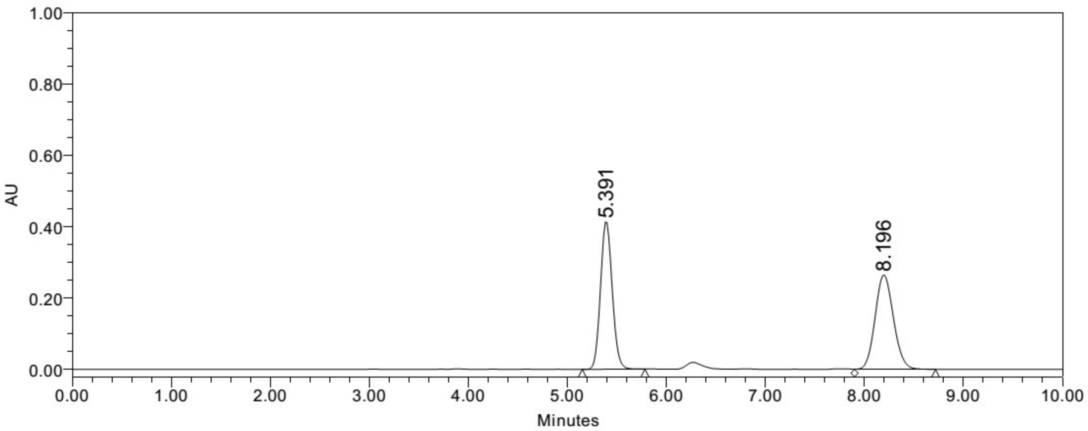

	RT	Area	% Area	Height
1	5.391	3410189	49.96	415773
2	8.196	3416298	50.04	265026

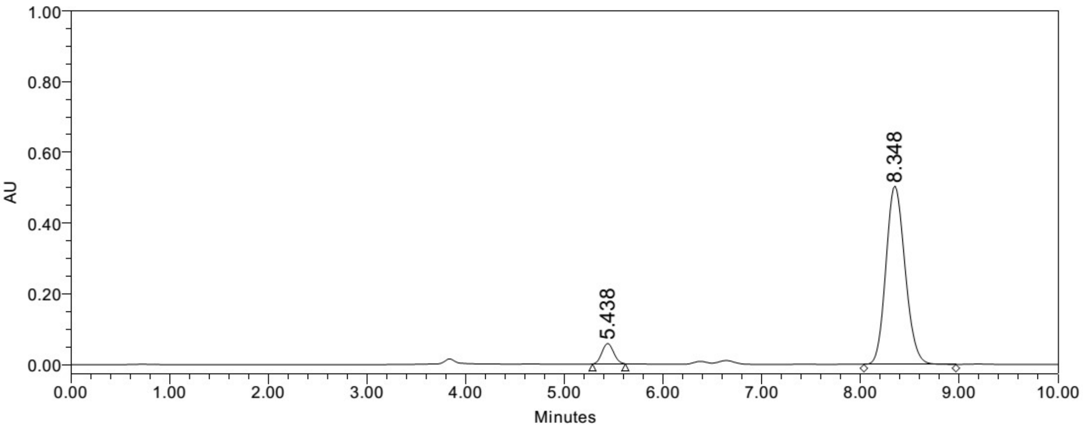

	RT	Area	% Area	Height
1	5.438	473022	6.48	57806
2	8.348	6831826	93.52	504629

Supplementary Figure 35. ^{1}H NMR and ^{13}C NMR spectra of **3p**

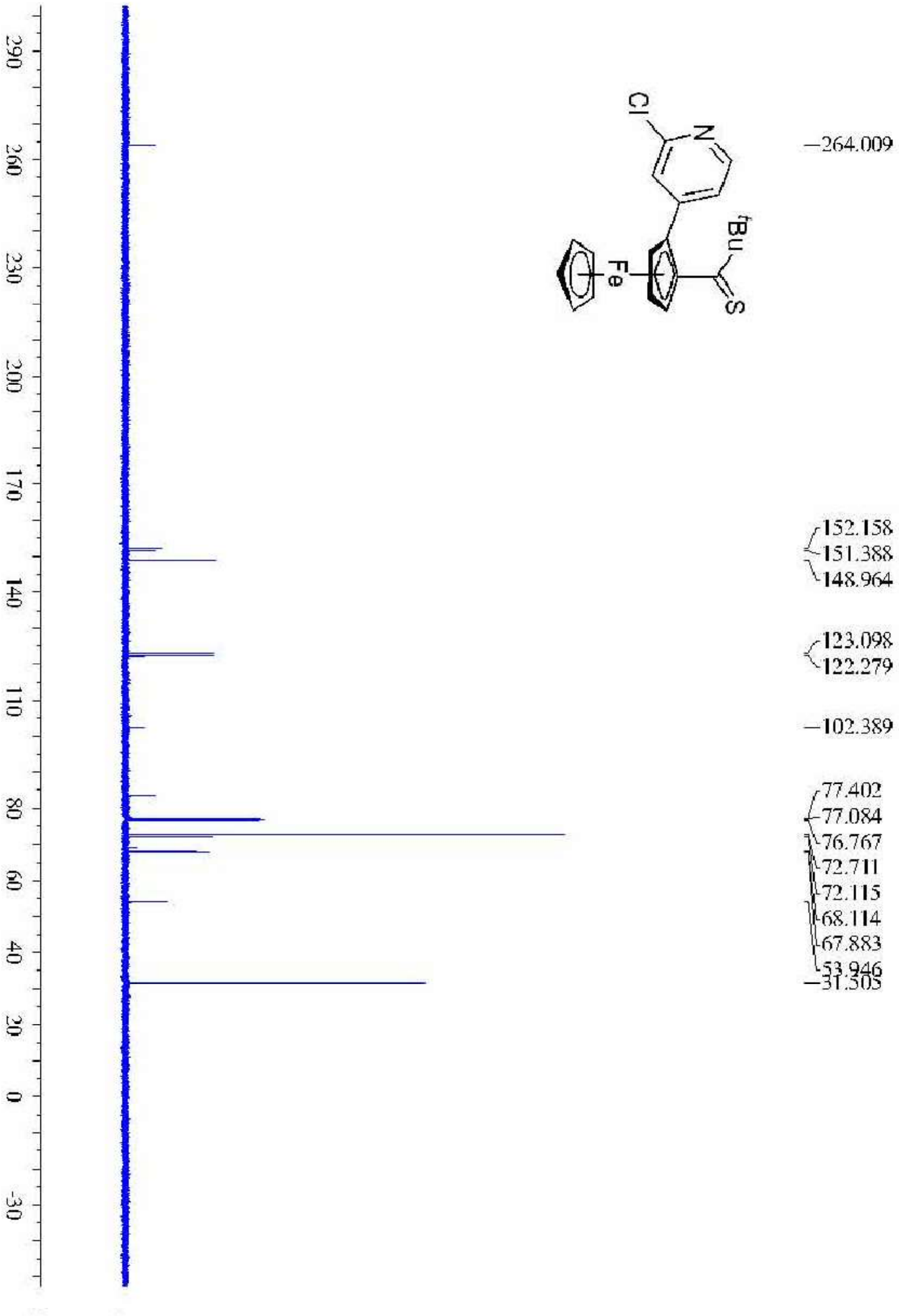

Supplementary Figure 36. HPLC analysis **3p**

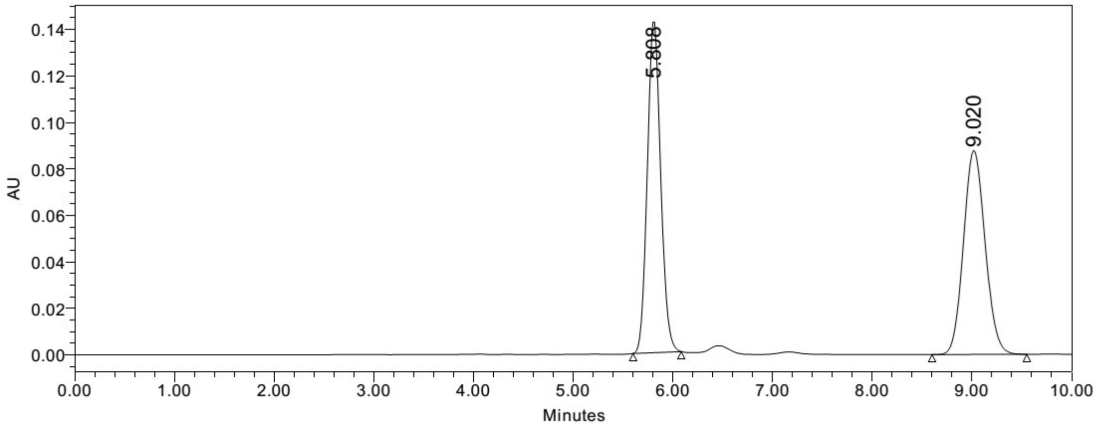

	RT	Area	% Area	Height
1	5.808	1330332	50.12	143314
2	9.020	1324132	49.88	87826

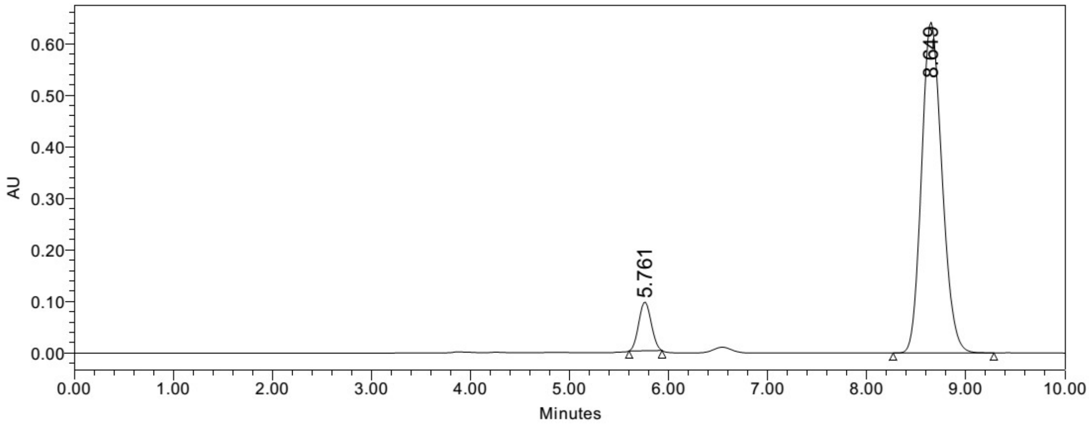

	RT	Area	% Area	Height
1	5.761	829473	8.26	94691
2	8.649	9217345	91.74	641877

Supplementary Figure 37. ^{1}H NMR and ^{13}C NMR spectra of **3q**

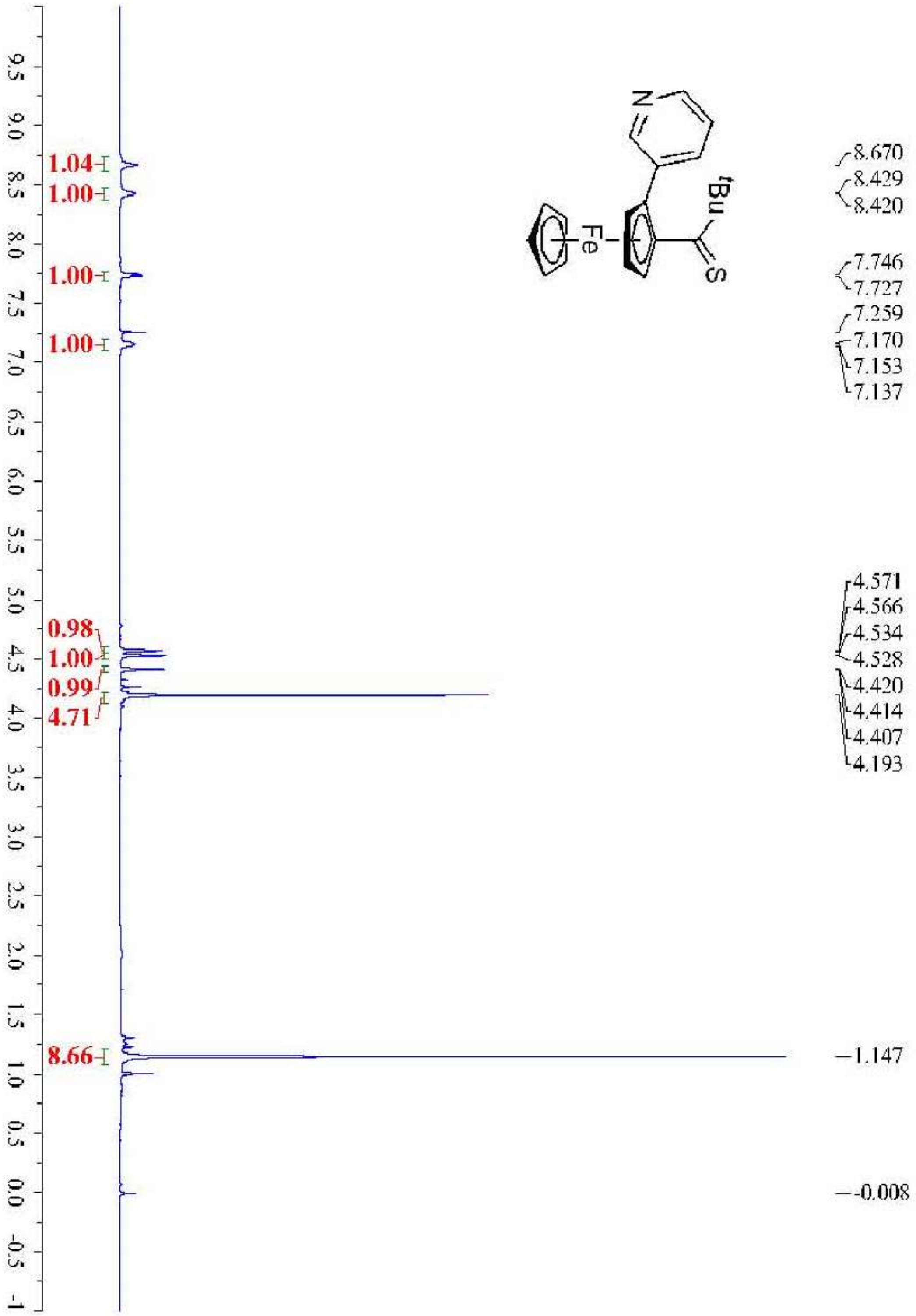

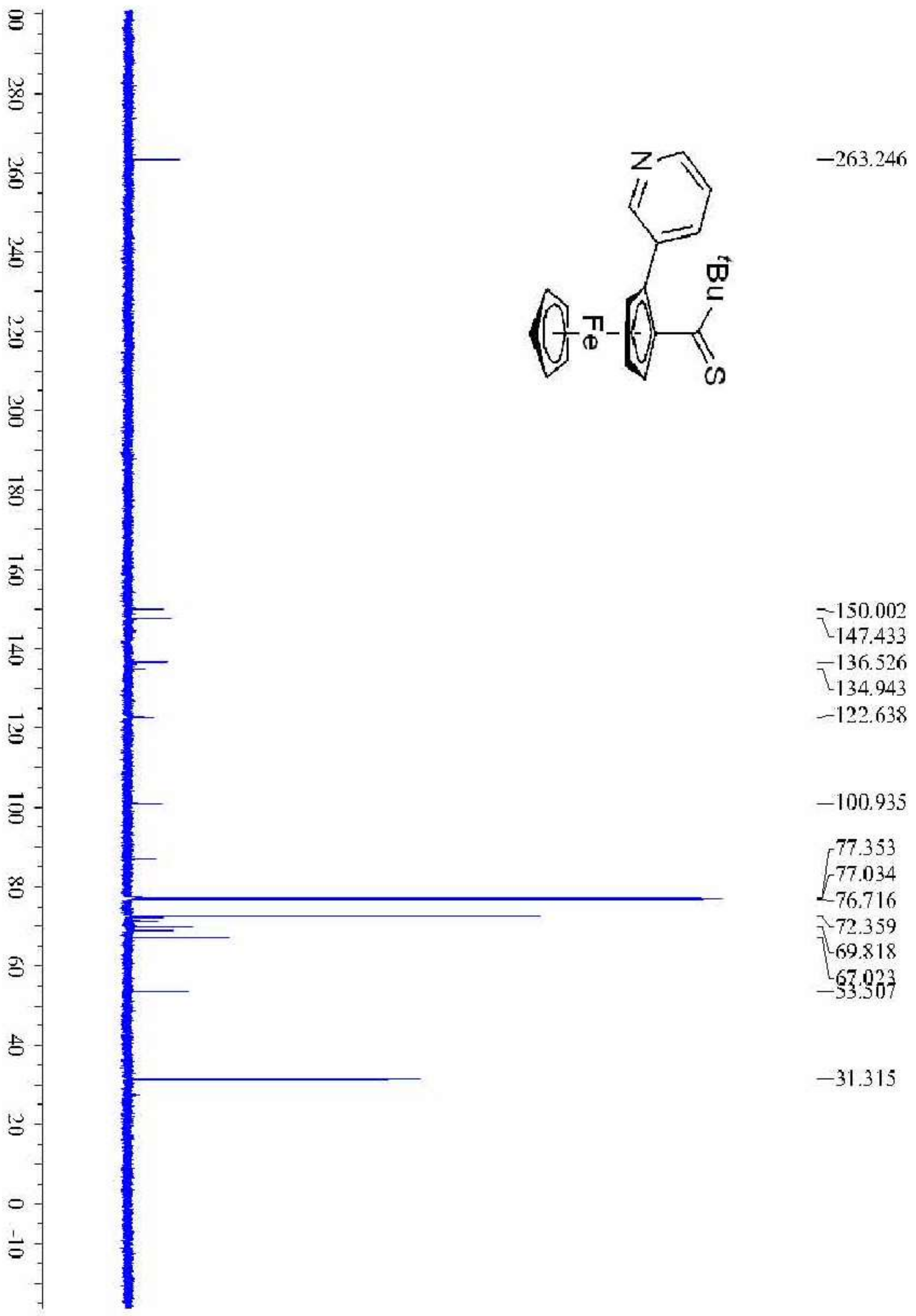

263.246
150.002
147.433
136.526
134.943
122.638
100.935
77.353
77.034
76.716
72.359
69.818
67.023
53.507
31.315
Fe
S
tBu
N

Supplementary Figure 38. HPLC analysis **3q**

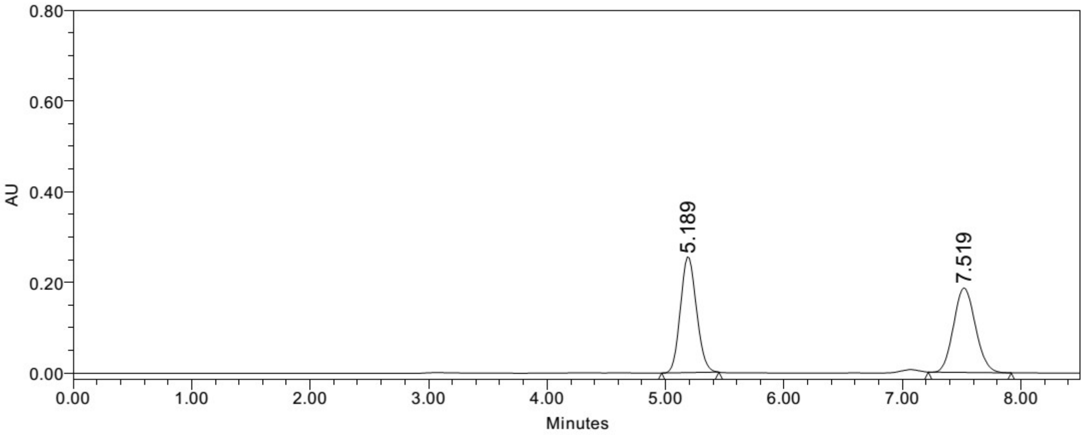

	RT	Area	% Area	Height
1	5.189	2414555	50.14	256423
2	7.519	2401315	49.86	186898

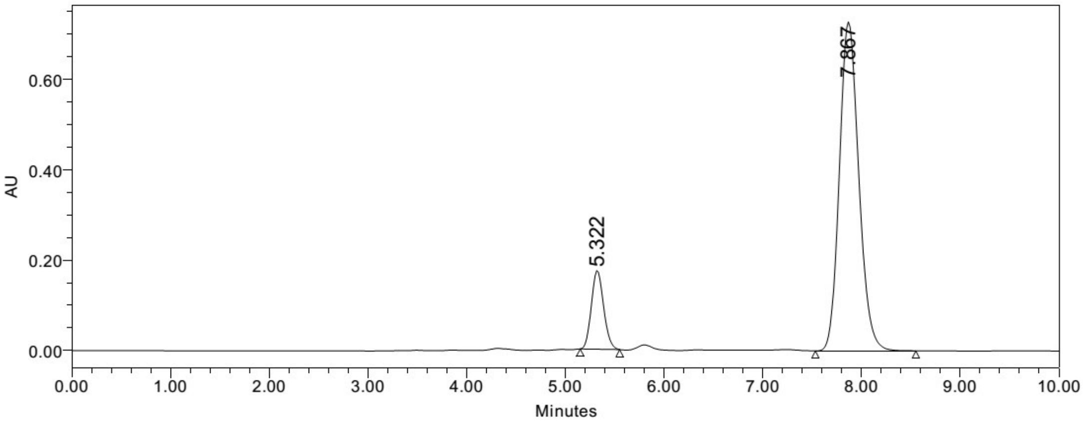

	RT	Area	% Area	Height
1	5.322	1507238	13.19	174379
2	7.867	9920226	86.81	728033

Supplementary Figure 39. ^{1}H NMR spectra of **3r**

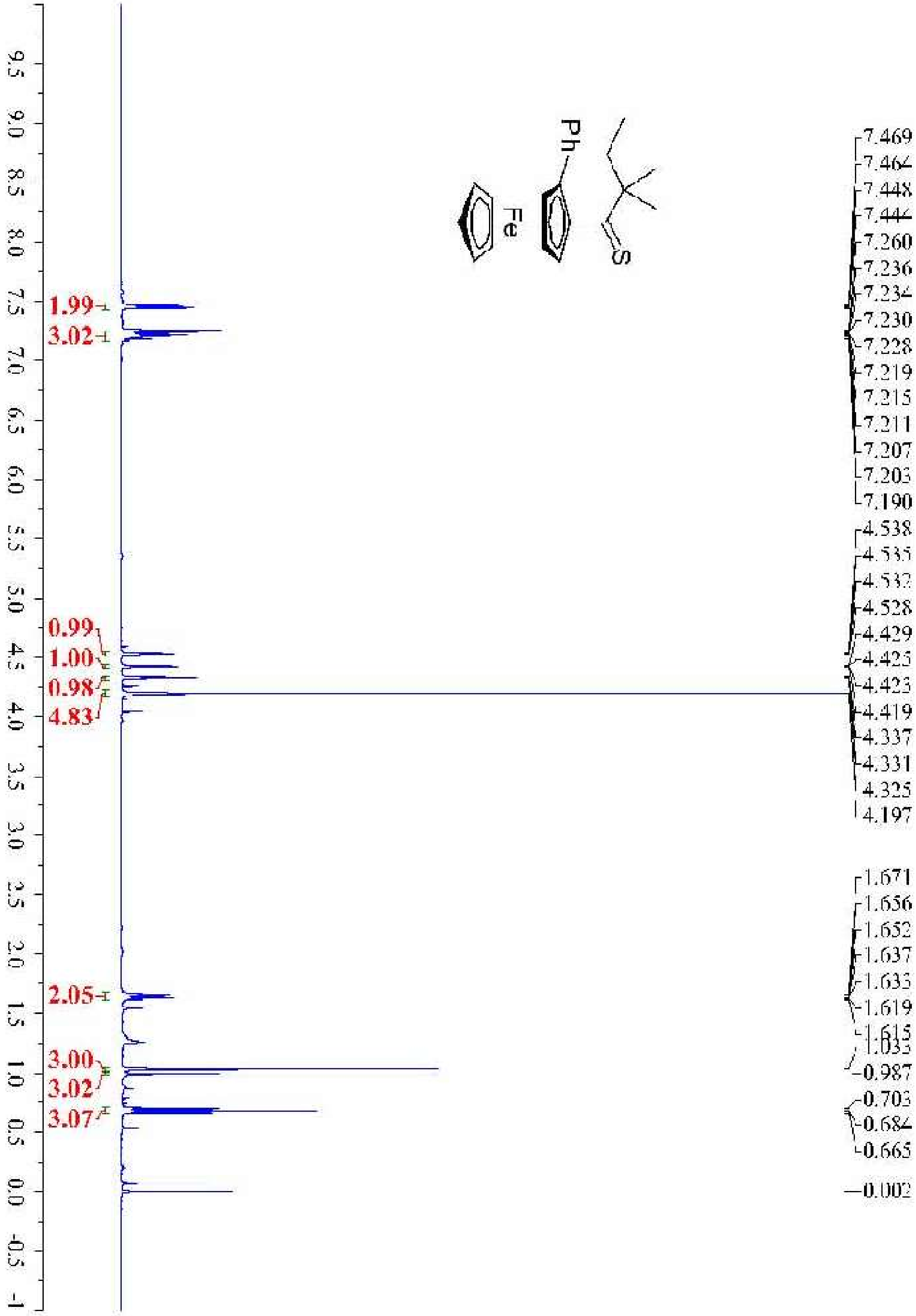

Supplementary Figure 40. HPLC analysis **3r**

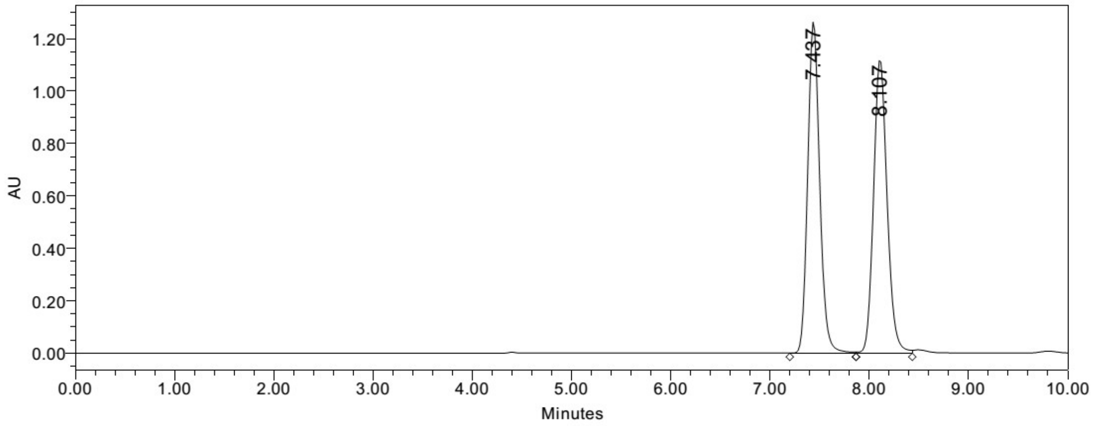

	RT	Area	% Area	Height
1	7.437	10606880	49.92	1263273
2	8.107	10641831	50.08	1121571

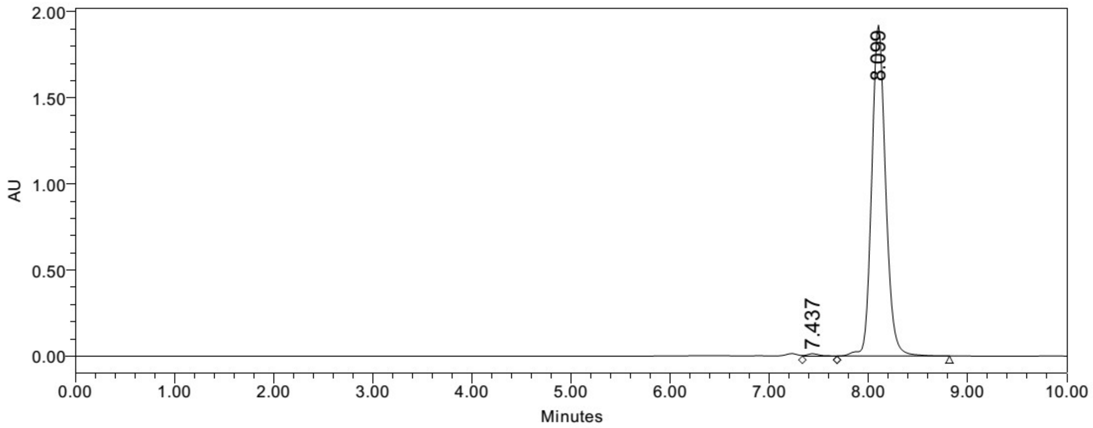

	RT	Area	% Area	Height
1	7.437	117013	0.62	13473
2	8.099	18798172	99.38	1920668

Supplementary Figure 41. ^{1}H NMR spectra of **3s**

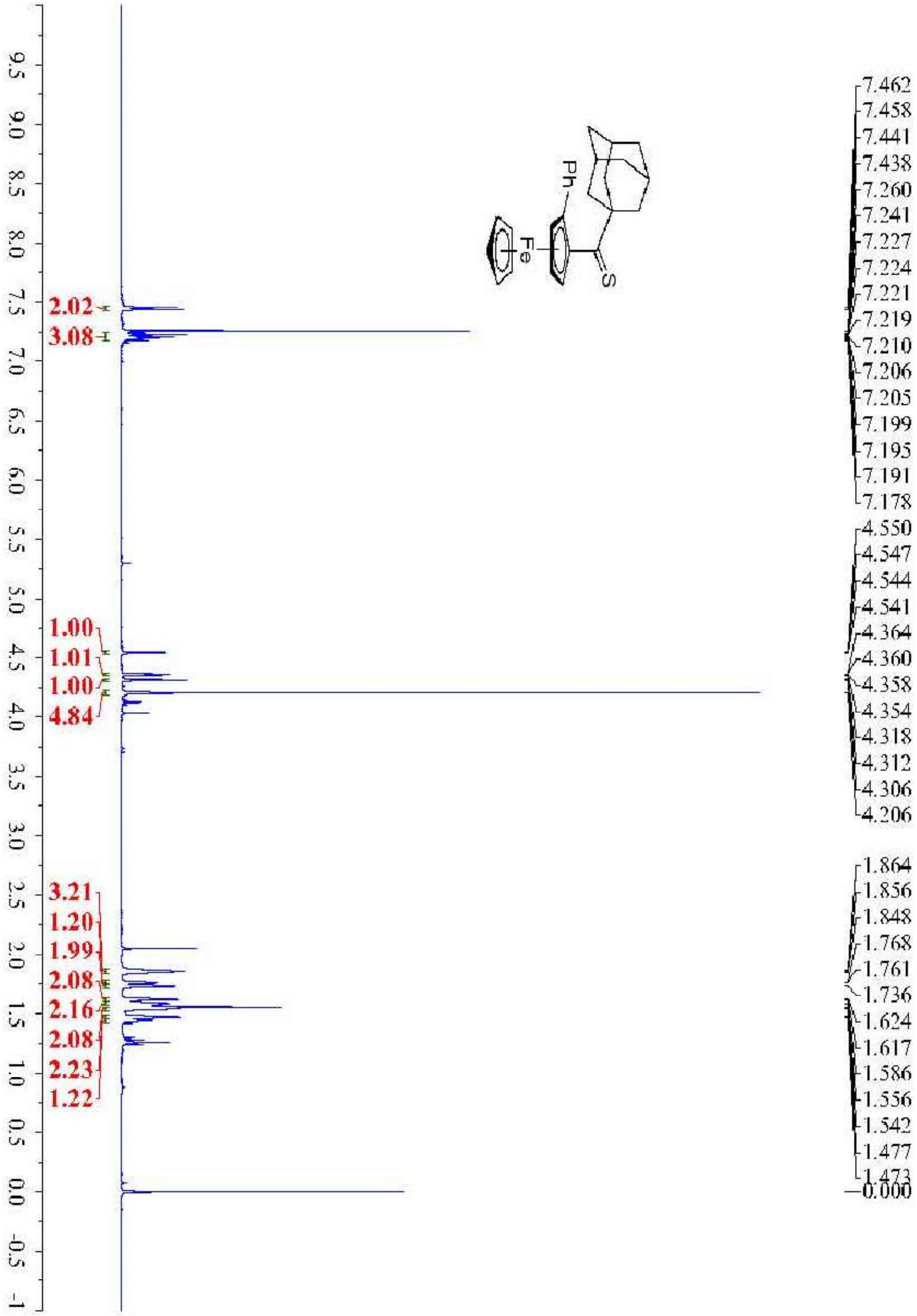

Supplementary Figure 42. HPLC analysis **3s**

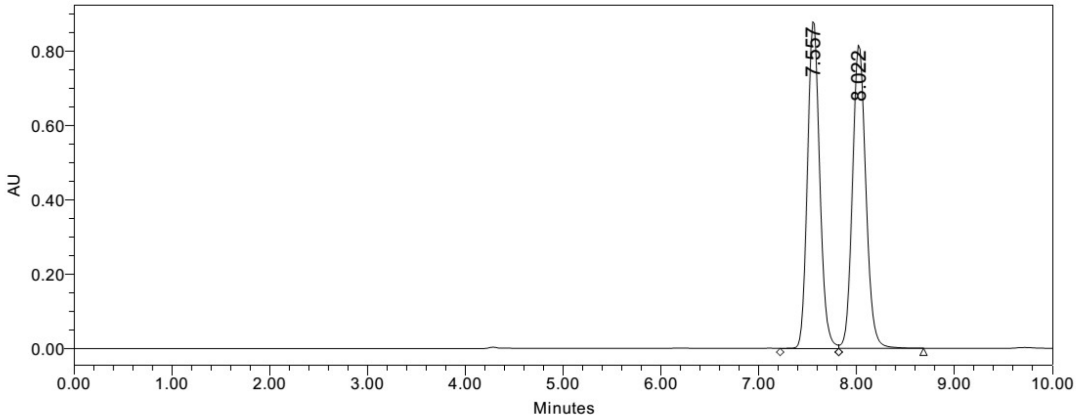

	RT	Area	% Area	Height
1	7.557	7634485	49.62	882976
2	8.022	7751784	50.38	817106

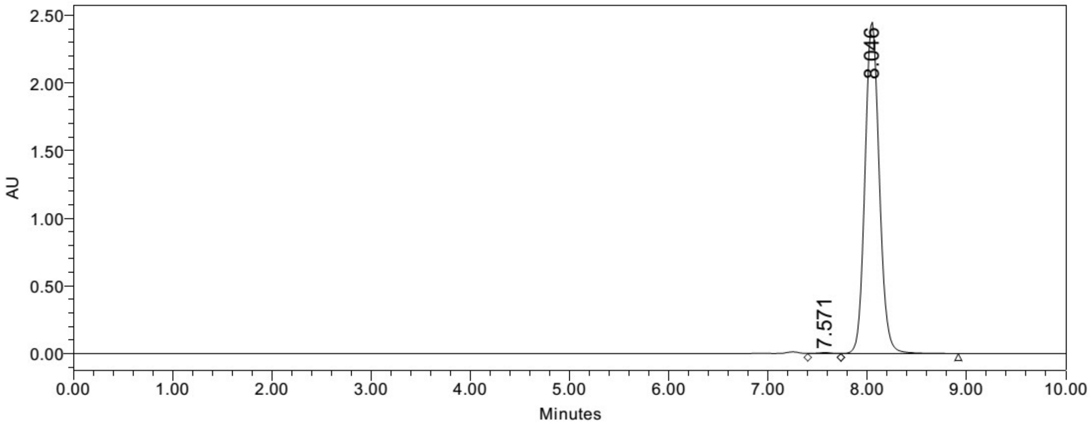

	RT	Area	% Area	Height
1	7.571	66586	0.27	6806
2	8.046	24571330	99.73	2453127

Supplementary Figure 43. ¹H NMR spectra of **4**

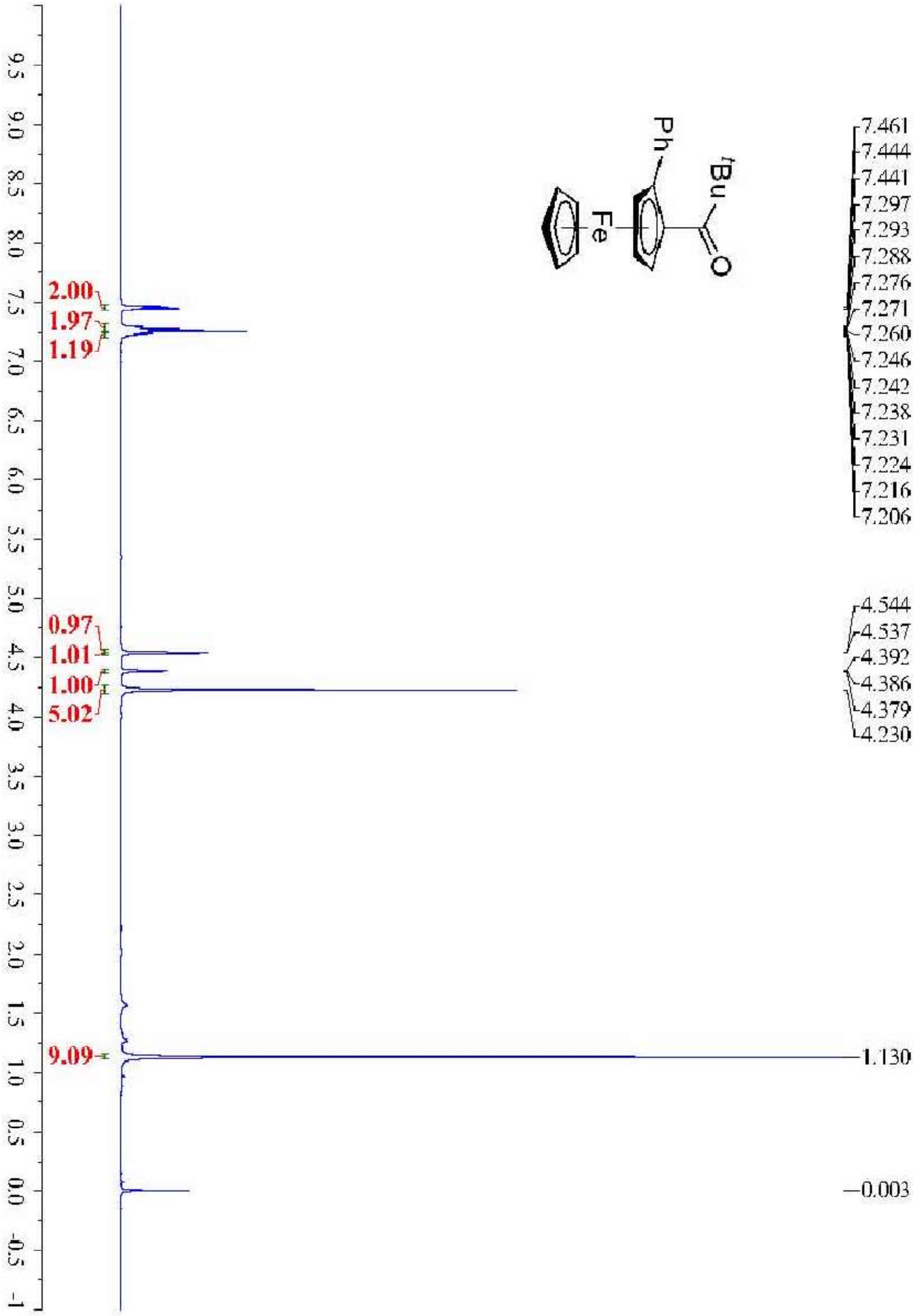

Supplementary Figure 44. HPLC analysis **4**

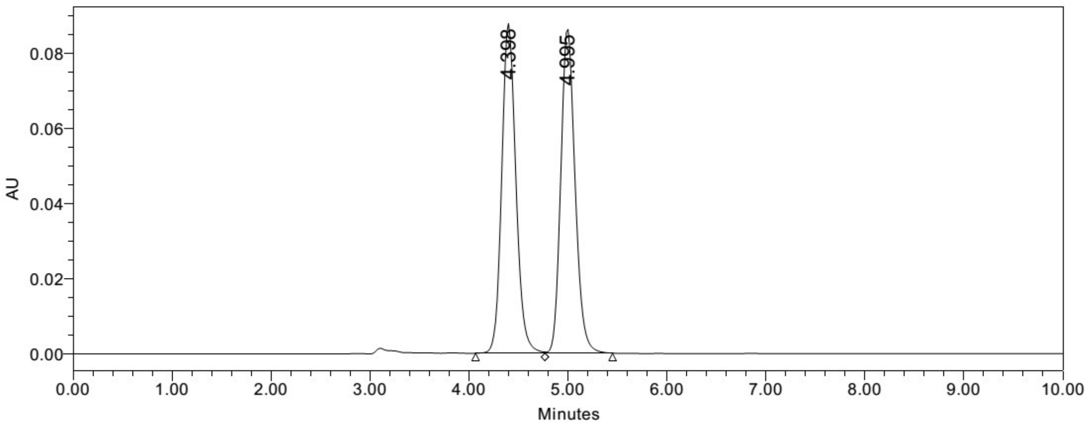

	RT	Area	% Area	Height
1	4.398	867104	50.05	87621
2	4.995	865289	49.95	86303

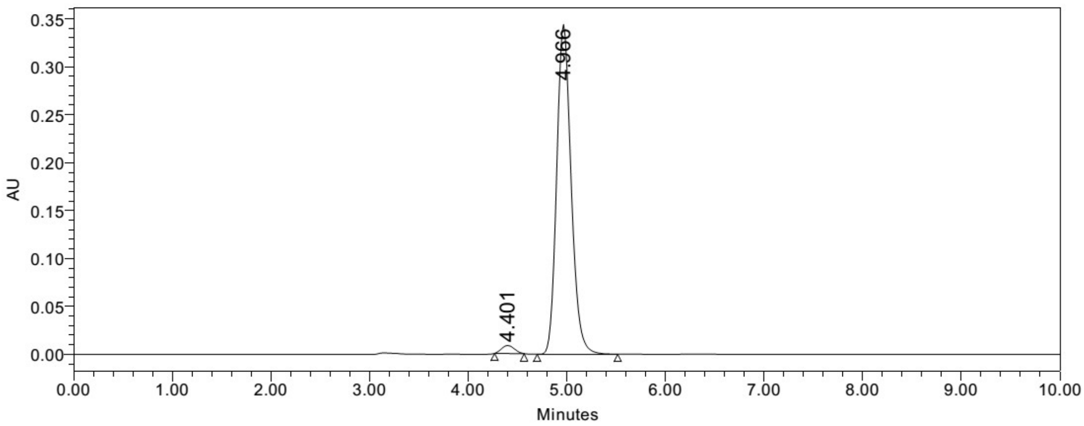

	RT	Area	% Area	Height
1	4.401	74953	2.09	8354
2	4.966	3502903	97.91	343847

Supplementary References

(1) Cai, Z.-J.; Liu, C.-X.; Gu, Q.; You, S.-L. Thioketone Directed Palladium(II)-Catalyzed C-H Arylation of Ferrocenes with Aryl Boronic Acids. *Angew. Chem. Int. Ed.* **2018**, *57*, 1296.

(2) (a) Klussmann, M.; Ratjen, L.; Hoffmann, S.; Wakchaure, V.; Goddard, R.; List, B. Synthesis of TRIP and Analysis of Phosphate Salt Impurities. *Synlett* **2010**, *14*, 2189; (b) Kashikura, S.; Mori, K.; Akiyama, T. Chiral Phosphoric Acid Catalyzed Enantioselective Synthesis of β-Amino-α,α-difluoro Carbonyl Compounds. *Org. Lett.* **2011**, *13*, 1860; (c) Michailidis, F. R.; Guenee, L.; Alexakis, A. Enantioselective Organocatalytic Iodination-Initiated Wagner-Meerwein Rearrangement. *Org. Lett.* **2013**, *15*, 5890.

(3) Feringa, B. L.; Pineschi, M.; Arnold, L. A.; Imbos, R.; de Vries, A. H. M. Highly Enantioselective Catalytic Conjugate Addition and Tandem Conjugate Addition-Aldol Reactions of Organozinc Reagents. *Angew. Chem., Int. Ed. Engl.* **1997**, *36*, 2620.

(4) Defieber, C.; Ariger, M. A.; Moriel, P.; Carreira, E. M. Iridium-Catalyzed Synthesis of Primary Allylic Amines from Allylic Alcohols: Sulfamic Acid as Ammonia Equivalent. *Angew. Chem. Int. Ed.* **2007**, *46*, 3139.

(5) Greßies, S.; Klauck, F. J. R.; Kim, J. H.; Daniliuc, C. G.; Glorius, F. Ligand-Enabled Enantioselective C–H Activation of Tetrahydroquinolines and Saturated Aza-Heterocycles by Rh^{I}. *Angew. Chem. Int. Ed*. **2018**, *57*, 9950.

(6) Fu, Y.; Hou, G.-H.; Xie, J.-H.; Xing, L.; Wang, L.-X.; Zhou, Q.-L. Synthesis of Monodentate Chiral Spiro Phosphonites and the Electronic Effect of Ligand in Asymmetric Hydrogenation. *J. Org. Chem.* **2004**, *69*, 8157.
